# WNT activity reveals context-specific genetic effects on gene regulation in neural progenitors

**DOI:** 10.1101/2023.02.07.527357

**Published:** 2023-02-07

**Authors:** Nana Matoba, Brandon D Le, Jordan M Valone, Justin M Wolter, Jessica Mory, Dan Liang, Nil Aygün, K Alaine Broadaway, Marielle L Bond, Karen L Mohlke, Mark J Zylka, Michael I Love, Jason L Stein

**Affiliations:** 1 Department of Genetics, University of North Carolina at Chapel Hill; Chapel Hill, NC, USA; 2 UNC Neuroscience Center, University of North Carolina at Chapel Hill; Chapel Hill, NC, USA; 3 Department of Cell Biology and Physiology, University of North Carolina at Chapel Hill; Chapel Hill, NC, USA; 4 Carolina Institute for Developmental Disabilities; Carrboro, NC, USA; 5 Department of Biostatistics, University of North Carolina at Chapel Hill; Chapel Hill, NC, USA

## Abstract

Molecular quantitative trait loci (QTLs) generate mechanistic hypotheses explaining how genetic variation impacts brain traits, but gene regulatory effects in bulk-post mortem brain tissues are undetected at many brain trait-associated loci. We hypothesized that the function of genetic variants may only be apparent in specific contexts, such as during stimulation of developmental signaling pathways. We measured chromatin accessibility and gene expression after activation of the canonical Wnt pathway in primary human neural progenitor cells from 82 donors. Brain structure and neuropsychiatric disorder-associated variants were enriched within Wnt-responsive regulatory elements. Thousands of context-specific molecular QTLs were identified during Wnt stimulation. Context-specific molecular QTLs increased the number of colocalizations by up to 70% and led to the nomination of developmental gene regulatory mechanisms underlying complex brain traits.

## One Sentence Summary

Stimulation of the canonical Wnt pathway reveals the regulatory function of brain-trait associated genetic variants.

Common genetic variation associated with brain-relevant traits and risk for neuropsychiatric disorders have been identified and replicated, providing a molecular basis for understanding inter-individual variation in brain structure, function, and behavior (1, 2). However, brain-trait associated loci are mostly found in non-coding regions without clear mechanisms of action. Gene regulatory mechanisms of non-coding loci are inferred using datasets mapping the effects of genetic variation on regulatory element activity, marked by accessible chromatin peaks (chromatin accessibility quantitative trait loci or caQTL), or gene expression (eQTL) (3). Gene regulatory associated loci (ca/eQTLs) measured in bulk post-mortem tissue have explained mechanisms for a subset of brain-trait associated loci through sharing, or colocalization, of causal variants (4–6). Yet, many brain-trait associated variants do not have detectable gene regulatory function in bulk post-mortem brain tissue, leading to the question of where the ‘missing regulation’ linking trait-associated variants to gene expression lies (4, 7, 8).

One potential solution is that variants impact the accessibility of regulatory elements or the expression of target genes only in specific contexts or when activated by certain stimuli (response-QTLs), and therefore are unlikely to be observed in bulk post-mortem tissue (9). The context specificity of genetic variant function, despite identical genetic sequence (excluding somatic mutations) being present in every cell, may be explained through the action of transcription factors only expressed, activated, or translocated to the nucleus within certain cell-types or during stimulation. Context-specific genetic effects on gene expression are consistent with the observation that only ~30% of genetic variants affecting gene expression also affect unstimulated chromatin accessibility, suggesting that some regulatory elements are primed to impact gene expression when a stimulus leads to the activation of additional transcription factors (9, 10). Recent studies characterizing cell-type specific ca/eQTLs highlight the importance of cellular context by revealing novel brain trait colocalizations undetected in bulk tissues (10–13). We hypothesized that the stimulation of a developmental signaling pathway in a homogeneous neural cell type will reveal previously undetected functions of genetic variation and explain some of the ‘missing regulation’ for brain-trait associated loci.

**Fig. 1.**
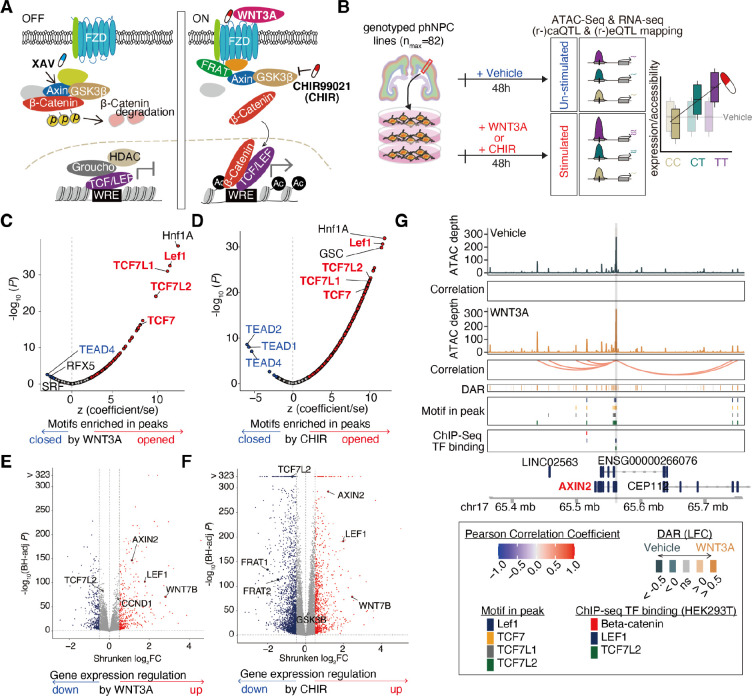
Gene regulatory changes induced by WNT stimulus (**A**) Cartoon of the canonical WNT signaling pathway. (**B**) Schematic of study design. Enrichment of TF motifs in WNT3A-responsive (**C**) or CHIR-responsive (**D**) chromatin accessibility peaks. Z-scores reflect scaled enrichment scores (x-axis), and −log10(P-values) depict the significance of enrichment (y-axis). TFBS motifs significantly enriched in peaks opening or closing due to the stimulus are represented by red and blue points, respectively. (**D**) Volcano plots show gene expression changes induced by exposure to WNT3A (**E**) or CHIR (**F**). Genes with significantly increased or decreased expression (DEGs) are represented by red and blue points, respectively. (**G**) WREs significantly correlated to *AXIN2* expression during WNT3A stimulation. DAR: Differentially accessible chromatin regions measured by ATAC-seq. DARs and TSSs in the region overlap TCF/LEF motifs and Wnt-relevant TF binding (27).

We evaluated context-specific effects of genetic variation in a population of primary human neural progenitor cells (hNPCs), a developmental cell type with regulatory elements strongly enriched in genetic association signals for multiple brain-related traits and neuropsychiatric disorders (10, 11, 14). We measured chromatin accessibility and gene expression in hNPCs following stimulation of the canonical Wnt pathway, which is known to impact neural progenitor proliferation and cortical patterning and is associated with the inter-individual differences in complex brain traits (2, 15–22). Wnt stimulation stabilizes cytoplasmic β-catenin, allowing it to translocate into the nucleus where it opens chromatin by displacing the repressor Groucho at TCF/LEF binding sites and promotes the expression of Wnt target genes (Fig. 1A) (23). Our results characterize context-specific genetic effects in hNPCs that provide novel insights into neurodevelopmental gene regulatory mechanisms underlying brain trait-associated loci.

## Results

### Wnt stimuli impact gene regulation

We cultured and maintained a population of previously genotyped multi-ancestry hNPC donors as proliferative neural progenitors (10, 24) (Fig. 1B, fig. S1). Either 5nM WNT3A, 2.5μM CHIR (CHIR99021, also known as CT99021), a potent GSK3β inhibitor and Wnt activator (25), or vehicle were added to the hNPCs for 48 hours. These exposures were selected to activate canonical Wnt signaling (26) and increase proliferation in hNPCs (fig. S2). Following 48h stimulation, we performed ATAC-seq and RNA-seq for all samples, including 2–6 replicates for each of six randomly selected donors to evaluate technical reproducibility (fig. S3–5). After quality checks and selection of one technical replicate from each donor-condition pair (see Methods), we detected expression of 15,762 protein-coding genes and 7,695 lncRNAs from 242 RNA-seq samples (*n*_vehicle_=79, *n*_WNT3A_=82, *n*_CHIR_=81) and chromatin accessibility at 172,887 peaks from 222 ATAC-seq samples (*n*_vehicle_=76, *n*_WNT3A_=68, *n*_CHIR_=78).

To determine the gene regulatory impacts of Wnt stimulation on human neural progenitors, we performed differential analyses of chromatin accessibility and gene expression between WNT3A- or CHIR-stimulated conditions compared to vehicle control. Stimulation by WNT3A or CHIR revealed 21,383 unique differentially accessible chromatin regions (Wnt-responsive elements or WREs; FDR Benjamini-Hochberg-adjusted *P* < 0.1, |LFC| > 0.5; WNT3A vs Vehicle (62 pairs): 4,819 WREs; CHIR vs Vehicle (72 pairs): 20,179 WREs; fig. S6A, table S1). We anticipated Wnt stimulation would increase chromatin accessibility at TCF/LEF binding sites as β-Catenin displaces the chromatin condenser Groucho (23). Consistent with these expectations, WREs opening due to Wnt stimulation were strongly enriched with TCF7, TCF7L1/2, and Lef1 motifs Fig 1C–D, table S2). β-Catenin, Lef1, and TCF7L2 binding sites defined by ChIP-seq in HEK293T cells (27) also had significantly greater overlap with WRE opening as compared to closing (fig. S7). Additional enrichment of HNF1a motifs within WREs (Fig. 1C–D) implies a coregulatory relationship with TCF/LEF, as has been previously described in cancer cells (28). Interestingly, binding motifs of non-canonical Wnt signaling such as TEAD4 (29) were enriched in WREs that closed in response to Wnt stimulation (Fig. 1C–D), suggesting an antagonistic relationship between canonical and non-canonical WREs. These results show that Wnt stimulation in human neural progenitors modulates known downstream DNA-binding protein effectors in expected directions and defines a set of human brain-developmental WREs.

We detected a total of 3,254 unique Wnt-responsive differentially expressed genes (DEGs) across the two Wnt-stimulating conditions (DEGs, FDR-adjusted *P* < 0.1; |LFC| > 0.5; WNT3A vs Vehicle (75 pairs): 762 DEGs; CHIR vs Vehicle (74 pairs): 3,031 DEGs (Fig. 1E–F, fig. S6B, table S3)). DEGs included known components of the Wnt pathway such as *LEF1* and *AXIN2*, confirming that Wnt stimulation leads to autoregulation of the Wnt pathway (30, 31). Pathway enrichment analysis of DEGs showed that those upregulated in response to Wnt stimulus were over-represented in Wnt-related pathways such as “TCF dependent signaling in response to WNT” (FDR-adjusted *P* = 3.81×10^−7^ and 4.69×10^−7^, for WNT3A vs Vehicle, or CHIR vs Vehicle, respectively), as expected (table S3–4). The Wnt pathway is also known to increase proliferation (fig. S2), and indeed, upregulated DEGs were enriched in “Cell Cycle [REAC]” related genes (FDR-adjusted *P* = 7.17 × 10^−51^ and 7.97 × 10^−45^, for WNT3A vs Vehicle, or CHIR vs Vehicle, respectively) (32). Additionally, Cyclin D1 (*CCND1*), a known target gene of WNT stimulation and a key factor regulating cell cycle progression (33), was significantly upregulated under WNT3A stimulation (LFC = 0.44, FDR-adjusted *P* = 1.63 × 10^−67^, Fig. 1E).

Activation of the Wnt-signaling pathway alters gene expression patterns that modulate NPC cellular behaviors such as proliferation and differentiation to shape brain development (34, 35). To identify novel WREs that may impact gene expression, we estimated the correlation between chromatin accessibility and gene expression for proximal gene-peak pairs (+/−1MB from the transcription start site (TSS)) to link regulatory elements to the genes they regulate. We found that across stimulation and vehicle conditions, over 5% of peaks are significantly correlated with nearby genes and over 12% of genes are significantly correlated with nearby peaks (FDR-adjusted *P* < 0.1, table S5–6). On average, 83% of gene-peak pairs showed a positive correlation, supporting the idea that opening chromatin usually increases, while restricting chromatin accessibility usually decreases, the expression of target genes. Wnt stimulation revealed 12,643 enhancer-gene pairs not detected in the vehicle condition, while 6,461 were lost (fig. S8). For example, *AXIN2*, a gene known to be upregulated by canonical Wnt signaling across tissues (23, 36), linked to 11 or 8 peaks each harboring TCF/LEF binding sites under WNT3A or CHIR condition, respectively, yet had no significantly correlated peak-gene links found in the vehicle condition (Fig. 1G). These data suggest that new enhancers are recruited to regulate gene expression during Wnt stimulation.

While both WNT3A and CHIR stimulate the Wnt pathway, they induced different effects on gene expression that are consistent with their distinct mechanisms of action. CHIR yielded considerably more WREs and DEGs as compared to WNT3A, suggesting that this potent small molecule inhibitor of GSK3*β* induces more gene regulatory changes as compared to the endogenous ligand at their respective concentrations, possibly because CHIR acts downstream of WNT3A where it may more directly affect target gene expression, though concentration differences between the two stimuli make direct comparisons difficult (Fig. 1E–F; fig. S6). The expression level of *GSK3β* is upregulated in progenitor cells stimulated with CHIR (LFC = 0.15, FDR-adjusted *P* = 4.64×10^−46^), but not WNT3A (LFC = 0, FDR-adjusted *P* = 0.97; differential impact estimated by interaction term = 9.14×10^−26^). This suggests that GSK3*β* inhibition by CHIR triggers a compensatory gene expression response that is not induced by Wnt signaling activated via the endogenous ligand (Fig. 1A) (37).

To test the specificity of Wnt pathway stimulations, we compared gene expression during simultaneous activation and downstream inhibition of the pathway (WNT3A + XAV (38)) with WNT3A activation alone in 6 hNPC donor lines (fig. S9A). As expected, inhibition of the Wnt signaling pathway suppressed expression of genes upregulated by Wnt stimulation as compared to vehicle (r = −0.59; *P* < 1 × 10^−323^; fig. S9B). For example, *LEF1* expression increased in response to WNT3A stimulation, and decreased following inhibition of the WNT pathway. These opposing effects provide further support that the observed gene expression changes are caused by induction of canonical Wnt signaling.

### Wnt-responsive genes and regulatory elements contribute to inter-individual differences in brain traits

**Fig. 2.**
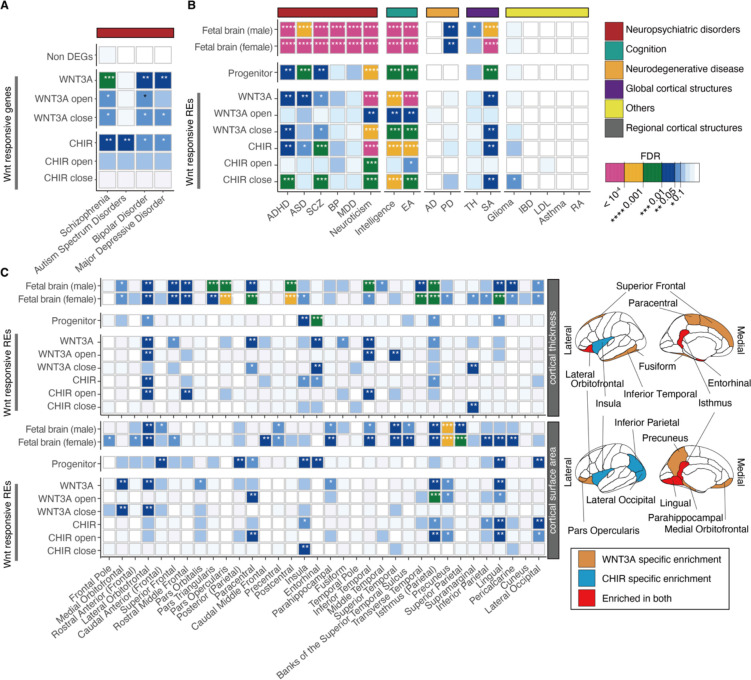
Contribution of Wnt-responsive regulatory elements to the heritability of brain traits (**A**) Enrichment of Wnt-responsive genes within neuropsychiatric disorder risk gene sets from the DisGeNET database (39). (**B**) Contribution of WREs to brain-related trait heritability evaluated by S-LDSC. Traits are grouped by category. (**C**) Contribution of WREs to the heritability of adult cortical thickness and cortical surface area traits across regions (left). Brain regions with significant enrichment of WREs impacting cortical thickness (top) or cortical surface area (bottom) traits (right). The *P* values indicated by color in (**B**-**C**) denote whether the WREs contribute significantly to SNP heritability after controlling for other annotations including elements in baseline model and/or non-WREs). * indicates enrichments with FDR < 0.1. ADHD: Attention deficit hyperactivity disorder, ASD: Autism spectrum disorder, SCZ: Schizophrenia, BP: Bipolar disorder, MDD: Major depressive disorder, EA: Educational attainment, AD: Alzheimer’s disease, PD: Parkinson’s disease, TH: Average cortical thickness, SA: Cortical surface area, IBD: Inflammatory bowel disease, LDL: low-density lipoprotein, RA: Rheumatoid arthritis (see table S8 for references).

Previous studies suggest that genes related to the Wnt pathway are mutated or differentially expressed in individuals with neuropsychiatric disorders (18–22). We sought to determine whether Wnt-responsive genes further support these disease associations by testing for enrichment of Wnt-responsive DEGs in sets of brain-related disease-associated genes using curated gene-disease information from the DisGeNET database (39). We found enrichment of Wnt-responsive DEGs among schizophrenia and ASD risk genes, (Fig. 2A, table S7) while genes not significantly differentially expressed after Wnt stimulation did not show a detectable enrichment among brain-related disease associated genes (40). This implies that alteration in the function of Wnt-responsive genes contributes to risk for neurodevelopmental disorders.

We next explored whether common genetic variants within WREs contribute to brain-related traits (table S8). By performing stratified LD score regression (S-LDSC) accounting for the baseline-model (41, 42), we replicated previous findings that regulatory elements in fetal brain tissues or hNPCs contribute to the heritability of neuropsychiatric disorders and brain-related traits (14, 43) (FDR-adjusted *P* < 0.1; Fig. 2B, table S9). We then applied S-LDSC to WREs and found that they contribute to the heritability of schizophrenia, ADHD, ASD, and glioma, as well as inter-individual differences in intelligence, even when controlling for the effects of non-differentially accessible peaks (Fig. 2B, table S9). We also found that common variants within WREs significantly contribute to inter-individual differences in global cortical surface area, but not cortical thickness, consistent with Wnt regulating progenitor proliferation and the predictions of the radial unit hypothesis (15, 44). We further estimated partitioned heritability enrichment for regional cortical surface area and thickness traits. We observed regional specificity where heritability was enriched in WREs for the surface area of regions such as lingual gyrus and isthmus of the cingulate (Fig. 2C). Interestingly, the heritability of cortical thickness was also enriched in WREs within regions including lateral orbitofrontal and the isthmus of the cingulate. We did not detect partitioned heritability enrichment for neurodegenerative disorders (Alzheimer’s disease and Parkinson’s disease) or non-brain related traits (Irritable Bowel Disease, Low-Density Lipoprotein, Asthma, and Rheumatoid Arthritis), showing the specificity of these enrichments. In summary, common variants within Wnt-responsive genes and regulatory elements contribute to inter-individual differences in brain structure, neuropsychiatric disease risk, and cognitive ability.

### Context-specific genetic effects on chromatin accessibility and gene expression

**Fig. 3.**
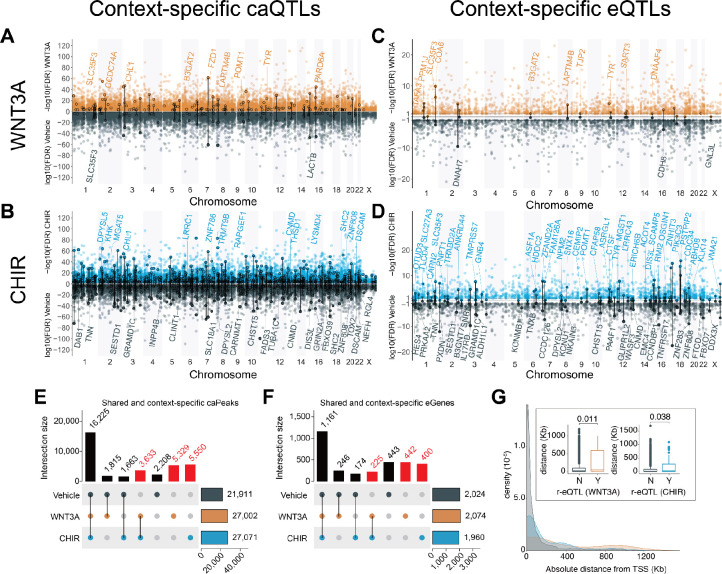
Context-specific genetic effects on chromatin accessibility and gene expression. Miami plots depict significant caQTLs (**A**, **B**) or eQTLs (**C**, **D**) detected under WNT3A (orange) (**A**, **C**), CHIR (blue) (**B**, **D**) or vehicle conditions (gray) across the genome. Circled variants denote significant genotype-by-condition interaction effects (r-QTLs). Protein-coding eGenes overlapping r-caQTLs and eGenes highlighted in subsequent figures are labeled. The number of context-specific caPeaks (**E**) or eGenes (**F**) shared across vehicle, WNT3A, and CHIR conditions. Columns labeled in red represent caPeaks or eGenes r only detected in Wnt-stimulated conditions. (**G**) Distributions of absolute genomic distances between eQTLs and their target gene’s transcriptional sites (TSS). Boxplots summarizing these distances are shown in the inset (P_WNT3A_Veh_ = 0.011; P_CHIR_Veh_ = 0.038).

Because Wnt-responsive gene expression and regulatory elements contribute to inter-individual differences in brain traits, we sought to identify single nucleotide polymorphisms (SNPs) affecting gene regulation during Wnt-stimulation. We mapped chromatin accessibility and expression quantitative trait loci (ca/eQTL) using stringent control for known and unknown confounding and use of a hierarchical multiple testing correction (Methods). We identified over 43,000 caQTLs (caSNP-caPeak pairs) in each condition regulating 36,423 unique caPeaks (FDR-adjusted P < 0.1; number of caQTL pairs = 43,664 Vehicle; 57,718 WNT3A; 57,581 CHIR; Fig. 3A–B). We also identified ~2,000 eQTL (eSNP-eGene pairs) in each condition regulating 3,089 unique eGenes (FDR-adjusted *P* < 0.1; number of eQTL pairs = 2,025 Vehicle; 2,075 WNT3A; 1,961 CHIR) (Fig. 3C–D, tables S10–11). The observed effect size of vehicle ca/eQTLs in this study strongly correlated with ca/eQTL effect sizes using largely overlapping hNPC samples cultured for previous studies (10, 11), indicating our findings are highly reproducible (caQTL r = 0.93, *P* < 1 × 10^−323^; eQTL r = 0.89, *P* < 1×10^−323^; fig. S10). When the same SNP was identified as both a caQTL and an eQTL for a given stimulus, we observed strong correlation between effect sizes on chromatin accessibility and gene expression, also as found in our previous work (fig. S11).

We observed a 66.2% increase in caPeaks and a 52.7% increase in eGenes detected in the Wnt stimulated states as compared to vehicle (Fig. 3E–F). The directionality and magnitude of QTL effect sizes between stimulated and unstimulated conditions were generally consistent, but nevertheless many QTLs exhibited differential effects in the stimulated condition consistent with the increase in detection of caPeaks and eGenes (fig. S12). These results show that Wnt stimulation reveals context-specific genetic effects on gene regulation previously undetected in unstimulated cells.

**Fig 4.**
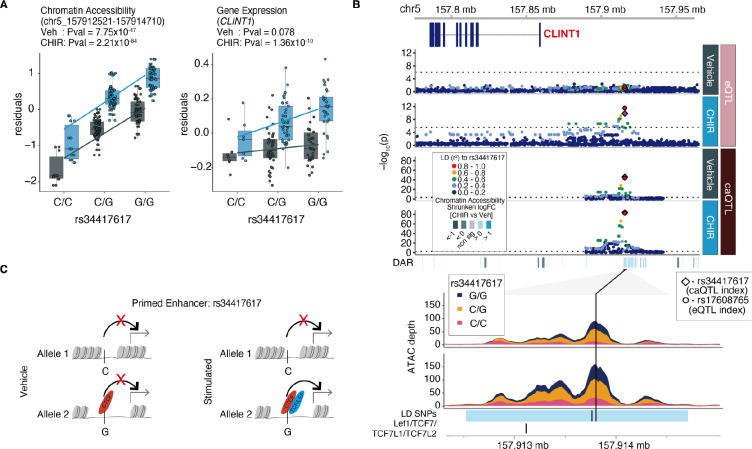
Enhancer priming mediated by stimulus-specific TF binding (**A**) Allelic effects of rs34417617 on chromatin accessibility of WRE (chr5:157912521–157914710) (left) and *CLINT1* expression (right). (**B**) Regional association plots at the *CLINT1* locus. From top to bottom: Genomic coordinates, gene models, eQTL and caQTL *P* values for vehicle and CHIR-stimulated conditions, ATAC-seq coverage showing differential chromatin accessibility, with SNPs linked by LD (r^2^ > 0.8) and TCF/LEF elements annotated. (**C**) Putative mechanism for rs34417617 regulating chromatin accessibility and gene expression.

We detected 253 instances of enhancer priming, where a genetic variant is associated with chromatin accessibility and inferred differences in TF binding in both vehicle and stimulated conditions, but only leads to changes in gene expression in the stimulated condition, likely due to recruitment of additional stimulus-specific TFs. For example, at a caPeak 53 kb from the TSS of *CLINT1*, we detected two high LD SNPs within the peak strongly associated with chromatin accessibility in both vehicle and under CHIR stimulation, one of which disrupts the CTCF motif (table S12). But, this locus was only associated with gene expression under CHIR stimulation, presumably due to the recruitment of TCF/LEF during Wnt stimulation to the motifs present in this peak (Fig. 4A–C).

Since we observed shared and distinct effects of genetic variants on gene regulation across stimulation conditions, we hypothesized two common interpretable scenarios out of many possibilities. (1) Wnt-activation increased power to detect condition-specific ca/eQTLs due to changes in accessibility or expression, or (2) Wnt activation alters the function of a genetic variant in regulating chromatin accessibility or target gene expression by revealing a differential genotypic effect as compared to vehicle. To test the latter hypothesis, we performed a genotype-by-condition interaction test for all independent QTLs, which showed the main effects on chromatin accessibility or their target eGenes, and detected 22 and 102 significant r-eQTLs and 291 and 1,800 r-caQTLs in WNT3A, CHIR, respectively (labeled in Fig. 3, fig S12, tables S13–14). Notably, r-eQTLs were more distal to the TSS of the regulated eGene as compared to non-r-eQTLs (Fig. 3G). This finding is consistent with the idea that context-specific regulatory elements are farther from the genes they regulate than are non-context-specific regulatory elements.

### Identifying context-dependent gene regulatory loci shared with brain-related GWAS traits

**Fig 5.**
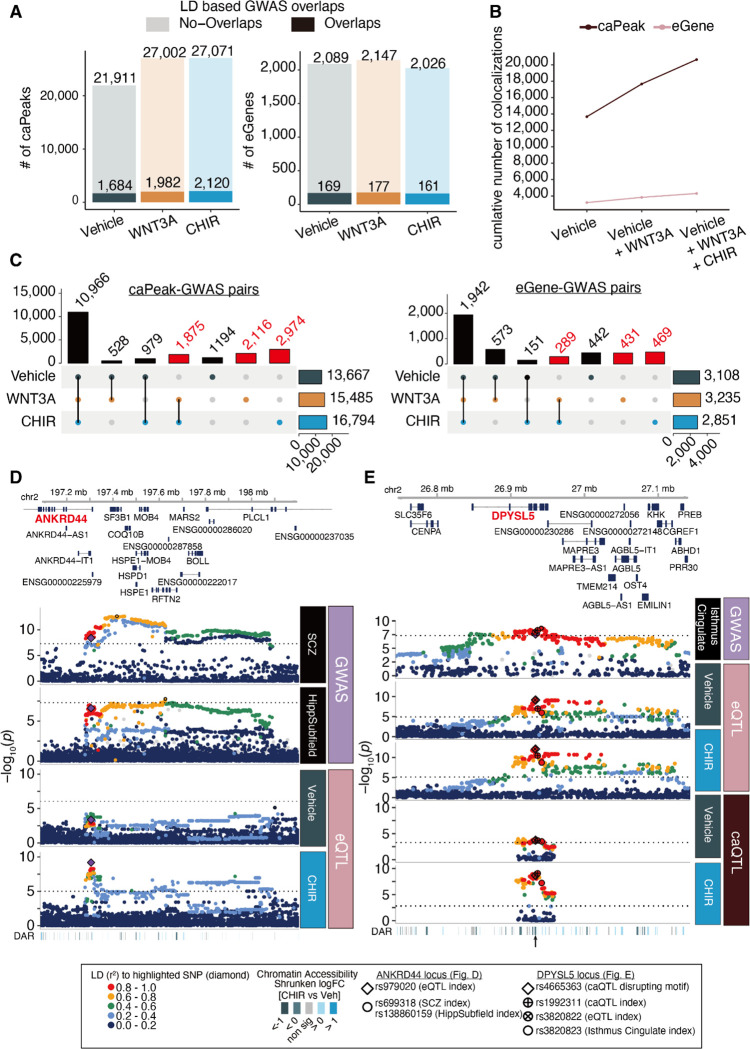
Using WNT stimulus-specific gene regulation to inform mechanisms underlying complex brain traits (**A**) The number of caPeaks (left) or eGenes (right) overlapping brain-related GWAS loci defined by moderate LD (*r*^*2*^ > 0.6 in either 1KG EUR population or our study) across vehicle, WNT3A and CHIR conditions. (**B**) The cumulative number of colocalized caPeaks or eGenes increases across stimulation conditions. (**C**) Shared or condition-specific caPeaks (left) or eGenes (right) colocalized with brain-related GWAS traits in each condition. Columns labeled in red indicate colocalizations only detected in Wnt-stimulated conditions. (**D**) Regional association plot depicting colocalization of schizophrenia (top panel) and the volume of a hippocampal subfield (presubiculum body, left hemisphere) GWAS with a CHIR-responsive eQTL modulating *ANKRD44* expression (rs979020-T, CHIR vs vehicle interaction FDR-adjusted *P* = 0.09). From top to bottom: Genomic coordinates and gene models, *P* values for brain-related GWAS, *P* values for condition-specific QTLs discovered in this study, and differentially accessible regions (DAR) within the locus. Differences in the patterns of association are likely due to population differences in LD between the GWAS and QTL studies. (**E**) Regional association plot depicting colocalization of average thickness of isthmus cingulate GWAS with a CHIR-responsive eQTL modulating *DPYSL5* expression and a CHIR-responsive caQTL (rs1992311, interaction FDR-adjusted *P* = 0.041; chr2:26932281–26934470, in an intron of *DPYSL5*), arranged as in (**D**).

Partitioned heritability analysis revealed that GWAS loci associated with neuropsychiatric disorder risk and brain structures are enriched at WREs, demonstrating that these elements contribute broadly to brain phenotypes (Fig. 2). However, enrichments do not nominate specific genes and variants underlying these contributions. In order to identify variants and putative gene regulatory mechanisms to explain brain trait GWAS loci including brain structure, function, neuropsychiatric disorders, and cognitive ability, we examined caQTLs and eQTLs with LD-overlap to GWAS loci (table S8). Based on this analysis, we identified 1,684 regulatory elements and 169 genes involved in brain-traits in the vehicle condition (Fig. 5A). The use of stimulated conditions increased the number of brain-trait associated peaks by 72.2% and genes by 57.3% (Fig. 5B), demonstrating that they may explain some of the ‘missing regulation’ underlying GWAS loci. 6,965 caPeak-GWAS pairs and 1,189 eGene-GWAS pairs were unique to stimulated conditions (Fig. 5C, table S16–17).

We highlight two stimulus-specific colocalizations, confirmed by conditional analysis, with the r-e/ca-QTLs. First, a CHIR r-eQTL modulating expression of *ANKRD44* (rs979020-T, Fig. 5D, fig. S13A) colocalized with schizophrenia GWAS and the volume of the left presubiculum body hippocampal subfield (45, 46). *ANKRD44* encodes an ankyrin repeat domain functioning as a regulatory subunit of protein phosphatase-6 (PP6), an enzyme that regulates the cell cycle and suppresses NF-kB signaling, a pathway known to engage in cross-talk with Wnt signaling (47–50). The T allele of rs979020 is associated with increased expression of ANKRD44, decreased risk of schizophrenia, and reduced volume of the hippocampal. This colocalization underscores the connection between decreased hippocampal volume and schizophrenia (51, 52) and suggests Wnt-responsive regulation of *ANKRD44* in neural progenitors plays a role in the expression of these traits. A second example is the colocalization of a CHIR r-caQTL (rs1992311; Fig. 5E, fig. S14) with a CHIR-responsive *DPYSL5* eQTL signal and a GWAS of average thickness of the isthmus cingulate region. *DPYSL5*, also known as *CRMP5*, has shown to be a negative regulator of neural progenitor proliferation (*53*). A Pou5f1::Sox2 motif is predicted to be disrupted by the caSNP in this caPeak (rs4665363-G; fig. S14E) which is likely modulated in the stimulation condition by TCF/LEF binding to motifs present in the same peak. This suggests that rs4665363 is a putative causal variant altering chromatin accessibility and downregulating *DPYSL5* expression, which may lead to increase of average thickness of the isthmus cingulate. We also observed stimulus-specific colocalizations supported by eCAVIAR, including *FADS3* with bipolar disorder (54), and *ENO4* with variants associated with regional cortical surface area (2) (fig. S15–S17). These results highlight that fine-mapping via integrating r-QTLs and GWAS traits allow us to find putative regulatory mechanisms that impact brain-related GWAS traits.

## Discussion

In this study, we stimulated the Wnt pathway in a library of genetically diverse human neural progenitor cells. We identified context-specific gene regulatory relationships and revealed novel effects of common genetic variants not detected in unstimulated states. We show that context-specific genetic effects reveal some of the ‘missing regulation’ underlying GWAS signals for brain structure and psychiatric disorders. Our results imply that genetic variants have function during early neurodevelopmental patterning that lead to differences in adult brain and behavioral traits. Future expansion of this approach in large populations of primary human neural progenitor cells or induced pluripotent stem cells will enable testing gene-by-environment interactions in a dish. We expect that stimulation of additional pathways relevant to brain development (55) or modulating neuronal activity (56), may reveal additional genetic effects that are masked in QTL studies conducted in bulk post-mortem tissue. A similar study design will likely be useful to understand how genetic variation influences disease risk after exposure to environmental insults, or alters cellular and molecular responses to clinically useful drugs (57, 58).

## Supplementary Material

Supplement 1

Supplement 2

Supplement 3

Supplement 4

Supplement 5

Supplement 6

Supplement 7

Supplement 8

Supplement 9

Supplement 10

Supplement 11

Supplement 12

Supplement 13

Supplement 14

Supplement 15

Supplement 16

Supplement 17

## Data Availability

All code used in analyses and data including full summary statistics except that which is provided in supplementary tables will be available upon publication at https://bitbucket.org/steinlabunc/wnt-rqtls/
