# Supplementary material for: Wnt activity reveals context-specific genetic effects on gene regulation in neural progenitors
Source: bioRxiv. 2023 Apr 19:2023.02.07.527357. Originally published 2023 Feb 7. Preprint. [Version 2] doi: 10.1101/2023.02.07.527357 (PMC9934631; doi:10.1101/2023.02.07.527357)
Supplement: Supplement 1 [file media-1.pdf]

Supplementary Information

Supplementary Figures **3**

Supplementary Figure 1| Multidimensional scaling analysis of genotype data 3
Supplementary Figure 2| Effects of CHIR and WNT3A on proliferation and Wnt activity 4
Supplementary Figure 3| Technical reproducibility of RNA-seq and ATAC-seq 6
Supplementary Figure 4| ATAC-seq Quality Control 7
Supplementary Figure 5| Global gene expression and chromatin accessibility patterns 8
Supplementary Figure 6| Number of differentially accessible regions and differentially expressed genes 9
Supplementary Figure 7| Wnt-pathway related transcription factor binding sites are enriched in upregulated WREs 10
Supplementary Figure 8| Stimulus-specific regulatory element-gene correlation 11
Supplementary Figure 9| Opposing gene regulation by WNT pathway inhibition 12
Supplementary Figure 10| ca/eQTL effect sizes compared to previous data 13
Supplementary Figure 11| eQTL-caQTL overlaps 14
Supplementary Figure 12| Effect size differences across conditions 15
Supplementary Figure 13| Response and Non−Response caPeaks enrichment within chromHMM Annotations 16
Supplementary Figure 14| Context-specific caPeak overlaps HAQER near HAR1A/B 17
Supplementary Figure 15| Colocalization of ANKRD44 r-eQTL, schizophrenia, and the volume of a hippocampal subfield (presubiculum body) 18
Supplementary Figure 16| Colocalization of an r-caQTL in DPYSL5 region and average thickness of the isthmus Cingulate 19
Supplementary Figure 17| Shared and context-specific QTL GWAS overlaps confirmed by eCAVIAR 20
Supplementary Figure 18| Stimulus-specific GWAS colocalization of FADS3 and Bipolar disorder 21
Supplementary Figure 19| Stimulus-specific ENO4 eQTL colocalizing with regional cortical surface area GWAS 22
Supplementary Figure 20| Optimizing control for known and unknown technical confounding in eQTL 23

Supplementary Tables **24**

Supplementary Table 1| Differentially accessible peaks 24
Supplementary Table 2| TF motif enrichment analysis 24
Supplementary Table 3| Differentially expressed genes 24
Supplementary Table 4| Pathway enrichment analysis 25
Supplementary Table 5| Summary of peak-gene correlation 26
Supplementary Table 6| Peak-gene pairs 26
Supplementary Table 7| Disease enrichment analysis results(DEG) 26
Supplementary Table 8| Summary of GWASs used in this study 27
Supplementary Table 9| Partitioned heritability analysis results 27
Supplementary Table 10| List of caQTL - caPeak 28
Supplementary Table 11| List of eQTL - eGene 29
Supplementary Table 12| caSNP TF motif disruption (in peak) 30

Supplementary Table 13| List of response-caQTLs 30
Supplementary Table 14| List of response-eQTLs 31
Supplementary Table 15| TFBS motif enrichment in response-caPeaks 32
Supplementary Table 16| caQTL Regional Overlaps 32
Supplementary Table 17| caQTL-eQTL colocalizations 33
Supplementary Table 18| GWAS colocalization results (caQTL) 33
Supplementary Table 19| GWAS colocalization results (eQTL) 34
Supplemental Information References 35

Supplementary Figures

Supplementary Figure 1| Multidimensional scaling analysis of genotype data

Multi-dimensional scaling (MDS) plot shows the first two components of genetic similarity for all HapMap populations and donors in this study, allowing inference of genetic ancestry. Multiple ancestries of donors are present in this population.

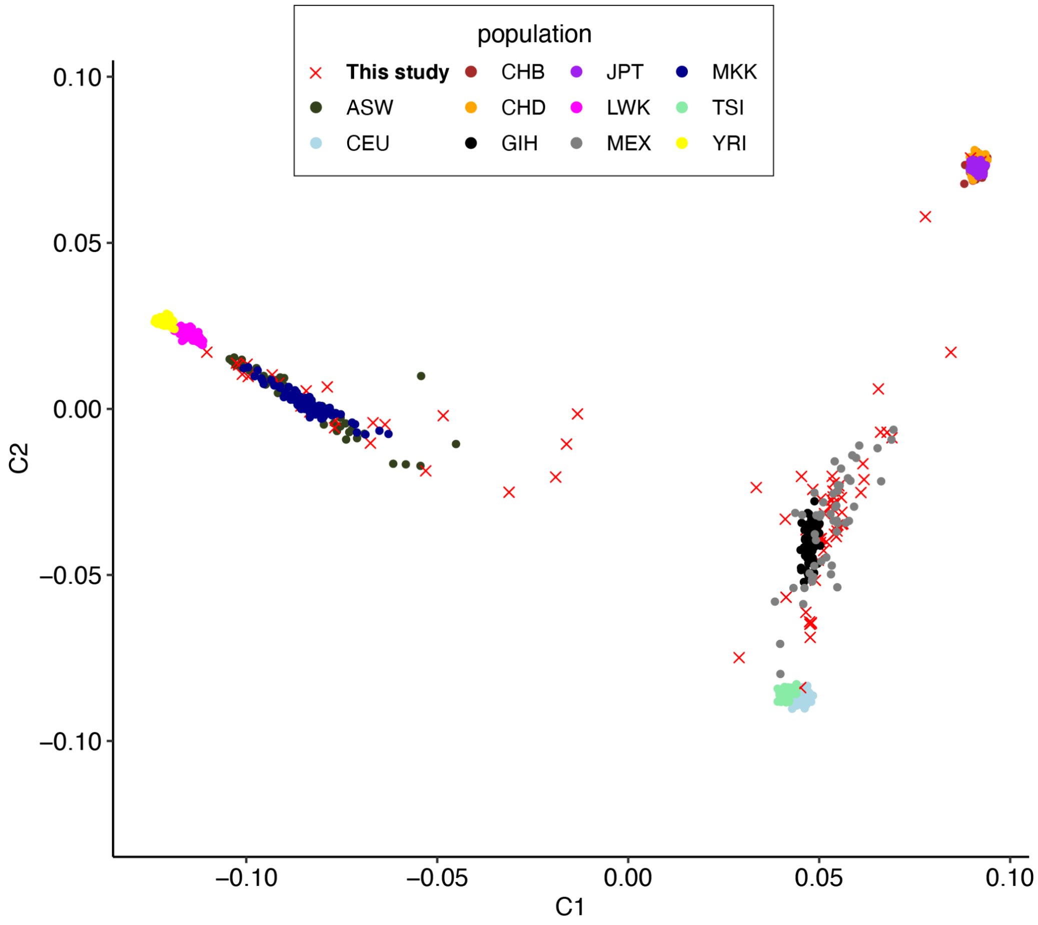

Supplementary Figure 2| Effects of CHIR and WNT3A on proliferation and Wnt activity

Cellular assays show that WNT3A and CHIR stimulation increase canonical Wnt pathway signaling and hNPC proliferation. Diagram of luciferase reporter assay measuring β-catenin mediated Wnt pathway activation by CHIR or WNT3A (**A**). Effects of 48h CHIR (**B**) or WNT3A (**C**) exposure on Wnt pathway activation, reported as the log of luciferase luminescence (activated by Wnt stimulation) normalized by renilla luminescence (from a constitutively active reporter transgene). Representative flow cytometry scatter plots from proliferation assays depict newly synthesized DNA from a 2h hr EdU pulse vs total DNA content following 48h vehicle (left) or 2.5uM CHIR (right) exposure (**D**). Percentage of cells in S-phase (%EdU+) exposed to vehicle or increasing doses of CHIR for 48h as measured by flow cytometry in a subset of cell-lines (**E**), and in all cell-lines used for ATAC-seq and RNA-seq in this study (**F**). Representative immunocytochemistry images (100x magnification) from proliferation assay of hNPCs following 48h vehicle (left) and 5nM WNT3A (right) exposure (**G**). Green (GFP) labels cells in S-phase during the EdU pulse, and blue (DAPI) stains all nuclei. Percentage of cells in S-phase (%EdU+) exposed to vehicle or increasing doses of WNT3A for 48h as measured by flow cytometry in a subset of cell-lines (**H**). CHIR and WNT3A concentrations (boxed) that maximize Wnt activation and proliferative responses were used in this study for ca/eQTL mapping.

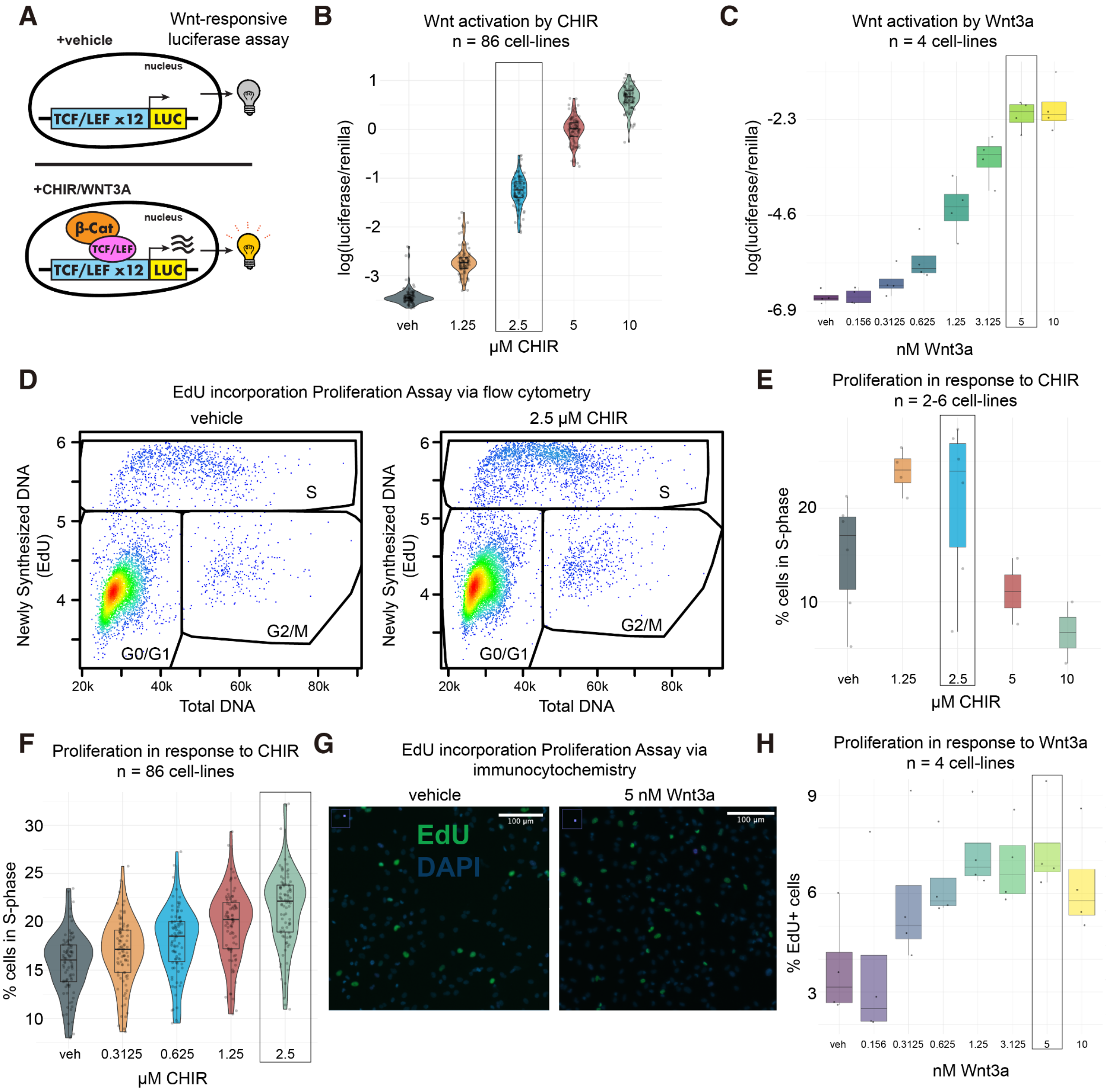
A
+vehicle
Wnt-responsive luciferase assay
nucleus
TCF/LEF x12
LUC
+CHIR/WNT3A
β-Cat
TCF/LEF
B
Wnt activation by CHIR
n = 86 cell-lines
log(luciferase/renilla)
veh
1.25
2.5
5
10
μM CHIR
C
Wnt activation by Wnt3a
n = 4 cell-lines
-2.3
-4.6
-6.9
0.156
0.3125
0.625
3.125
nM Wnt3a
D
EdU incorporation Proliferation Assay via flow cytometry
vehicle
2.5 μM CHIR
Newly Synthesized DNA (EdU)
S
G2/M
G0/G1
Total DNA
E
Proliferation in response to CHIR
n = 2-6 cell-lines
% cells in S-phase
F
Proliferation in response to CHIR
n = 86 cell-lines
G
EdU incorporation Proliferation Assay via immunocytochemistry
5 nM Wnt3a
EdU
DAPI
100 μm
H
Proliferation in response to Wnt3a
n = 4 cell-lines
% EdU+ cells

Supplementary Figure 3| Technical reproducibility of RNA-seq and ATAC-seq

Pairwise correlations normalized sequencing counts from technical replicates across open chromatin peaks measured by ATAC-seq (**A**) or genes measured by RNA-seq (**B**). Higher correlations for within donor vs across donor pairs indicates robust reproducibility of measurements for a given donor and condition. Violin plots represent the distribution of Pearson correlation coefficients calculated between a pair of genotypically distinct donors ("across donor") or between two technical replicates of the same donor cultured at different times ("within donor"). Top and bottom horizontal lines within violin plots represent the interquartile range of the data, and the middle bar represents the median. P-values report significant differences between the across vs. within donor correlations following Fisher's Z transformation and evaluated with the Welch two sample t-test. The total number of cell-lines and pairwise correlations (n) for each experimental condition is reported along the x-axis.

A

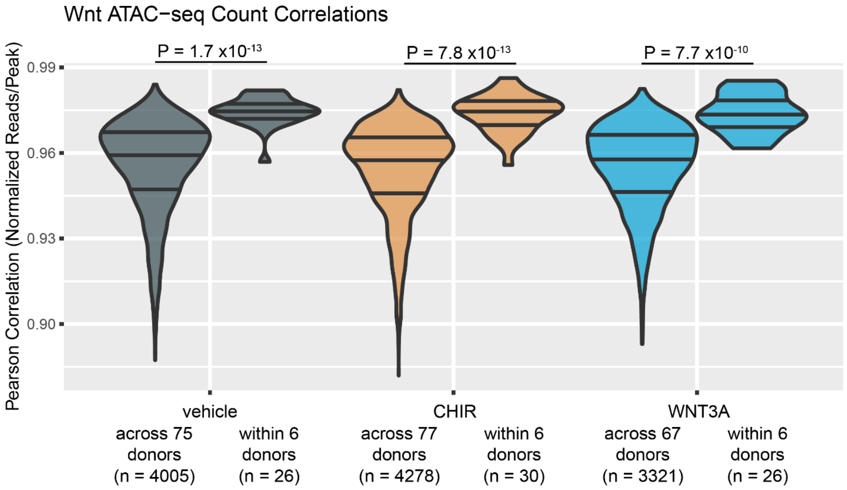

B

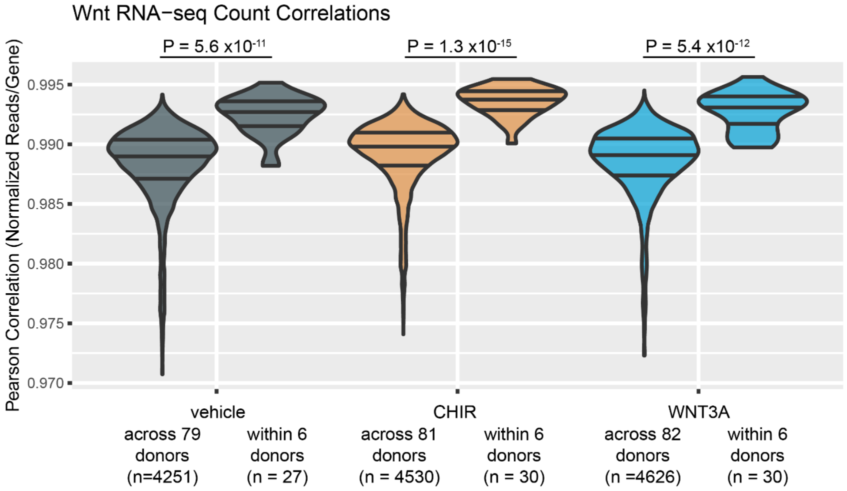

Supplementary Figure 4| ATAC-seq Quality Control

Total ATAC-seq reads (**A**), unique non mitochondrial reads (**B**), transcription start site (TSS) enrichment (**C**), total read duplication rate (**D**), mitochondrial read duplication rate (**E**), and fraction of reads within chromatin accessibility peaks (FRiP) (**F**) across stimulus conditions. Mean insert size distributions in base pairs across all samples colored by stimulus condition (**G**) exhibit a nucleosomal phasing pattern; right: insert size distribution plotted on log- scale. Mean TSS enrichments (> 7) and FRiP scores (>0.3) exceed the "ideal" metrics for ATAC-seq libraries defined by the ENCODE project[1]. Together, these measurements validate the quality of ATAC-seq samples used in this study.

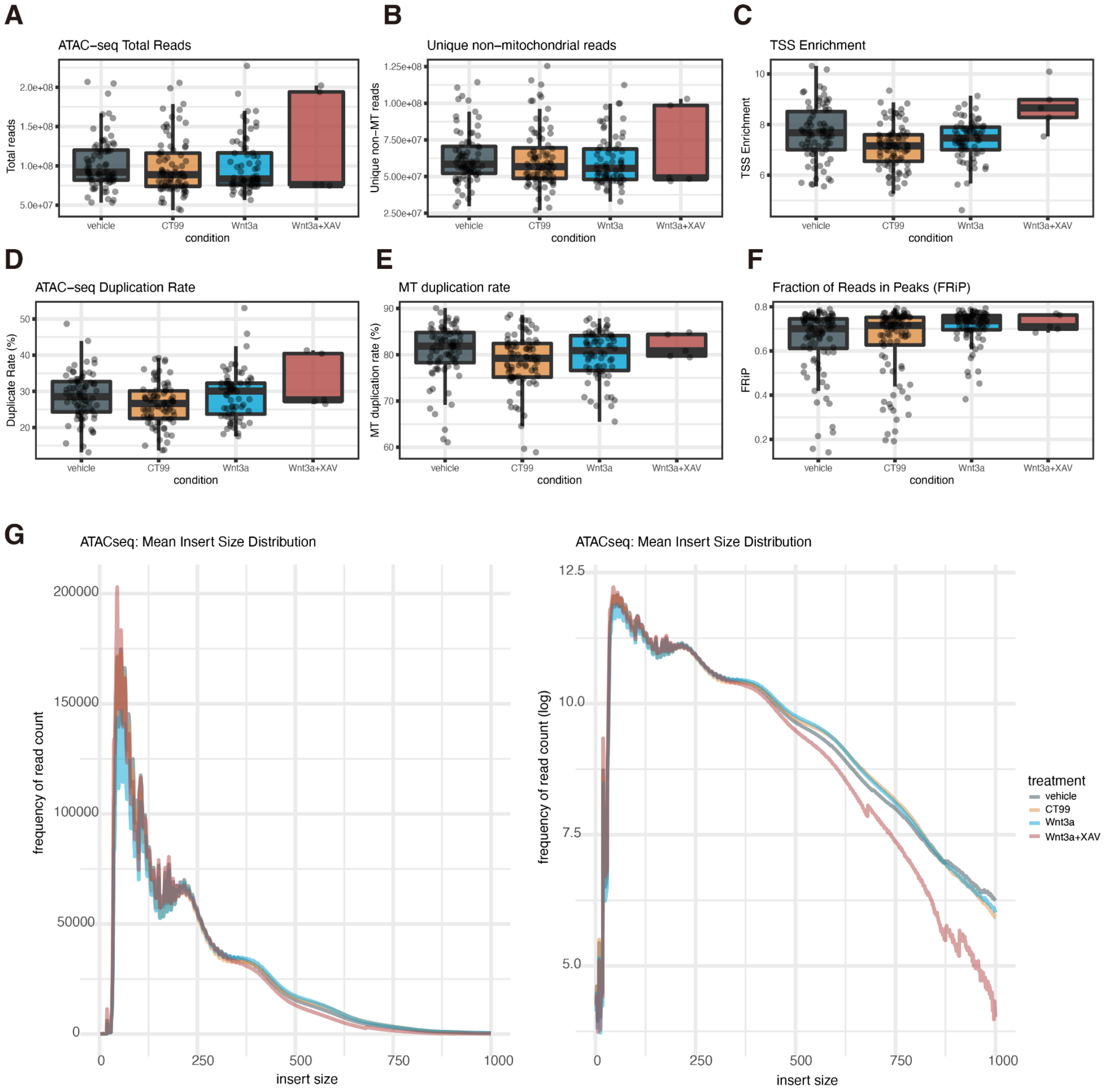

Supplementary Figure 5| Global gene expression and chromatin accessibility patterns

Principal component analysis (PCA) of ATAC-seq (**A, C**) and RNA-seq (**B, D**) data after batch correction for technical variables included in differential accessibility or expression models, respectively. Samples are labeled by condition (**A**, **B**), or sex (**C**, **D**). Variance in global gene expression and chromatin accessibility profiles across the first two PCs is driven by stimulation condition, but not by sex. Correlation matrix of ATAC-seq (**E**) or RNA-seq (**F**) principal components 1-10 with technical (RIN) and biological variables (donor and sex). We performed linear regression to remove effects of PCs 1-10 and the residualized sequencing count data was used as QTL model input to account for measured and unmeasured confounding.

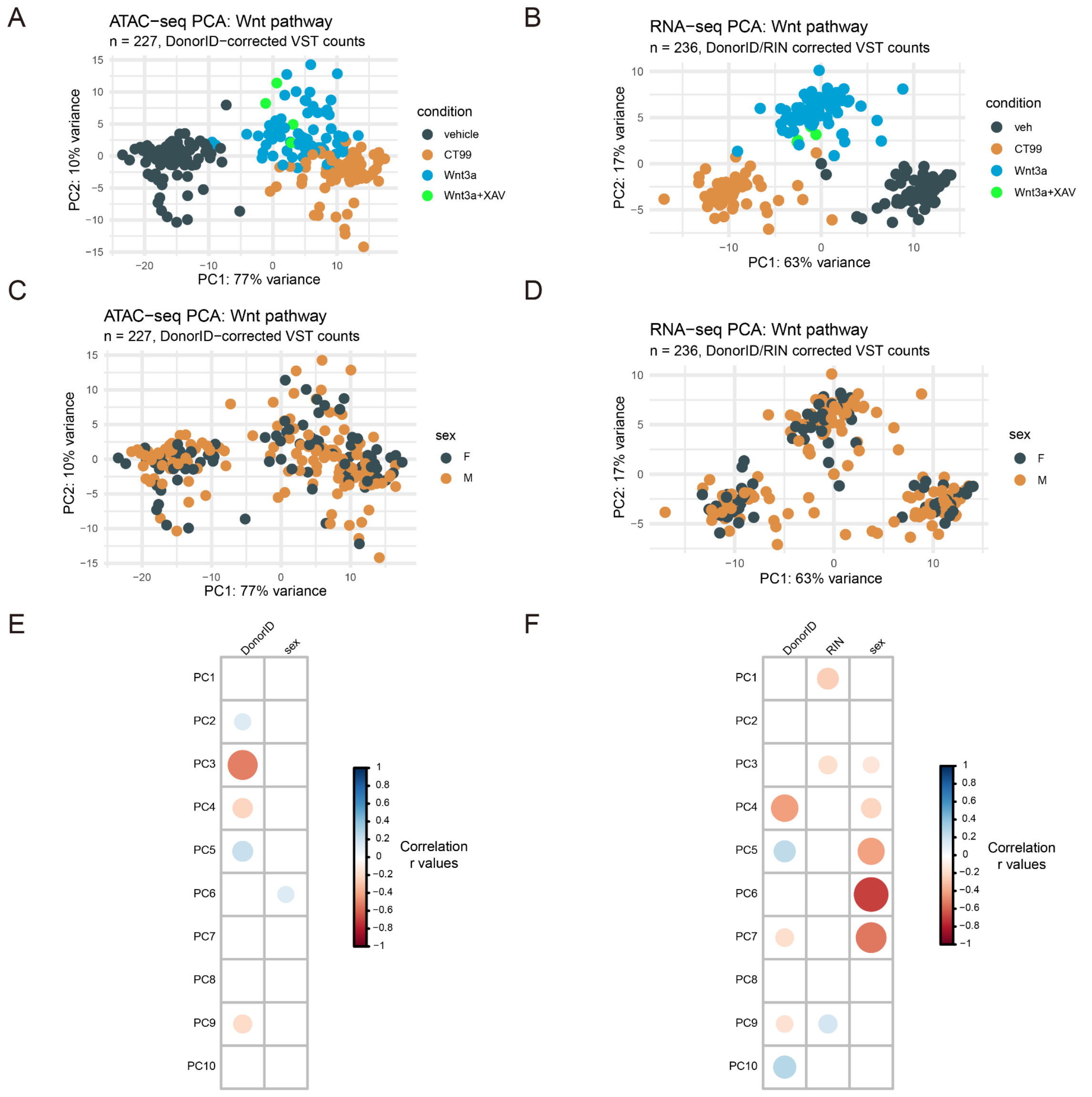

Supplementary Figure 6| Number of differentially accessible regions and differentially expressed genes

Barplots showing the number of differentially accessible regions (WREs) (**A**) and differentially expressed genes (DEGs) (**B**). The reference indicates referenced condition (either vehicle or WNT3A). Sample size indicates paired samples between conditions. Percentage of increased (shrunken log2FC > 0.5 in dark blue, shrunken log2FC > 0 in blue) or decreased (shrunken log2FC < -0.5 in red, shrunken log2FC < 0 in pink) accessibility or expression are shown for each category. Nonsignificant peaks and genes (FDR-adjusted P >=0.1) are colored gray. Greater changes were observed in both chromatin accessibility and gene expression for CHIR, a potent Wnt activator, as compared to WNT3A, an endogenous Wnt ligand. Simultaneous WNT3A activation and inhibition via XAV as compared to WNT3A activation yielded few differentially expressed genes and chromatin elements, likely due to the low sample size. We also note that 93,853 peaks and 9,890 genes were filtered out (adjusted *P* value were set to *NA*) by DESeq2[2,3] as those have low mean read counts.

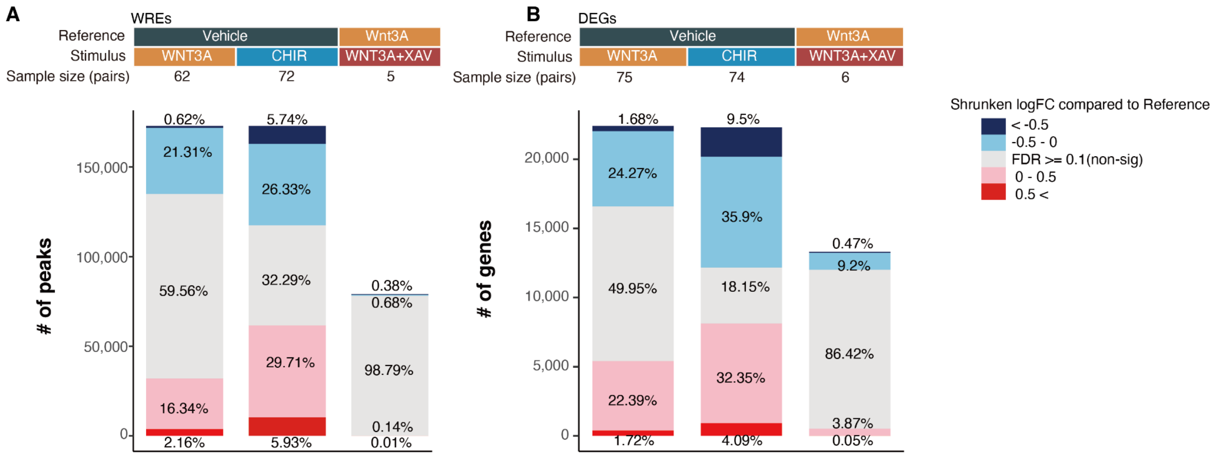

Supplementary Figure 7| Wnt-pathway related transcription factor binding sites are enriched in upregulated WREs

The canonical Wnt-pathway downstream effectors, ß-catenin, LEF1, and TCF7L2 binding sites previously identified by ChIP-seq experiments in HEK cells[1,4] are more often overlapped with WREs opening due to WNT3A (**A**) or CHIR (**B**) than closing WREs. The figures show the percentage of overlaps with the indicated TF binding sites in either the 2,000 most upregulated peaks or the 2,000 most downregulated peaks based on shrunken logFC. Statistical significance was estimated using Fisher's exact test on the number of overlaps.

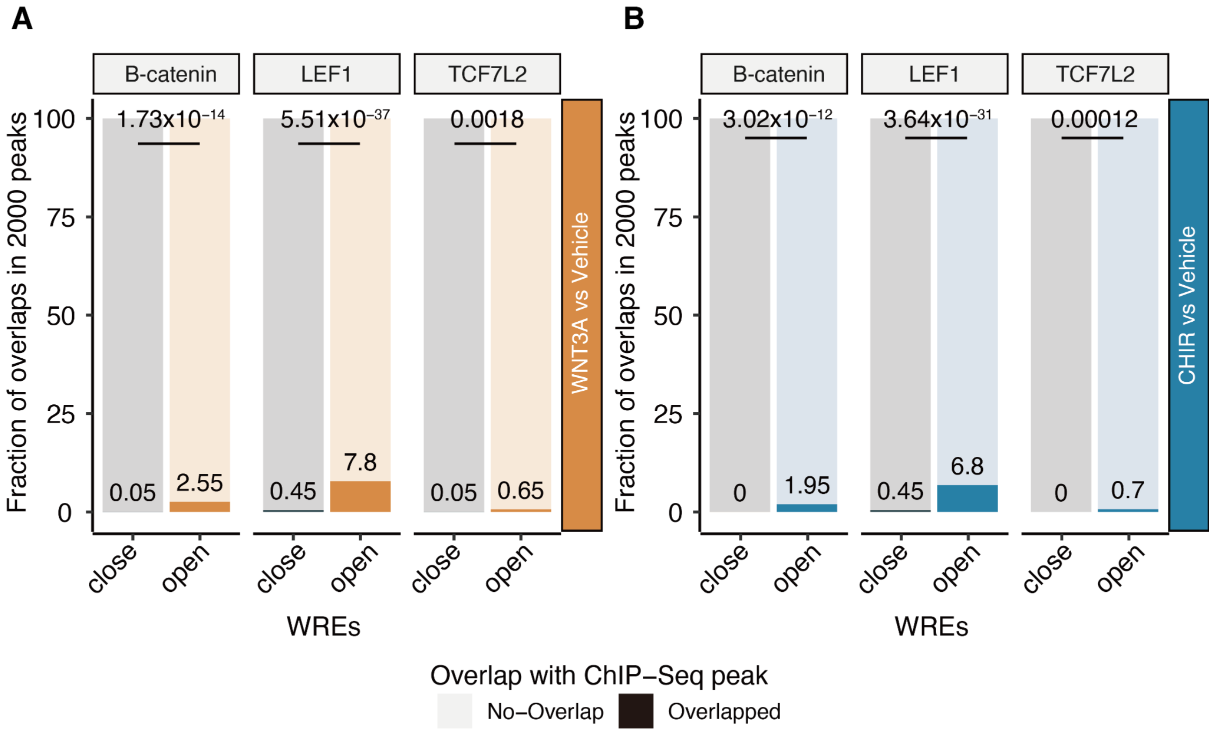

Supplementary Figure 8| Stimulus-specific regulatory element-gene correlation

We identified stimulus-specific regulatory elements which regulate gene expression by correlating expression with chromatin accessibility. The number in the plot indicates significant and positively correlated peak-gene pairs. In total, 12,643 peak-gene pairs are detected only under the stimulus condition (highlighted in red), demonstrating new stimulus-specific regulatory elements.

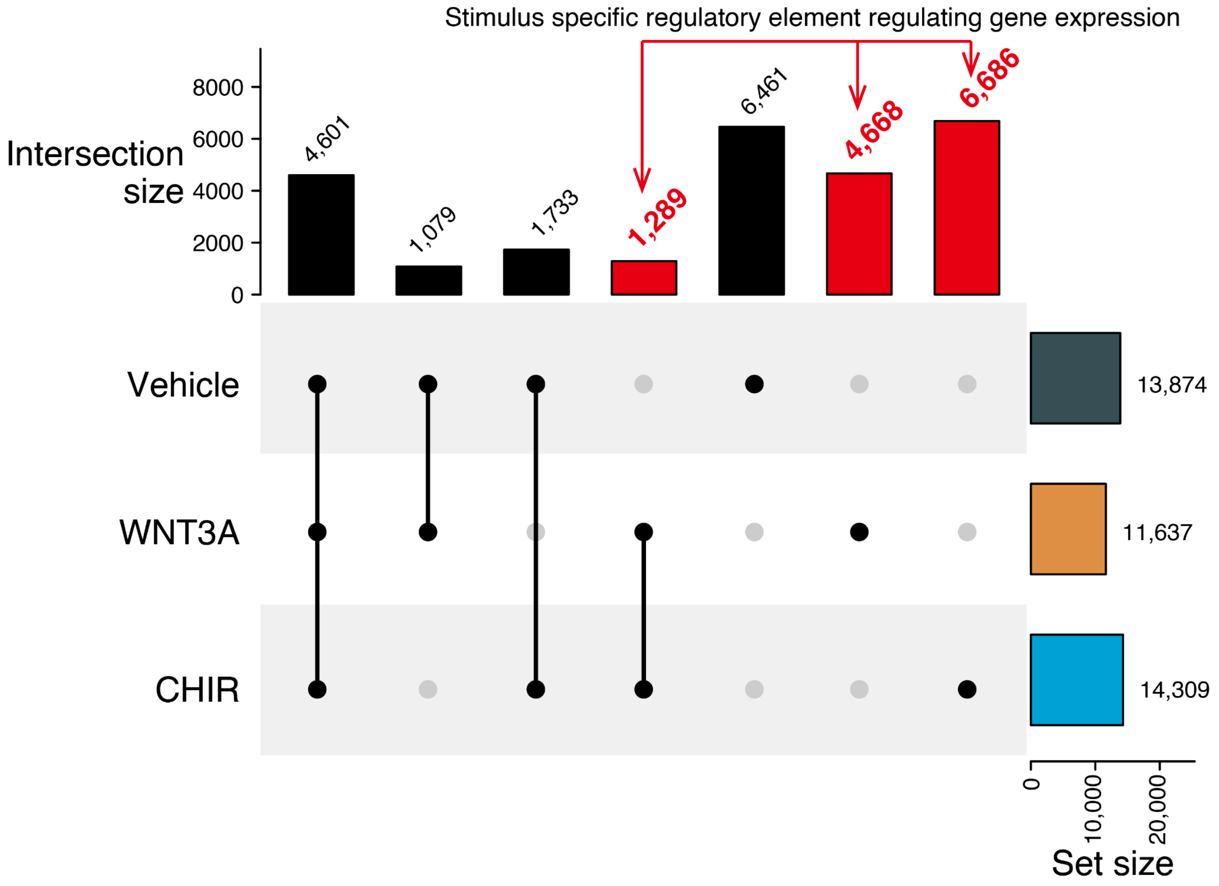

Supplementary Figure 9| Opposing gene regulation by WNT pathway inhibition

(**A**) Volcano plot showing differential expressed genes between simultaneous excitation and inhibition of WNT3A (WNT3A+XAV) as compared to WNT3A stimulus alone. Note that LEF1, a downstream effector of the Wnt pathways, has decreased expression under inhibition of the Wnt pathway. (**B**) Among 1413 genes that are differentially expressed at BH-adj P < 0.1 in both comparisons (WNT3A+XAV vs WNT3A (6 pairs) and WNT3A vs Vehicle (75 pairs)), 70.8% (1001 genes), opposing changes in gene expression were observed. This result is consistent with most gene expression changes being caused by the stimulus of the Wnt pathway.

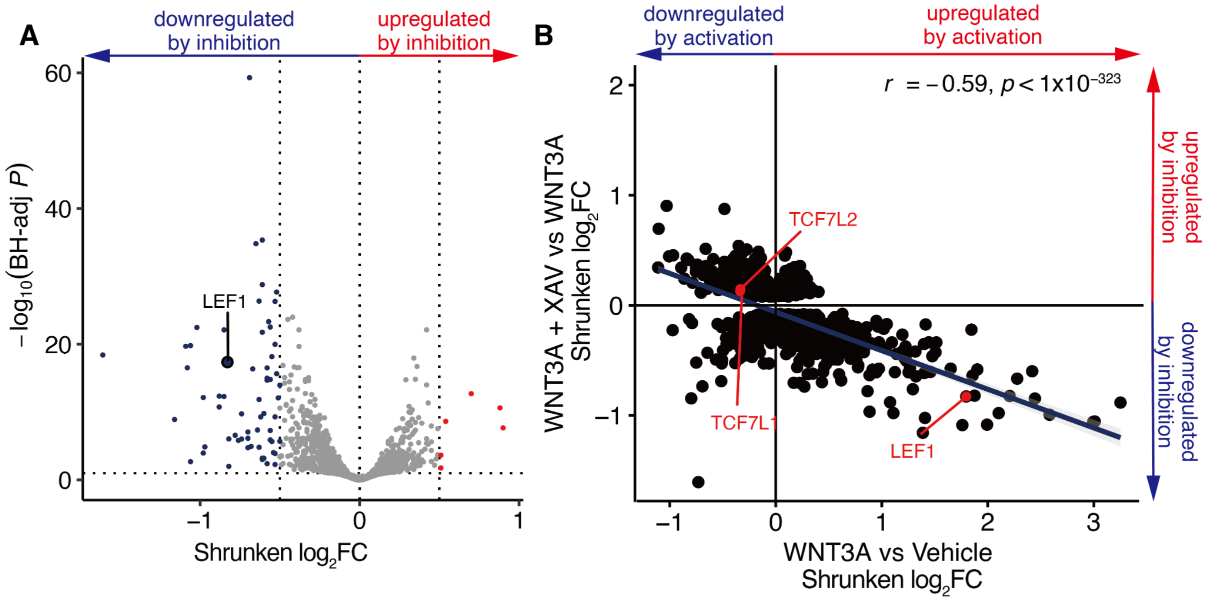

Supplementary Figure 10| ca/eQTL effect sizes compared to previous data

We compared the effect size of ca-QTL (**A**) and eQTL (**B**) in vehicle condition to previous studies using the same hNPC cell lines with slightly larger sample size[5,6]. Strongly correlated effect sizes for unstimulated ca/eQTLs were found between the two datasets. In part **A**, the current study beta values are derived using RASQUAL as a scaled allelic ratio ranging from -0.5 to 0.5, which is not on the same scale as the beta values from Liang et al., which are calculated using a linear mixed effect model. One SNP showed opposite effect directionality across the caQTL datasets between the current and previous datasets (rs2076179). We suspect this was due to an error in allele assignment for that SNP in the previous analysis caused by sampling differences incorrectly changing the labeled minor allele for a SNP with allele frequency close to 0.50. We corrected this error for the one SNP prior to plotting.

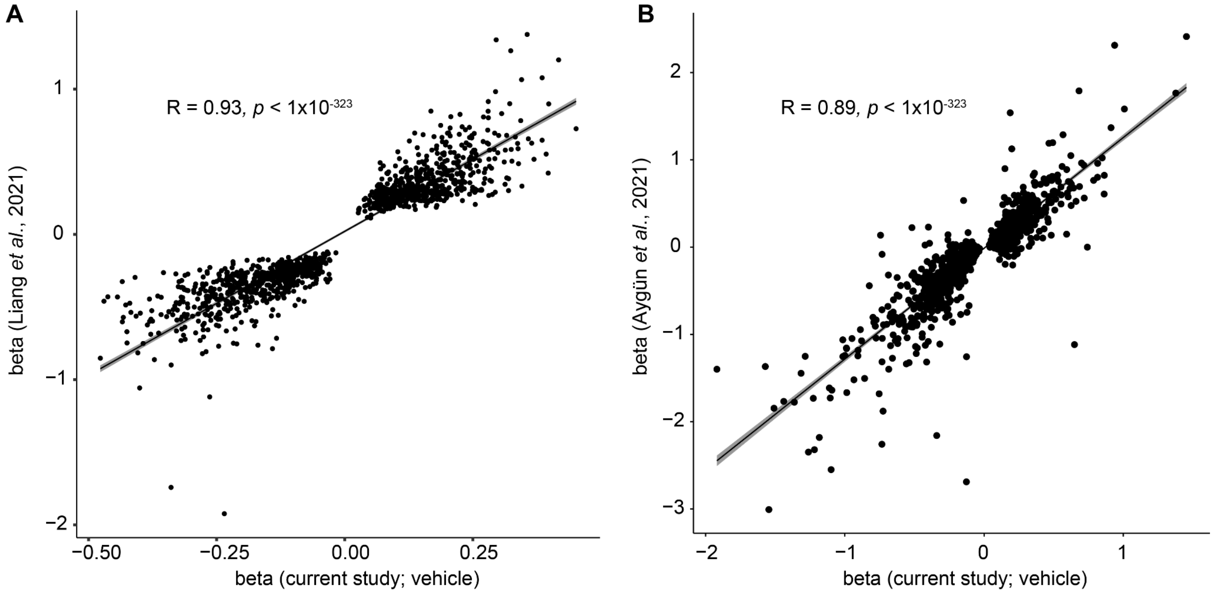

Supplementary Figure 11| eQTL-caQTL overlaps

Overlaps between caQTLs and eQTLs were called where a caSNP was within 1Mb of an eGene and at least 1 significant SNP in both datasets were in LD $r^2 \geq 0.8$. The percentage of eQTLs (**A**) and caQTLs (**B**) overlapped within vehicle and WNT stimulus conditions are shown. The effect size of caQTL-eQTL sites which shared the same SNP position were compared within vehicle (**C**) and WNT stimulus conditions (WNT3A and CHIR shown in **D** and **E** respectively). SNPs selected to influence chromatin accessibility had relatively little overlap with those influencing gene expression likely because genetic variation affecting chromatin accessibility often does not lead to changes in gene expression (about <2% of caQTLs had a shared eQTL based on LD overlap (r^2 >= 0.8). However, SNPs selected to influence gene expression more often also influence chromatin accessibility in that ~27% of eQTLs have a shared caQTL within condition, a comparable number observed in our previous study (34.9% caQTL-eQTLs overlap[5]).

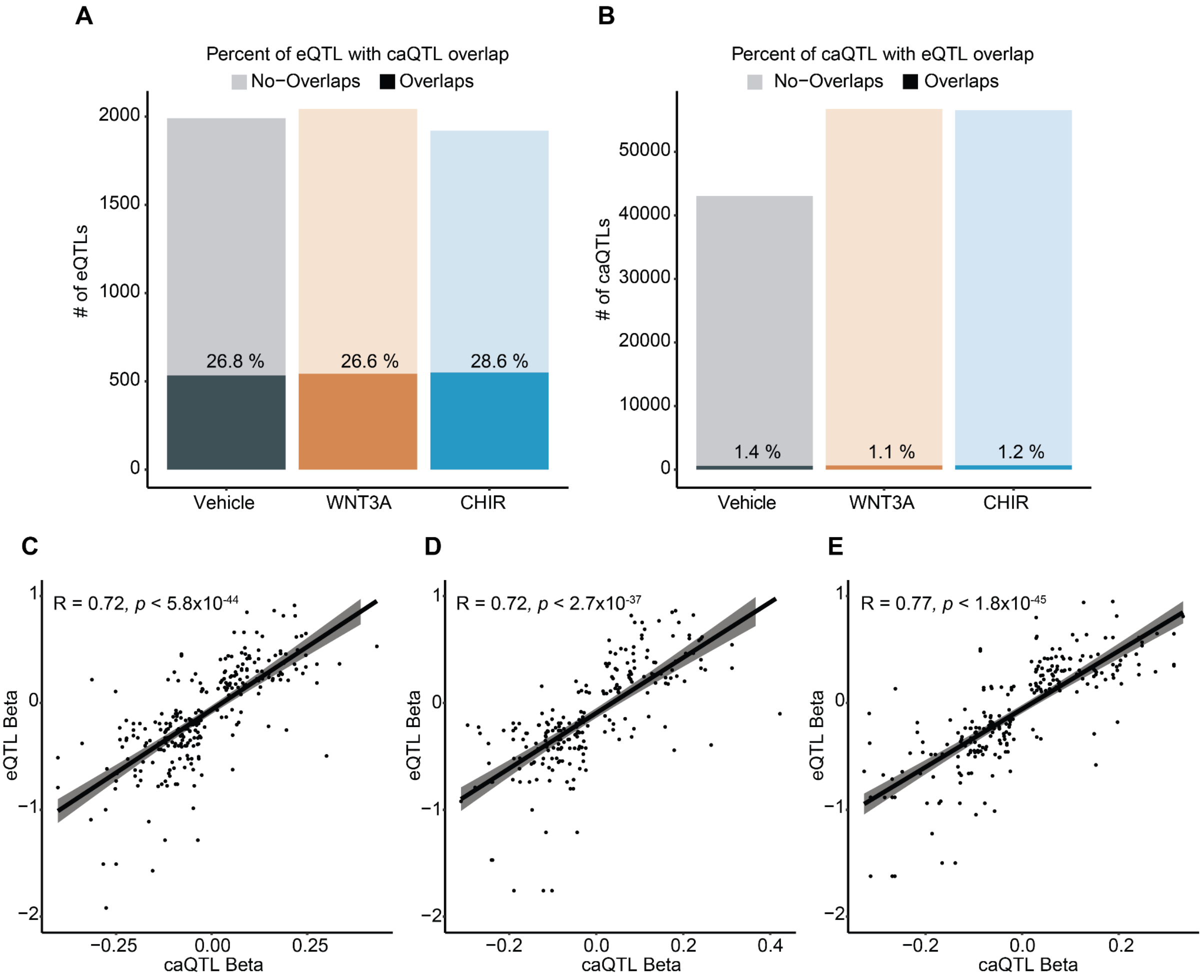

Supplementary Figure 12| Effect size differences across conditions

We compared effect sizes of index caQTLs (**A**-**B**) / eQTLs (**C**-**D**) in stimulated conditions versus vehicle conditions (WNT3A or CHIR in (**A,C**) and (**B,D**), respectively). Dots in red indicate significant interaction effects were observed (r-QTLs). Error bars are standard errors of beta. We observed lower correlation in r-QTLs as compared to non-r-QTLs. We note that some SNPs are not tested for a baseline model due to low expression thus could not be plotted because no beta value was calculated.

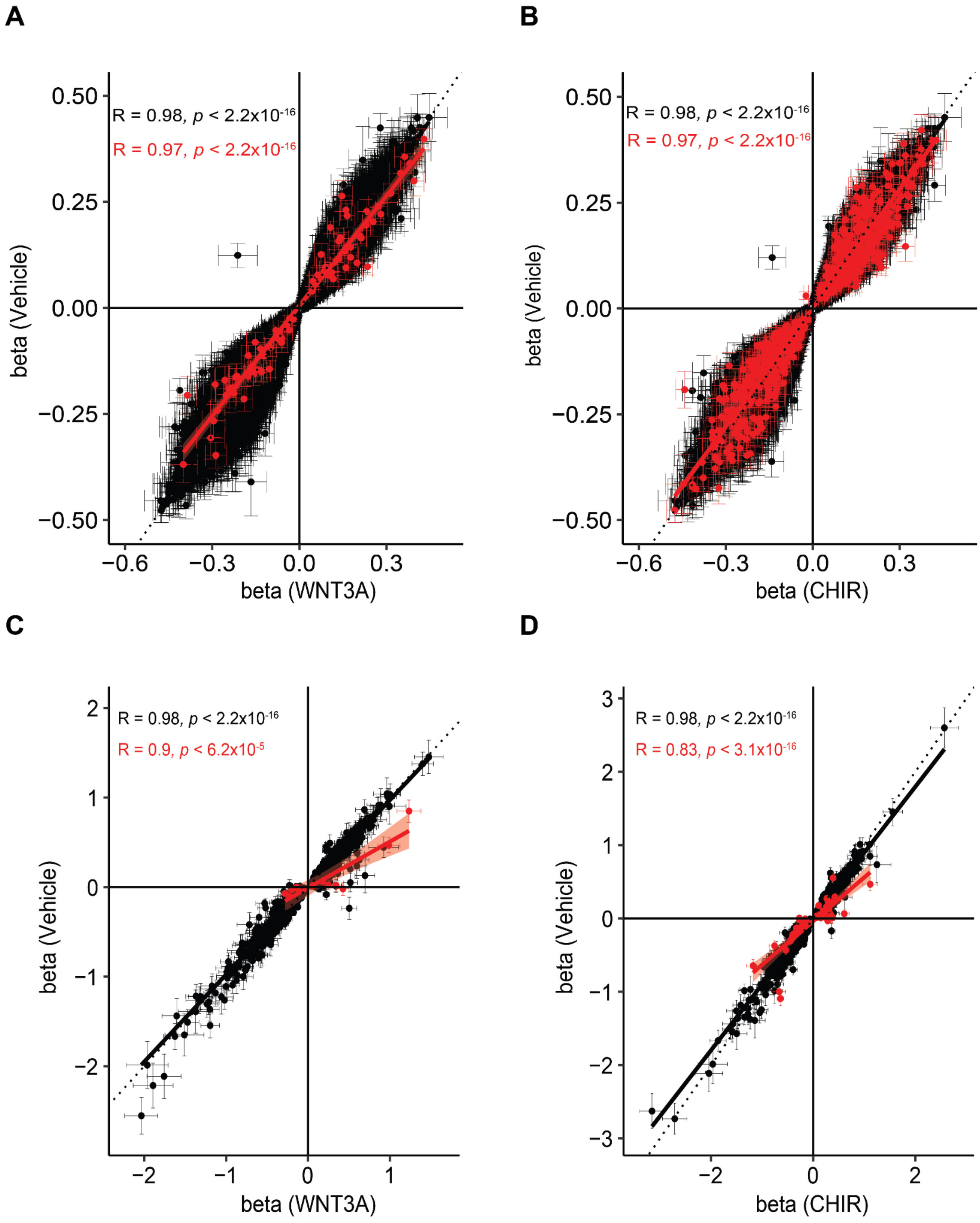

Supplementary Figure 13| Response and Non−Response caPeaks enrichment within chromHMM Annotations

Enrichment of response and non-response caPeaks within chromHMM states defined within fetal brain male (E081)[7]. *, **, *** indicate enrichments of overlaps relative to the entire genome, evaluated with a binomial test, similar to the GREAT test[8], with *P*-values < 0.05, .01 and .001, respectively. We observed a significant enrichment of both response and non-response caPeaks within promoters, enhancers, and bivalent regions. TssA, TssFlnk, and Enh states showed a significant difference in overlap counts between response and non-response caPeaks (P = 3.9×10^{-5}; 2.8×10^{-4}; 7.4×10^{-7}, respectively), as evaluated with Fisher's exact test. There was significantly less enrichment of response caPeaks in active TSS and enhancers as compared to non-response caPeaks, perhaps because these response caPeaks flag novel condition specific enhancers not annotated in post-mortem fetal brain tissue.

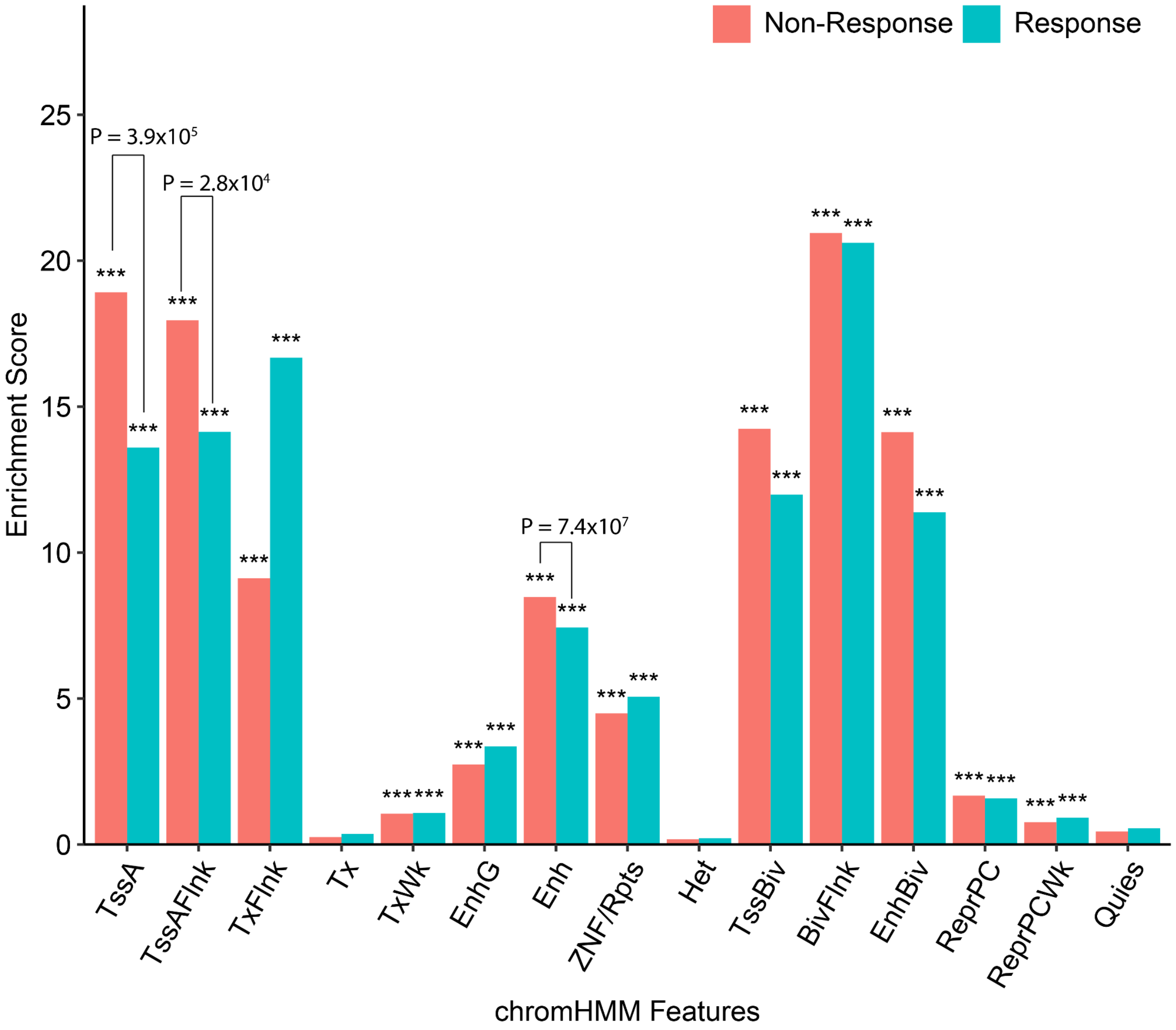

Supplementary Figure 14| Context-specific caPeak overlaps HAQER near HAR1A/B

(**A**) Allelic effects of rs112675456 on chromatin accessibility (chr20:63101761-63102670), reveal a caQTL significant in CHIR stimulated condition but not vehicle. (**B**) Regional association plots at rs112675456, the index SNP for a caPeak-HAQER overlap near the transcription start sites of HAR1A/B. From top to bottom: Genomic coordinates, gene models, caQTL *P* values for vehicle and CHIR-stimulated conditions, ATAC-seq coverage showing differential chromatin accessibility, and HAQER.

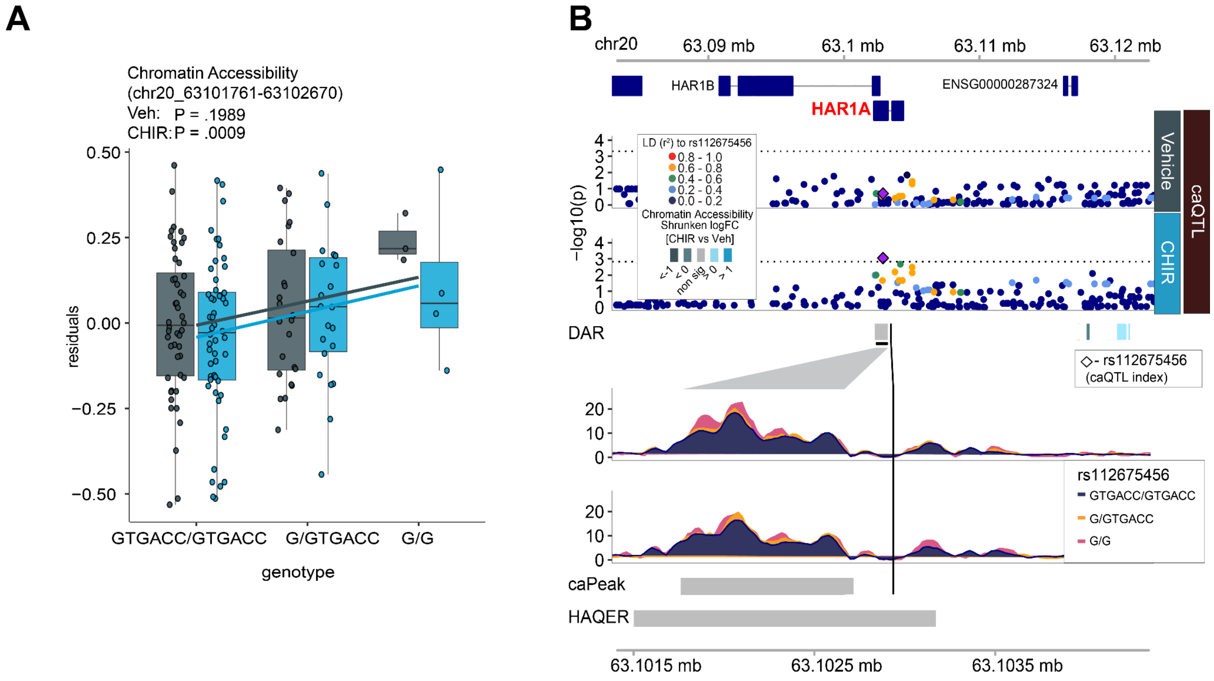

Supplementary Figure 15| Colocalization of ANKRD44 r-eQTL, schizophrenia, and the volume of a hippocampal subfield (presubiculum body)

(**A**) Boxplot showing increased ANKRD44 gene expression by rs979020-T and the SNP a significant interaction effect. (**B**) P-P plot from schizophrenia GWAS vs eQTL colored by r^2 in this study population to rs979020, providing evidence for a colocalization. (**C**) Conditional analysis of schizophrenia GWAS was performed using GCTA-cojo tool with LD from the UKBB reference panel (White British). GWAS *P* value (upper panel) and post-conditional analysis *P* value (bottom panel) are shown. An absence of GWAS signal after conditioning on the r-eQTL index provides evidence for colocalization. (**D**, **E**) Similar to (**B**, **C**) but using GWAS for volume of a hippocampal subfield (presubiculum body; left hemisphere).

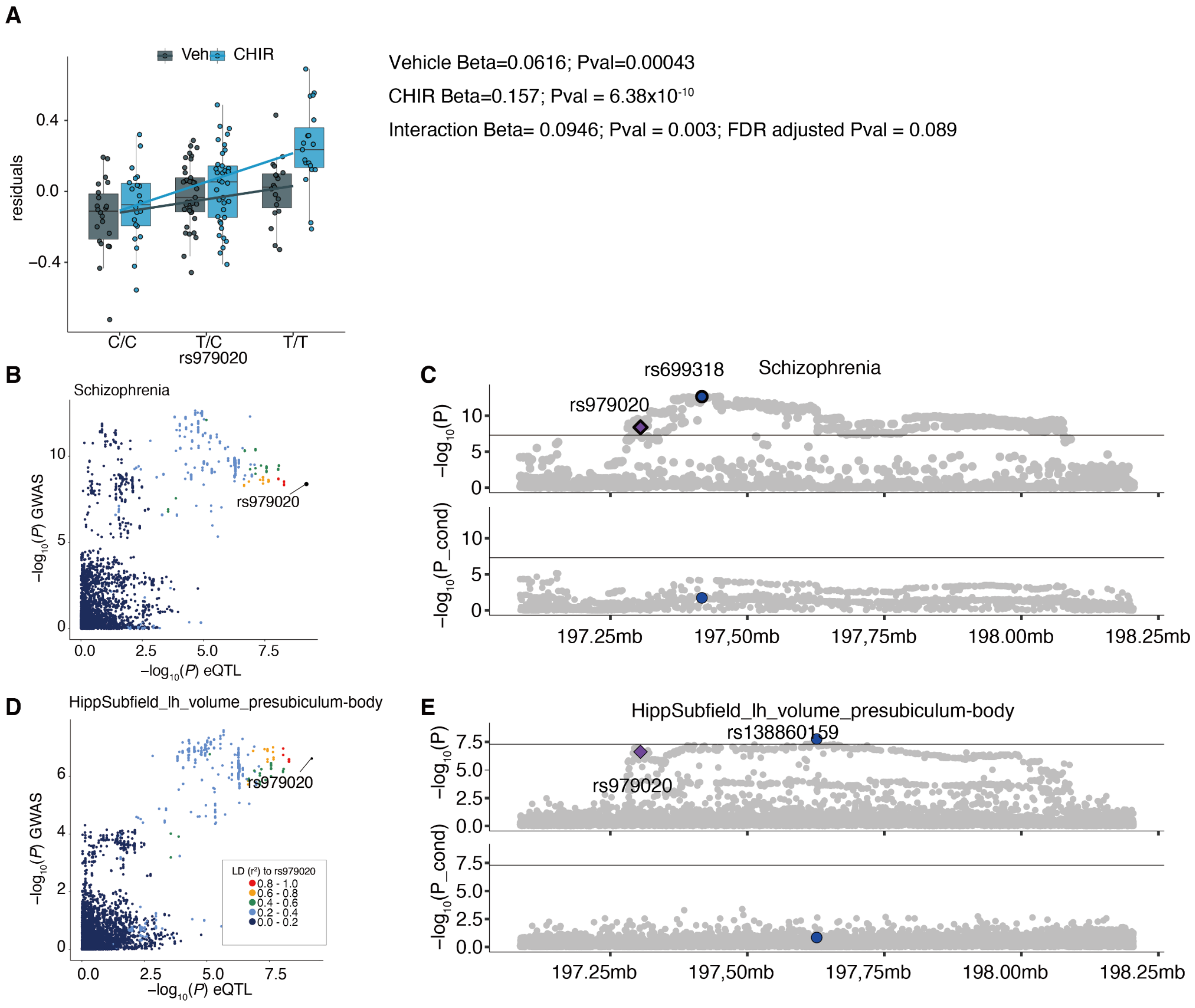

Supplementary Figure 16| Colocalization of an r-caQTL in DPYSL5 region and average thickness of the isthmus Cingulate

(**A**) We detected an r-caQTL at rs1992311 (FDR-adjusted *P* value = 0.04). We present boxplots showing differential genetic effects on chromatin accessibility in stimulated versus vehicle conditions in a linked SNP within the peak that disrupts a motif (rs4665363-G). (**B**) P-P plot for isthmus Cingulate GWAS[9] vs caQTL colored by r^2 in our population to rs1992311, providing evidence for colocalization. (**C**) Boxplot showing decreased *DPYSL5* expression by rs4665363-G. (**D**) Conditional analysis on GWAS for average thickness of Isthmus Cingulate was performed using the UKBB reference panel (White British). GWAS *P* value (upper panel) and post-conditional analysis *P* value (bottom panel) are shown. GCTA-cojo identified collinearity between rs4665363 and rs3820823 (r^2>0.9) thus both SNPs are not shown in the bottom panel. A decrease in GWAS signal after conditioning on the r-caQTL provides evidence for colocalization. (**E**) Logo plot predicting disruption of the Pou5f1::Sox2 motif by rs4665363-G. (**F**) Coverage plots of the peak showing the location of rs4665363 and a TCF/LEF motif present in the peak.

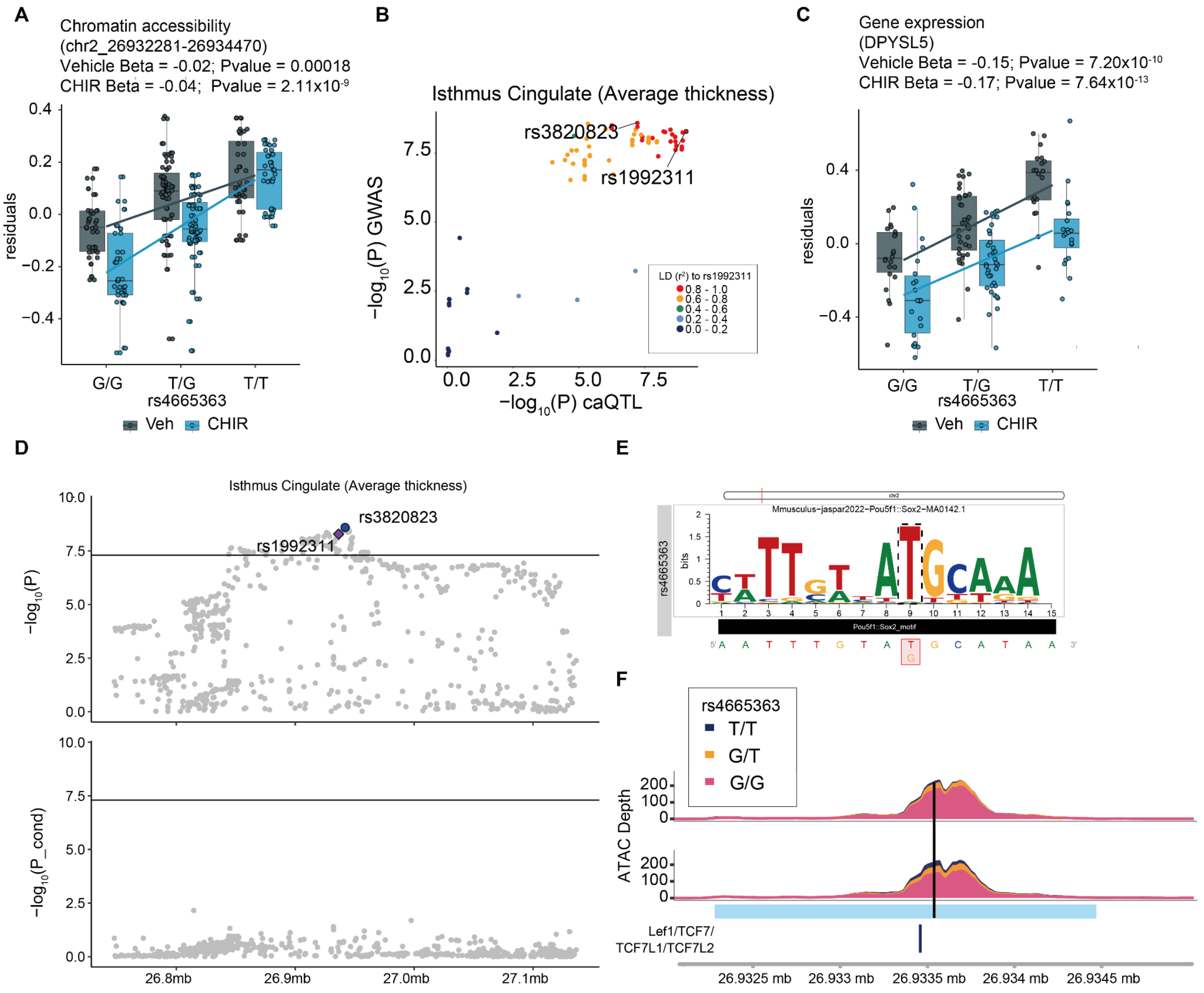

Supplementary Figure 17| Shared and context-specific QTL GWAS overlaps confirmed by eCAVIAR

Colocalization of eQTLs/caQTLs and brain-related GWAS phenotypes tested by eCAVIAR. (**A**) A cumulative number of colocalized eGenes/caPeaks after colocalization analysis by eCAVIAR and (**B**) shared/distinct colocalization in each condition. The use of stimulated conditions increased the number of brain-trait associated genes by 75.6% and peaks by 77.0%.

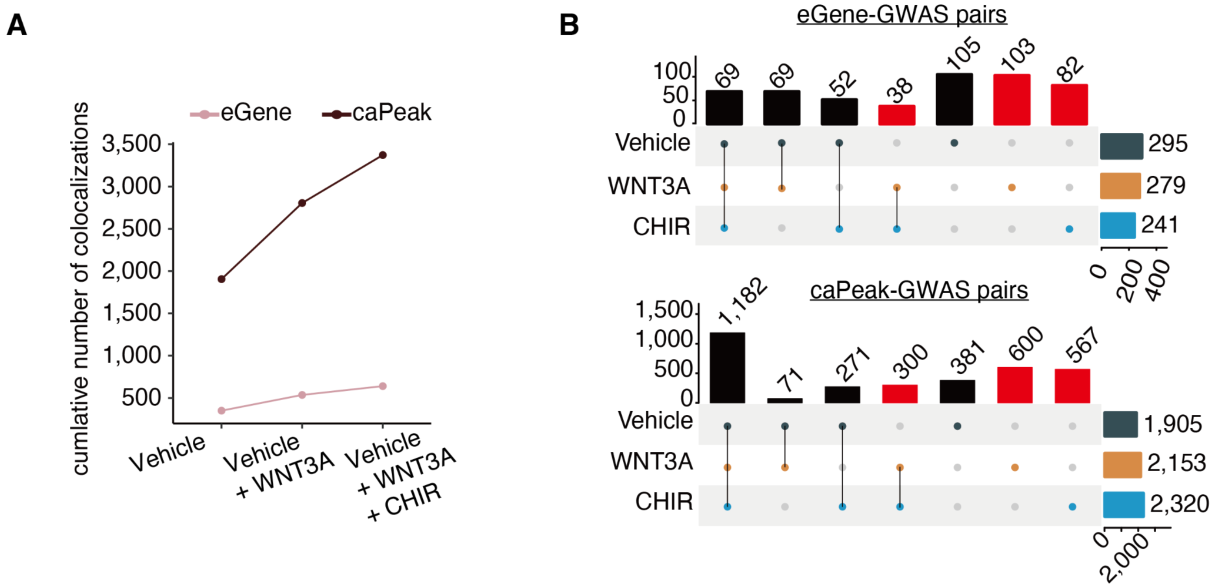

Supplementary Figure 18| Stimulus-specific GWAS colocalization of *FADS3* and Bipolar disorder

(**A**) GWAS and QTL locus plots in the region of *FADS3*. GWAS index SNP rs174592-A (protective allele) of bipolar disorder (BP) is in high LD with index caQTL (rs99780, r^2 = 0.86 in EUR, 0.85 in this study population) for RE (chr11:61850491-61851800) and *FAD3* index eQTL (rs174583, r^2 = 0.89 in EUR, r^2 = 0.78 in this study population). Rs174592-A is located within the peak and predicted to disrupt a STAT1::STAT2 transcription factor binding site motif. (**B**) Rs174592-A decreases chromatin accessibility of this WRE (Beta = -0.10; P = 6.81 x 10^{-13}, left), and expression of *FADS3* (Beta = -0.13 P = 2.95 x 10^{-7}, right) in CHIR condition. (**C**) Differential chromatin accessibility and *FADS3* gene expression between CHIR and Vehicle. (**D**) VST normalized expression counts of STAT1 and STAT2 are shown. Shrunken LFC and FDR-adjusted P values were estimated for 78 pairs. In the boxplots, chromatin accessibility or gene expression are colored by condition (gray: vehicle, blue:CHIR). (**E**) Schematic of FADS3 regulation through STAT1::STAT2 binding. Our data suggests characterization of the colocalized putative BP risk SNP (rs174592) as a functional variant regulating *FADS3* expression through differences in STAT1::STAT2 binding, which is inhibited during Wnt stimulation.

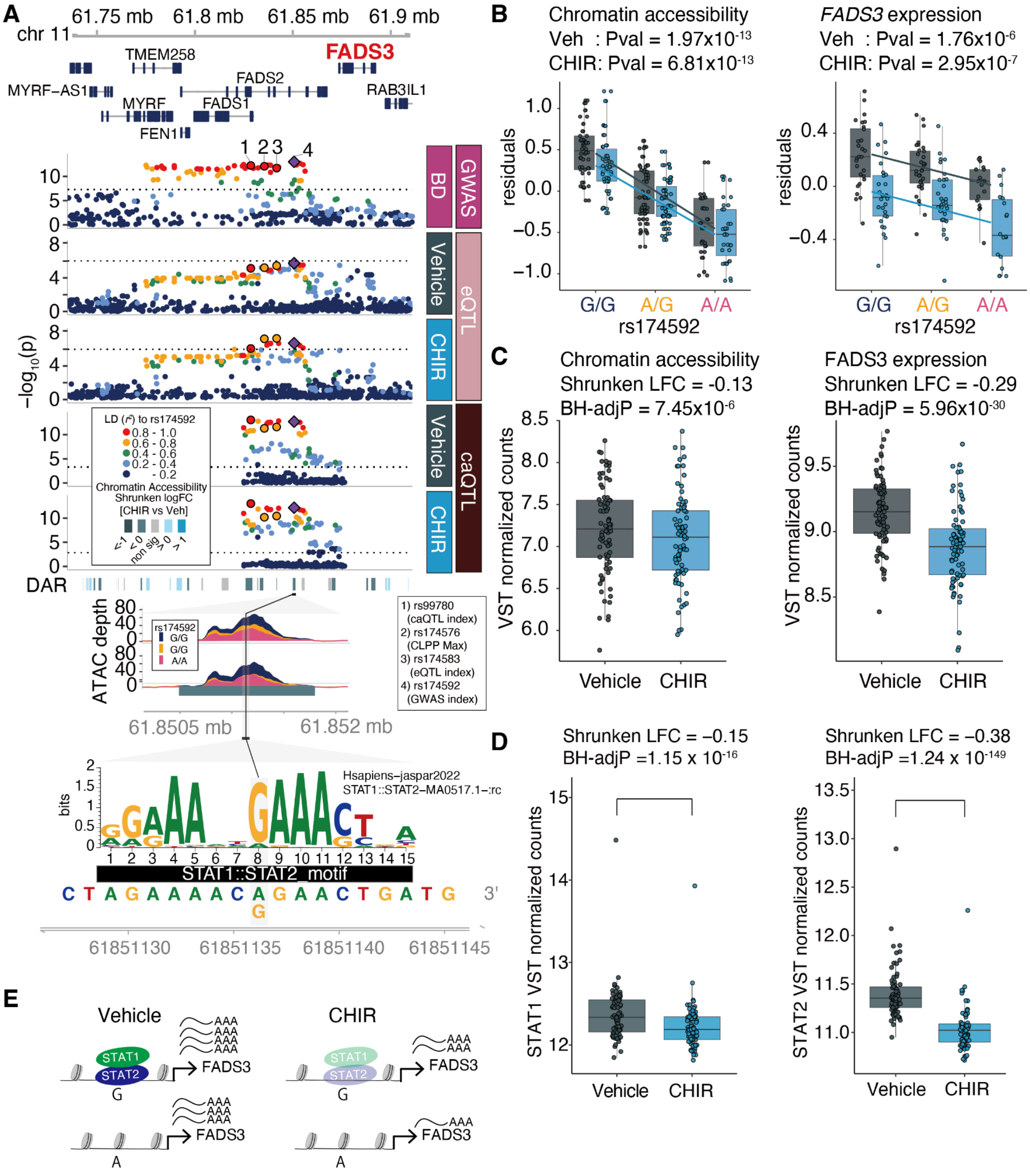

Supplementary Figure 19| Stimulus-specific *ENO4* eQTL colocalizing with regional cortical surface area GWAS

(**A**) Chromatin accessibility (chr10:116973711-116975130; left) and *ENO4* gene expression regulated by rs11197861. eQTL of *ENO4* was detected only in the stimulated condition (**A**, **B**). The eQTL is colocalized with several brain-related traits including surface area of Insula (**B**, **C**). (**B**) *P* values from Insula surface area GWAS, eQTL, caQTL for vehicle, CHIR condition are shown respectively. ETV2:FOXI1 motif is disrupted by the rs11197861-T located in the peak, which may result in decreased chromatin accessibility and *ENO4* gene expression. (**C**) Z scores from regional cortical surface area[9]. In addition to Insula, surface area of precentral and precuneus are also associated with this SNP (indicated by asterisks; $p < 5 \times 10^{-8}$).

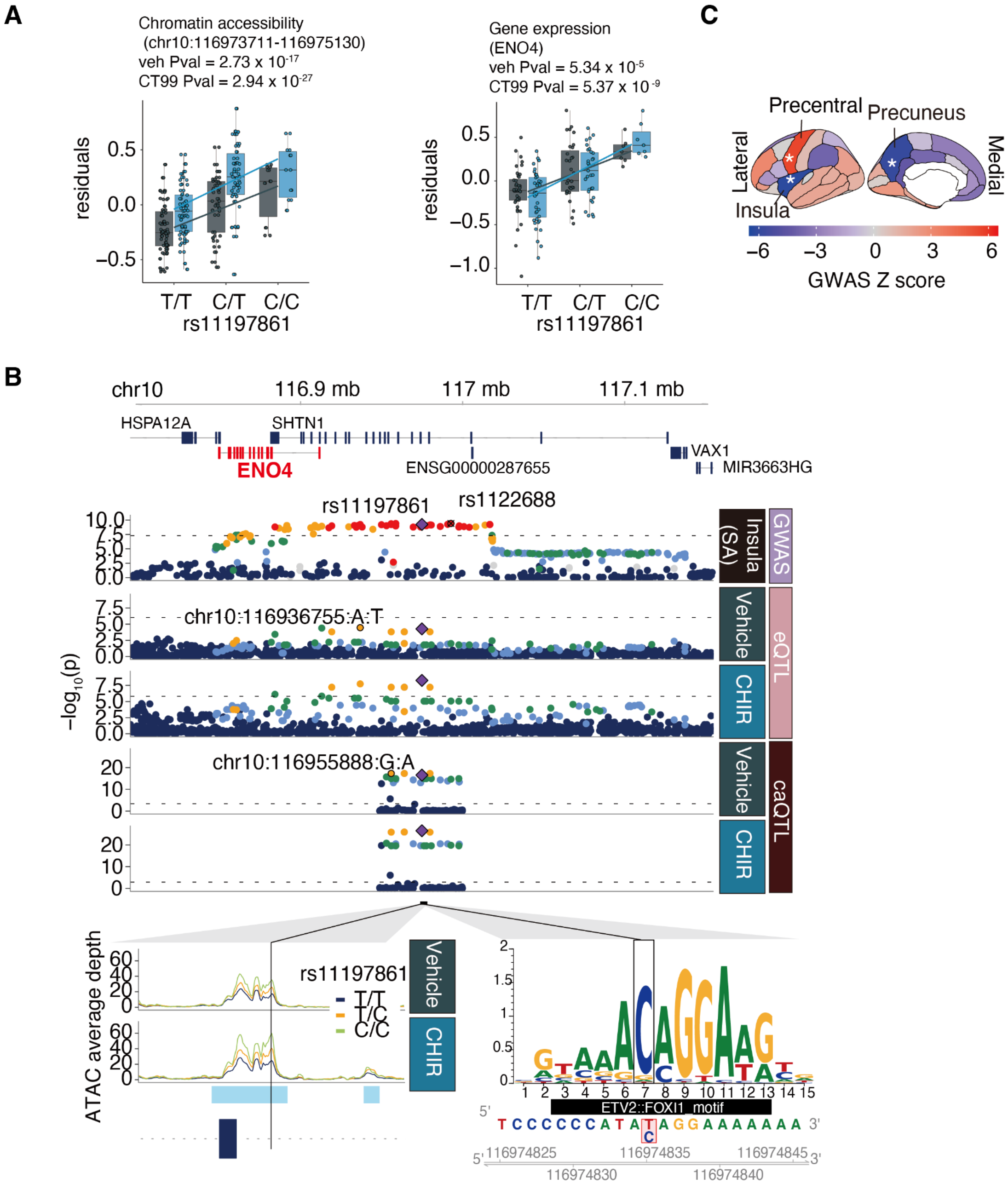

Supplementary Figure 20| Optimizing control for known and unknown technical confounding in eQTL

We tested different numbers of PC variables to determine the number of PCs to include for correcting expression values prior to running limix_qtl. (**A**) The plot shows the number of discovered eGenes with respect to the number of PCs at FDR-adjusted P < 0.1. and the number of overlapping found eGenes across models (**B-D**). To identify independent eQTLs, we repeated eQTL mapping by including the index eSNP in the association model until no SNP passed the genome-wide threshold (FDR-adjusted P < 0.1). In table (**E**), we show the number of SNPs discovered in each round of conditional associations.

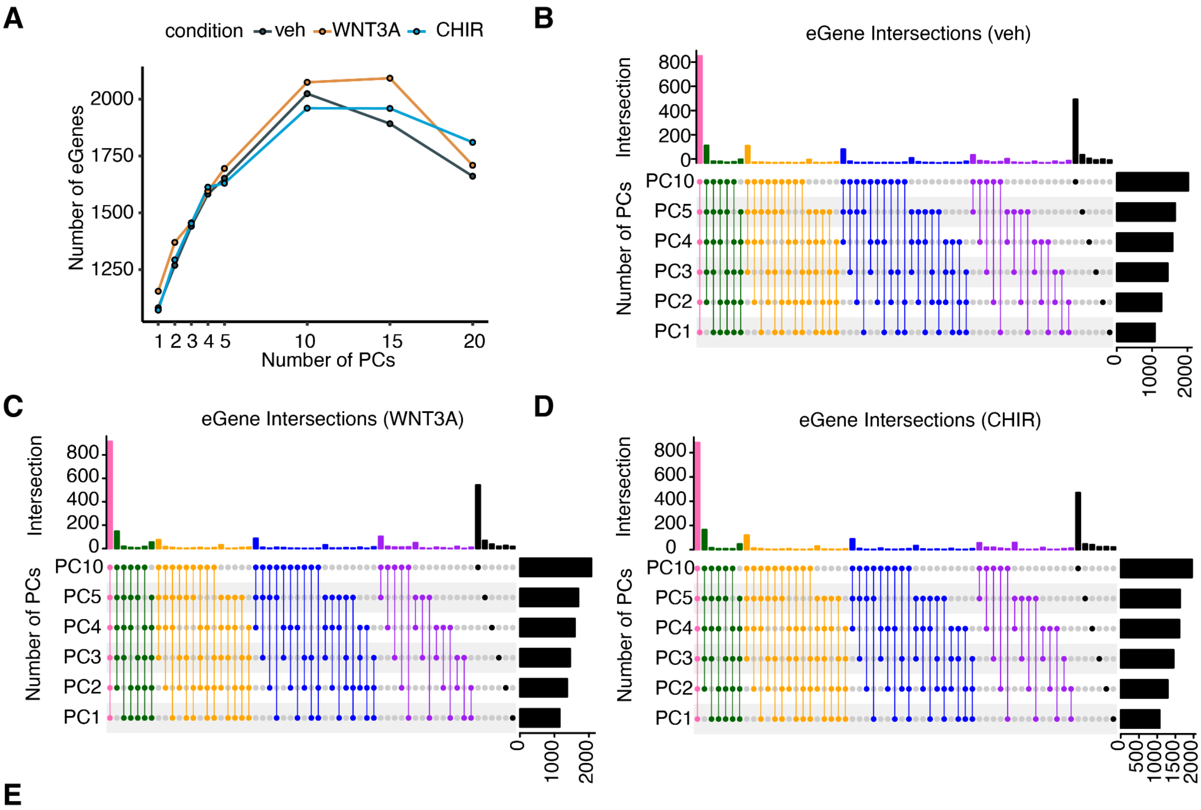

E

# of eSNPs per Gene		
**Round**	**# eGenes**	**condition**
1	2024	Vehicle
2	65	Vehicle
1	2074	Wnt3A
2	72	Wnt3A
3	1	Wnt3A
1	1960	CHIR
2	65	CHIR
3	1	CHIR

Supplementary Tables

S1 - S4, S6-S19 are provided in Excel files.

Supplementary Table 1| Differentially accessible peaks

A. WNT3A vs Vehicle; B. CHIR vs Vehicle; C. WNT3A+XAV vs WNT3A

Column Name	Description
Chromosome	chromosome
start	peak start position (hg38)
end	peak start position (hg38)
baseMean	average of the normalized count values
log2FoldChange	shrunken log2Fold Change
lfsSE	standard error of Shrunken log2Fold Change
pvalue	p value
padj	BH-adjusted Pvalue

Supplementary Table 2| TF motif enrichment analysis

A. WNT3A vs Vehicle; B. CHIR vs Vehicle

Column Name	Description
TFname	TF name
TFID	TF ID
estimate	coefficient from logistic regression
estimate_err	coefficient standard error from logistic regression
z	z score = estimate/estimate_err
pval	pvalue from logistic regression
padj	BH-adjsted Pvalue

Supplementary Table 3| Differentially expressed genes

A. WNT3A vs Vehicle; B. CHIR vs Vehicle; C. WNT3A+XAV vs WNT3A

Column Name	Description

gene_id	Ensembl ID
gene_name	HGNC gene name
gene_type	protein_coding/lncRNA
chromosome	chromosome
start	collapsed gene start position
end	collapsed gene end position
strand	strand
baseMean	average of the normalized count values
log2FoldChange	shrunken log2Fold Change
lfcSE	standard error of Shrunken log2Fold Change
pvalue	p value
padj	BH-adjusted Pvalue

Supplementary Table 4| Pathway enrichment analysis

A. WNT3A vs Vehicle; B. CHIR vs Vehicle
Details can be found on the gprofiler website (https://biit.cs.ut.ee/gprofiler/page/apis)

**Column Name**	**Description**
query	DEG direction (up-regulated/down-regulated/all)
source	KEGG / REAC(REACTOME)
term_id	pathway ID
term_name	pathway name
p_value	Hypergeometric p-value after correction for multiple testing (BH)
query_size	number of genes in the query
intersection_size	number of genes in the query annotated to the corresponding term
term_size	number of genes annotated to the term
effective_domain_size	total number of genes in the background that is used for Hypergeometric test
GeneRatio	interaction_size/query_size

Supplementary Table 5| Summary of peak-gene correlation

Condition	Pearson's Correlation Coefficient			% of positively correlated pairs
	max r	mean(abs.r)	min r	
Vehicle	0.95	0.50	-0.78	80.4%
WNT3A	0.95	0.53	-0.83	83.4%
CHIR	0.97	0.50	-0.75	83.6%

Supplementary Table 6| Peak-gene pairs

A. WNT3A vs Vehicle; B. CHIR vs Vehicle

Column Name	Description
chromosome	chromosome position of peak
start	start position of peak
end	end position of peak
gene_id	Ensembl ID
gene_name	HGNC gene name
distance	distance from TSS to center of peak
coefficient	coefficient in a linear model
fdr	BH-adjusted P value
(interaction coefficient)	Interaction coefficient in a linear model (WNT3A/CHIR vs Vehicle)
(fdr_interact)	BH-adjusted P value for interaction test (WNT3A/CHIR vs Vehicle)

Supplementary Table 7| Disease enrichment analysis results(DEG)

Details can be found in DisGeNet (https://www.disgenet.org/home/).

Column Name	Description

status	DEG category (all/up/down/nonsig)
cond	condition (WNT3A/CHIR/nonsig)
ID	phenotype ID
Description	phenotype description
source	"CURATED"
BgRatio	number of genes associated to the disease and number of background gene in database
pvalue	Fisher's exact test P value
FDR	BH-adjusted P value
disease_class	MESH tree branch class
disease_class_name	MESH tree branch name
disease_semantic_type	UMLS® semantic types
shared_symbol	shared gene symbol associated to the disease

Supplementary Table 8| Summary of GWASs used in this study

**Column Name**	**Description**
GWAS Name	GWAS phenotype
Ref	Reference

Supplementary Table 9| Partitioned heritability analysis results

**Column Name**	**Description**
Annotation type	Tissue / Cell / WNT stimulation
Data set	WREs used for LDSC
GWAS phenotype	GWAS phenotype
Coefficient	coefficient from LDSC model
Coefficient stderr	standard error of Coefficient

Coefficient P	One-sided Pvalue from Coefficient Z score
BH-adjusted P	BH-adjusted Pvalue

Supplementary Table 10| List of caQTL - caPeak

**Column Name**	**Description**
Feature	peak ID
rs.ID	SNP ID
Chromosome	chromosome position of peak/snp
SNP.Position	SNP position
Ref.Allele	reference allele
Alt.Alele	alternative allele (the effect allele)
Allele.Frequency	allele frequency of reference allele
HWE.Chi_Square.Statistic	Hardy-Weinberg chi-square statistic
Imputation.Quality..IA.	imputation quality score
Log_10.Benjamini.Hochberg.Q.value	$\log_{10}$Benjamini-Hochberg q-value
Chi.square.statistic..2.x.log.Likelihood.ratio.	chi square statistic (2 x log-likelihood ratio)
Effect.Size	cis-regulatory effect parameter (pi)
Sequencing.mapping.error.rate..Delta.	sequencing/mapping error rate (Delta)
Reference.allele.mapping.bias..Phi.	reference allele mapping bias (Phi)
Overdispersion	overdispersion estimate
SNP.ID.within.the.region	ID of snp within tested region
No..of.feature.SNPs	total number of snps tested within the feature
No..of.tested.SNPs	total number of snps tested within cis window
No..of.iterations.for.null.hypothesis	number of iterations for null hypothesis
No..of.iterations.for.alternative.	number of iterations for alternative hypothesis

hypothesis	
Random.location.of.ties	location of snp used as lead if tie occurs
Log.likelihood.of.the.null.hypothesis	log-likelihood of the null hypothesis
Convergence.status	job outputs status (0=success)
Squared.correlation.between.prior.and.posterior.genotypes..fSNPs.	squared correlation between prior and posterior genotypes (fSNPs; feature snps)
Squared.correlation.between.prior.and.posterior.genotypes..rSNP.	squared correlation between prior and posterior genotypes (rSNP; single cis-regulatory snp)
PValue	p value
Distance_from_PeakCenter	distance of tested snp from the center of the peak

Supplementary Table 11| List of eQTL - eGene

**Column Name**	**Description**
gene_id	Ensembl ID
gene_chr	chromosome position of gene
gene_start	start position of gene
gene_end	end position of gene
gene_name	HGNC gene name
feature_strand	strand (+/-)
snp_id	snp ID
snp_chr	chromosome position of SNP
snp_pos	SNP position
Assessed Allele	Assessed (also called effect) allele
maf	minor allele frequency
beta	beta estimate of main effect
beta_se	standard error of beta estimate
empirical_feature_p_value	permutation p value

p_value	p value
global_adj_p_value	BH-adjusted P value
iteration	iteration (1 = primary)

Supplementary Table 12| caSNP TF motif disruption (in peak)

**Column Name**	**Description**
Chr	chromosome position
snpPos	snp position
strand	strand (+/-)
SNP_id	SNP ID
REF	reference allele
ALT	alternative allele
geneSymbol	motif gene symbol
dataSource	motif data source
providerId	JASPAR matrix ID
effect	disruption effect strength
motifPosition_start	start position of disrupted motif
motifPosition_end	end position of disrupted motif

Supplementary Table 13| List of response-caQTLs

**Column Name**	**Description**
Feature	peak ID
SNP	snp ID
PValue	p value
Interaction_Beta	interaction effect

Chr	chromosome position
Position	SNP position
global	BH-adjusted Pvalue
Peak_SNP	combined peak ID and snp ID
Ref	reference allele
Alt	alternative allele
RASQUAL_PValue_Veh	p value from vehicle RASQUAL output
RASQUAL_PValue_Condition	p value from condition RASQUAL output
Pi_Veh	cis-regulatory effect parameter (pi) from vehicle RASQUAL output
EffectSize_Veh	beta estimate of snp effect in vehicle
Pi_Condition	cis-regulatory effect parameter (pi) from condition RASQUAL output
EffectSize_Condition	beta estimate of snp effect in condition

Supplementary Table 14| List of response-eQTLs

A. WNT3A vs Vehicle, B. CHIR vs Vehicle

**Column Name**	**Description**
gene_name	HGNC gene name
chromosome	chromosome position
start	start position of gene
end	end position of gene
snp_id	SNP ID
Assessed Allele	assessed allele
maf	minor allele (assessed allele) frequency
beta	interaction effect
beta_se	standard error of interaction effect
p_value	P value of interaction effect

padj	BH-adjusted Pvalue

Supplementary Table 15| TFBS motif enrichment in response-caPeaks

A. WNT3A vs Vehicle response-caPeaks, B. CHIR vs Vehicle response-caPeaks

**Column Name**	**Description**
TF_name	Name of transcription factor binding site motif (JASPAR 2022)
TFID	JASPAR 2022 ID for transcription factor binding site
pval	P-value for logistic regression fit from r/glm() indicates significance of enrichment in response vs. non-response caPeaks
estimate	Estimated coefficient for effect of caPeak type (response vs. non-response) on TFBS motif presence in the caPeak
estimate_error	Standard error of estimates
z	Scaled estimates (estimate/estimate_error)
fdr	BH-adjusted Pvalue

Supplementary Table 16| caQTL Regional Overlaps

**Column Name**	**Description**
CHROM	chromosome position
START	start position of HAQER
END	End position of HAQER
NAME	HAQER ID
Feature	caPeak ID
Haq_Index	Index of HAQER
Wnt_Index	Index of caPeak
Region	Overlap region type (HAQERs/HARs)
Response_caPeak	* indicates response caPeak

Supplementary Table 17| caQTL-eQTL colocalizations

Column Name	Description
gene_chr	chromosome position (eQTL)
gene_start	start position of gene
gene_end	end position of gene
gene_name	HGNC gene name
snp_id	SNP ID (eQTL)
Assessed Allele	assessed allele (eQTL)
maf	minor allele (assessed allele) frequency (eQTL)
beta	interaction effect (eQTL)
beta_se	standard error of interaction effect (eQTL)
p_value	P value of interaction effect (eQTL)
global_adj_p_value	BH-adjusted Pvalue (eQTL)
Feature	peak ID
rs.ID	SNP ID (caQTL)
Chromosome	chromosome position (caQTL)
SNP.Position	SNP position (caQTL)
Ref.Allele	reference allele (caQTL)
Alt.allele	alternative allele (caQTL)
Effect.Size	cis-regulatory effect parameter (pi)
PValue	p value (caQTL)
Distance_from_PeakCenter	distance of tested SNP from the center of the peak
R2	*r2* between eQTL and caQTL SNP

Supplementary Table 18| GWAS colocalization results (caQTL)

Column Name	Description
chr	chromosome
Peak	peak ID
Wnt_Pos	caQTL index position
Wnt_SNP	caQTL SNP ID
GWAS_Pos	GWAS index Position
GWAS_SNP	GWAS SNP ID
R2_CurrentStudy	r^2 between GWAS index SNP and eQTL index SNP estimated in this study population
R2_1kgEUR	r^2 between GWAS index SNP and eQTL index SNP estimated in 1KG EUR population
GWASID	GWAS phenotype(s)

Supplementary Table 19| GWAS colocalization results (eQTL)

Column Name	Description
condition	stimulus condition (veh/WNT3A/CHIR)
gene	HGNC gene name
chr	chromosome
WntPos	eQTL index position
GWAS Pos	GWAS index Position
r2_current_study	r^2 between GWAS index SNP and eQTL index SNP estimated in this study population
r2_1kgEUR	r^2 between GWAS index SNP and eQTL index SNP estimated in 1KG EUR population
WntindexSNP	eQTL index SNP id
Pheno2indexrsID	Overlapping GWAS index SNP rsid
gtxSigInLocus_Brain_Cortex	TRUE: significant GTEx bulk tissue eQTL detected within $r^2 > 0.6$ of eQTL index SNP<br>FALSE: significant GTEx bulk tissue eQTL NOT detected within $r^2 > 0.6$ of eQTL index SNP
gtxSigInLocus_Brain_Frontal_	As above for GTEx Brain_Frontal_Cortex_BA9 tissue

Cortex_BA9	
gtxSigInLocus_Adipose_Subcutaneous	As above for Adipose_Subcutaneous

Supplemental Information References

1. Luo, Y. *et al.* New developments on the Encyclopedia of DNA Elements (ENCODE) data portal. *Nucleic Acids Res.* **48**, D882–D889 (2020).
2. Bourgon, R., Gentleman, R. & Huber, W. Independent filtering increases detection power for high-throughput experiments. *Proc. Natl. Acad. Sci. U. S. A.* **107**, 9546–9551 (2010).
3. Love, M. I., Huber, W. & Anders, S. Moderated estimation of fold change and dispersion for RNA-seq data with DESeq2. *Genome Biol.* **15**, 550 (2014).
4. Doumpas, N. *et al.* TCF/LEF dependent and independent transcriptional regulation of Wnt/β-catenin target genes. *EMBO J.* **38**, (2019).
5. Liang, D. *et al.* Cell-type-specific effects of genetic variation on chromatin accessibility during human neuronal differentiation. *Nat. Neurosci.* **24**, 941–953 (2021).
6. Aygün, N. *et al.* Brain-trait-associated variants impact cell-type-specific gene regulation during neurogenesis. *Am. J. Hum. Genet.* **108**, 1647–1668 (2021).
7. Roadmap Epigenomics Consortium *et al.* Integrative analysis of 111 reference human epigenomes. *Nature* **518**, 317–330 (2015).
8. McLean, C. Y. *et al.* GREAT improves functional interpretation of cis-regulatory regions. *Nat. Biotechnol.* **28**, 495–501 (2010).
9. Grasby, K. L. *et al.* The genetic architecture of the human cerebral cortex. *Science* **367**, (2020).
