# Supplementary material for: WNT activity reveals context-specific genetic effects on gene regulation in neural progenitors
Source: bioRxiv. 2023 Feb 7:2023.02.07.527357. Preprint. [Version 1] doi: 10.1101/2023.02.07.527357 (PMC9934631; doi:10.1101/2023.02.07.527357)
Supplement: Supplementary file 1 [file media-1.pdf]

Supplementary Information

Materials and Methods 3
hNPC culture 3
hNPC Wnt activity luciferase assays 3
hNPC proliferation assays 4
ATAC-seq Library preparation 4
RNA-seq Library Preparation 4
Sequencing of ATAC-seq and RNA-seq libraries 5
Genotyping and imputation 5
ATAC-seq data preprocessing 5
Chromatin Accessibility Peak Calling 6
Differential chromatin accessibility analysis 6
RNA-seq data pre-processing and analysis 6
Pathway enrichment analysis on differential expressed genes 7
Motif Enrichment Analysis 7
Correlation between chromatin accessibility and gene expression 7
Disease enrichment analysis using differentially expressed genes 7
Partitioning GWAS heritability by WREs 7
caQTL and response-caQTL mapping 8
cis-eQTL and response-eQTL mapping 9
Effects of ca/eQTLs on transcription factor binding sites 9
Shared eSNPs/caSNPs and GWAS SNPs 10

Supplementary Figures 11
Supplementary Figure 1| Multidimensional scaling analysis of genotype data 11
Supplementary Figure 2| Effects of CHIR and WNT3A on proliferation and Wnt activity 12
Supplementary Figure 3| Technical reproducibility of RNA-seq and ATAC-seq 14
Supplementary Figure 4| ATAC-seq Quality Control 15
Supplementary Figure 5| Global gene expression and chromatin accessibility patterns 16
Supplementary Figure 6| Number of differentially accessible regions and differentially expressed genes 17
Supplementary Figure 7| Wnt-pathway related transcription factor binding sites are enriched in upregulated WREs 18
Supplementary Figure 8| Stimulus-specific regulatory element-gene correlation 19
Supplementary Figure 9| Opposing gene regulation by WNT pathway inhibition 20
Supplementary Figure 10| ca/eQTL effect sizes compared to previous data 21
Supplementary Figure 11| eQTL-caQTL overlaps 22
Supplementary Figure 12| Effect size differences across conditions 23
Supplementary Figure 13| Colocalization of ANKRD44 r-eQTL, schizophrenia, and the volume of a hippocampal subfield (presubiculum body) 24

Supplementary Figure 14| Colocalization of an r-caQTL in DPYSL5 region and average thickness of the isthmus Cingulate 25
Supplementary Figure 15| Shared and context-specific QTL GWAS overlaps confirmed by eCAVIAR 26
Supplementary Figure 16| Stimulus-specific GWAS colocalization of FADS3 and Bipolar disorder 27
Supplementary Figure 17| Stimulus-specific ENO4 eQTL colocalizing with regional cortical surface area GWAS 28
Supplementary Figure 18| Optimizing control for known and unknown technical confounding in eQTL 30

Supplementary Tables 31
Supplementary Table 1| Differentially accessible peaks 31
Supplementary Table 2| TF motif enrichment analysis 31
Supplementary Table 3| Differentially expressed genes 31
Supplementary Table 4| Pathway enrichment analysis 32
Supplementary Table 5| Summary of peak-gene correlation 33
Supplementary Table 6| Peak-gene pairs 33
Supplementary Table 7| Disease enrichment analysis results(DEG) 34
Supplementary Table 8| Summary of GWASs used in this study 34
Supplementary Table 9| Partitioned heritability analysis results 35
Supplementary Table 10| List of caQTL - caPeak 35
Supplementary Table 11| List of eQTL - eGene 36
Supplementary Table 12| caSNP TF motif disruption (in peak) 37
Supplementary Table 13| List of response-caQTLs 38
Supplementary Table 14| List of response-eQTLs 39
Supplementary Table 15| caQTL-eQTL colocalizations 39
Supplementary Table 16| GWAS colocalization results (caQTL) 40
Supplementary Table 17| GWAS colocalization results (eQTL) 41

References continued 41

Materials and Methods

hNPC culture

Ethics statement for human tissue-derived cell-lines

This study followed IRB regulations to derive human NPC cell-lines from prenatal tissue collected at the UCLA Gene and Cell Therapy facility following voluntary termination of pregnancy.

Generation of hNPC lines

Fetal brain tissue visually consistent with dorsal telencephalon morphology (flat and sheet-like) was collected at 14-21 gestation weeks from presumed neurotypical donors to derive primary human NPCs as previously described (*11*, *24*, *43*). To summarize, tissue was dissociated into single cells which were cultured as neurospheres before transfer to fibronectin (Sigma F1141) and Poly-L-Ornithine (Sigma P3655) coated plates. Neuronal differentiation was suppressed to maintain NPCs in a proliferative state by following previously established culture methods (*10*). After 2-3 passages, NPC lines were cryopreserved and transferred to UNC Chapel Hill. NPC media: Neurobasal A (Life Technologies 10888-022) supplemented with 100 µg ml^{-1} primocin (Invivogen ant-pm-2), 10% BIT 9500 (Stemcell Technologies 09500), 1% glutamax (100x; Life Technologies, 35050061), 1 µg ml^{-1} heparin (Sigma-Aldrich H3393-10KU), 20 ng ml^{-1} EGF/FGF (Life Technologies PHG0313/PHG0023), 2 ng ml^{-1} LIF (Life Technologies PHC9481) and 10 ng ml^{-1} PDGF (Life Technologies PHG1034).

hNPC cell culture and WNT-stimulation

We cultured cryopreserved hNPCs in batches of 8 cell lines per week, pseudorandomizing each experimental group for biological variables (sex and donor gestation week), and technical variables (passage number). Following a two-week expansion period, we plated 400k NPCs per well of a 6-well plate for each cell-line. Next, we exposed each well to either vehicle (Neurobasal A media supplemented with PBS+0.1%BSA and DMSO), 5nM Wnt3a in PBS+0.1% BSA, or 2.5uM CHIR in DMSO for 48h. All exposures were prepared such that equal volumes were applied for each stimulated condition, including a “balancer” solution composed of culture media, PBS+0.1% BSA, DMSO, and water in order to standardize diluents across all exposures. The well-position of each exposure was rotated every week of the experiment to minimize the potential effects of plate position. After 48h exposure to stimuli or vehicle, cells were lifted with Accutase (Thermo Fisher Scientific A1110501) for preparation of ATAC-seq and RNA-seq libraries. We generated 2-6 separately cultured replicates for each of 6 randomly selected donors to measure technical variation across the experiment. We utilized cell culture media, growth factors, additives, and WNT-stimulating exposures from the same manufacturer lots across the entire experiment, whenever possible, to mitigate potential batch effects. When it was not possible to use the same lot of reagent, this information was recorded. One individual (JMV) performed all hNPC cultures and exposures to stimuli to minimize variance in handling effects. The investigators were not explicitly blinded to the donor during cell culture or library preparation. However, the investigators did not have knowledge of donor genotype when performing cell culture or library preparation. Regular pre-assay screening detected no mycoplasma contamination (ATCC 30-1012K).

hNPC Wnt activity luciferase assays

Canonical Wnt pathway signaling activity was measured via a luciferase reporter assay (**fig. S2A-C**). After two weeks of hNPC culture expansion as described above, we lifted cells with 0.05% trypsin (Gibco 25300062) and plated 10k cells counted by hemocytometer per well of 96-well

plates (Corning 07-200-91). Cells were transduced with 10uL lentivirus carrying BAR:Luciferase and Tk:Renilla constructs 24 hours after plating. Plasmids were generous gifts from the lab of Ben Major (*59*) and lentivirus was generated as previously described (*58*). After a two-day incubation, cells were exposed with either vehicle, CHIR, or WNT3A as described above. 48 hours later, cell lysates were collected and luminescence measured using the Dual-Glo luciferase system (Promega E2920) on the GloMax Discover plate reader (Promega). β-Catenin induced BAR:Luciferase signal was normalized by Renilla luminescence from the constitutively active Tk promoter.

hNPC proliferation assays

After two weeks of hNPC culture expansion as described above, we lifted cells with 0.05% trypsin (Gibco 25300062) and plated 12.5k cells counted by hemocytometer per well of 96-well plates (Corning 3610). We generated 4 technical replicate wells for each concentration of Wnt-activating stimulus or vehicle, and 8 technical replicate wells for vehicle exposures per hNPC donor cell-line, distributing replicates across each culture plate to mitigate potential artifacts introduced by plate position. One day later, vehicle or Wnt activating stimuli were applied, as described above. 46 hours later, we exposed cells with 10 μM EdU with a 10% media addition for 2 hours. hNPCs were collected and fixed via a 10-minute incubation in 4% paraformaldehyde in phosphate-buffered saline. On these cells, we labeled total DNA content with FxCycle Far Red dye (Thermo Fisher Scientific F10347) and performed Click-iT EdU-incorporation assays (Thermo Fisher Scientific C10337) according to the manufacturer's protocols. We quantified EdU and FxCycle signals using the Attune NxT 96-well Flow Cytometer (Thermo Fisher Scientific) (**Fig. S2D-F**), or via high content imaging on the Nikon Eclipse Ti2 microscope followed by manual quantification with Fiji (**Fig. S2G-H**). Flow cytometry data was processed with FlowJo v10.7.1 and cell-cycle populations defined by automated gating with FlowDensity software (*60*). Detailed methods for analysis of flow cytometry can be found in previous work(*58*).

ATAC-seq Library preparation

Cells were lifted and counted to isolate 50k cells per sample to be used as input for the Omni-ATAC protocol for library preparation(*61*). To summarize, 50k nuclei from fresh NPCs were counted via hemocytometer and tagmented using TDE1 enzyme (Illumina 20034198) and tagmentation buffer composed of 20mM Tris-HCl pH 7.6, 10mM $MgCl_2$, and 20% Dimethyl Formamide. All libraries were PCR amplified (NEB M0544S) for 5 cycles, and then each library was further amplified for a sample-specific additional number of cycles (average 2 additional cycles across all samples) determined by a qPCR side reaction to avoid overamplification. Nextera-compatible unique dual-indexing primers barcoded each sample during amplification (Illumina 20027213). We purified libraries to remove primer dimers and eliminate fragments over 1000bp (Roche 07983298001). We validated ATAC-seq library quality before sequencing for a subset of samples by observing appropriate nucleosomal banding patterns via capillary gel electrophoresis on an Agilent 4150 TapeStation system (Agilent 5067-5584). We then pooled ~70 ATAC-seq libraries with unique barcodes in each of four pools and sent pooled libraries for multiplexed sequencing. One individual (BDL) generated all ATAC-seq library preparations in order to minimize batch effects introduced by handling variance.

RNA-seq Library Preparation

Total RNA was isolated from each hNPC line and condition using all cells remaining (average 1.1M cells per sample) after removing 50k cells for ATAC-seq library preparation as described above. For each experimental week, hNPCs were lifted with Accutase (Thermo Fisher Scientific A1110501), lysed, and stored in TriZol (Thermo 15-596-026) at -80C for subsequent purification of total RNA using RNeasy Mini Kits and on-column DNase digestion (Qiagen 74106, 79256). All RNA extractions were performed by the same individual (JTM) to minimize potential batch effects introduced by handling. We quantified RNA isolates using fluorescence-based Quant-iT RNA assay kits (Thermo Q33140). Mean RNA integrity number (RIN) across all samples was 9.86 (SD = 0.39). Library preparation was performed using a stranded total RNA kit (KAPA KR0934) after samples were depleted of ribosomal RNA (KAPA KR1151).

Sequencing of ATAC-seq and RNA-seq libraries

ATAC-seq libraries were sequenced at the NYGC to an average read depth of 49M (SD = 13.6M) paired-end reads (2x100bp) per sample on the Illumina Novaseq platform. Total RNA-seq libraries were sequenced at the NYGC to an average sequencing depth of 55M (SD = 13M) paired-end reads (2x100bp) per sample on the Illumina Novaseq platform.

Genotyping and imputation

hNPC genotypes were obtained from genomic DNA isolated with DNeasy Blood and Tissue Kit (QIAGEN 69504) using Illumina's HumanOmni2.5 platform. Additional variants were imputed using the TOPMed freeze 5 reference panel (*62*) with minimac4 software (*63*) on the University of Michigan Imputation server. We performed quality control, pre-processing, and filtering of SNPs with PLINK v1.9(*64*) as previously described (*10*) based on Hardy-Weinberg equilibrium, minor allele frequency, individual missing genotype rates, and variant missing genotype rate (plink --hwe 1e-6 --maf 0.01 --mind 0.1 --geno 0.05).

ATAC-seq data preprocessing

FastQC (v0.11.9) (*65*) and MultiQC (v1.7) (*66*) software performed quality control for all ATAC-seq libraries before and after trimming sequencing adapters with BBMap (v38.98)(*67*). We then mapped ATAC-seq reads to the hg38 reference genome with the Burrows-Wheeler Alignment tool (BWA-MEM v0.7.17) (*68*). We then refined alignments to minimize mapping bias at sites where ATAC-seq reads overlapping bi-allelic SNPs using imputed genotypes by re-mapping and removing duplicate reads with WASP (v0.3.4) (*69*). Samtools (v1.16) (*70*) then removed unmapped or mitochondrial reads and bedtools (v2.3) (*71*) removed reads mapped to genomic blacklist regions as defined by ENCODE (*72*). ATAC-seq read metrics during and following preprocessing were calculated using picard (v2.21.7) (*73*) and ataqv software (v1.0.0)(*74*) (**fig. S4**). We evaluated cross-sample contamination using verifyBAMID (*75*), and omitted samples with FREEMIX or CHIPMIX scores greater than 0.02. We also omitted samples with low transcriptional start site enrichment (TSSe < 5) and aberrant short read/mononucleosomal read ratios (S/M <1 or S/M > 7). In total, 51 samples were omitted by these criteria. Principal component analysis (PCA) on ATAC-seq samples separated samples by stimulus condition, and not by sex or other biological or technical variables in the first two principal components (PCs) of variance (**fig. S5**). Sex and Donor ID, which captures cell-line intrinsic effects for each hNPC showed some correlation with other PCs, leading us to include these variables in QTL models described below and use residualized data following regression of PCs 1-10.

Chromatin Accessibility Peak Calling

After selecting a single ATAC-seq replicate for each donor-condition pair based first on whether stimulated conditions and vehicle were cultured in the same plate, and subsequently on QC metrics, we called chromatin accessibility peaks from ATAC-seq reads using all samples excluding technical replicates with CSAW's (v1.28)(*76*) *windowCounts* function with the options (ext = *mean length of fragments*, filter = *5 * sample number* spacing = *10*, param = pe.param) where pe.param were *max.frag = 1500, pe='both', minq=20*. Windows within 100bp were merged with the *mergeWindows* function. Read counts were normalized accounting for GC-content via conditional quantile normalization with cqn software (*77*).

Differential chromatin accessibility analysis

We ran DEseq2 for the read counts described above to identify peaks with differential accessibility due to stimulus condition, called WREs. The comparisons were performed as paired tests within donor, hence, the model was set to *Peak Accessibility ~ Donor ID + stimulus condition*, where Donor ID and stimulus condition were factor variables. We repeated this analysis for each WNT stimulus condition vs vehicle pairs (e.g. WNT3A vs Vehicle and CHIR vs Vehicle) or WNT3A+XAV vs WNT3A. FDR-adjusted $P < 0.1$ was used as the significance threshold.

RNA-seq data pre-processing and analysis

Similarly to the preprocessing of ATAC-seq data described above, we initially screened RNA-sequencing libraries using FastQC and MultiQC. We then trimmed sequencing adapters and mapped reads to genes as annotated by Ensembl v104 for the hg38 reference genome using the STAR aligner (v2.7.7a) (*78*). We filtered samples with (1) low RIN score (<7), (2) low unique mapped rate (< 80%), (3) high mismatched rate (>50%), (4) high multi-mapping rates (>8%), (5) high duplicated read rates (> 30%), or (6) FREEMIX or CHIPMIX scores from verifyBAMID greater than 0.02. QC filtering by these criteria removed 27 samples and retained a total of 242 samples. Transcripts were collapsed using collapse_annotation.py (https://github.com/broadinstitute/gtex-pipeline/tree/master/gene_model) into a single gene model and gene-level reads were summarized for each sample using featureCounts from Rsubread (v2.8.2) (*79*). As with the ATAC-seq samples, we performed PCA and found that the RNA-seq samples clustered according to stimulus condition and not sex, RIN, or other variables in PC1 vs PC2 plots (**fig S5**). Again, some PCs correlated with hNPC donor sex and RIN, indicating that we should include these variables in the QTL models described below for data corrected for PCs 1-10. Significantly higher pair-wise correlations within RNA-seq sample technical replicates generated for the same hNPC donor and condition ("within donor") compared to correlation across distinct donors (Evaluated by the Welch two sample t-test) supports the technical reproducibility of the RNA-seq dataset (**fig S2**).

After selecting a single RNA-seq replicate for each donor-condition pair based first on whether stimulated conditions and vehicle were cultured in the same plate, and subsequently on QC metrics, DESeq2 (*80*) was used to assess differential gene expression from RNA-seq reads mapped to protein-coding genes or lncRNA where at least 1% of samples in either condition show at least 10 normalized counts. We evaluated the model *RNAseq Count ~ RIN + Donor ID + stimulus condition* to identify DEGs. In this model, we represented RIN as a numeric variable and Donor ID and stimulus condition as factor variables. Shrunken log2 fold change (LFC) (*81*) was used for estimating dispersions and FDR-adjusted *P* value < 0.1 was used as significance threshold.

Pathway enrichment analysis on differential expressed genes

To determine pathways enriched in DEGs, we first obtained up-/ down-regulated genes in WNT stimulus condition as compared to vehicle (shrunken LFC > 0, < 0, respectively; and FDR-adjusted $P < 0.1$). All genes included in DEG analysis were used as the background. For both DEG and background genes, we restricted the analysis to protein-coding genes located outside the MHC region (chr6:28,510,120-33,480,577). Analysis was run by g:COSt from g:profiler (*82*) for KEGG and REACTOME pathways (*83*, *84*). FDR-adjusted $P < 0.1$ was used as the statistical significance threshold.

Motif Enrichment Analysis

We tested differential transcription factor (TF) motif enrichment within WREs for 841 predicted human TFs in JASPAR 2022 (*85*) core database (taxonomic group = vertebrates), using data downloaded from http://expdata.cmmt.ubc.ca/JASPAR/downloads/UCSC_tracks/2022/hg38/ (data access date: May 4th 2022). We restricted analysis to TFBS motifs within conserved regions, defined by 100-way phastCons scores > 0.4 (*86*) downloaded from UCSC Genome Browser in regions ≥ 20bp. We estimated TFBS motif enrichment within the top 2,000 chromatin accessibility peaks representing WREs gained or lost in each stimulus condition (|LogFC| at FDR-adj $P < 0.1$ as compared to vehicle) to avoid bias introduced by the different number WREs across conditions. Finally, we ran a logistic regression to identify TF motifs observed more often in gained peaks or lost peaks while accounting for differences in peak width and percentage of peaks in conserved regions using the following model: *glm(TFBS ~ peaktype + peadkwidth + conservedbppercent, family ='binomial'),* where *TFBS* is a binary outcome that indicates whether each differentially accessible peak overlaps with TF binding sites, *peaktype* indicates open or closed status of peak due to stimulation condition, *peadkwidth* indicates window size of peak and *conservedbppercent* indicates percentage of conserved regions (*conserved bp / peak width*). We considered *peaktypes* with TFBS enrichments with FDR-adjusted $P < 0.1$ as statistically significant.

Correlation between chromatin accessibility and gene expression

We estimated Pearson's correlation between chromatin accessibility and gene expression using variance stabilizing transformation (VST) normalized ATAC-seq and RNA-seq read counts residualized by 10 global chromatin peak or gene expression PCs, respectively. For each condition, we tested all peaks located within 1MB from the transcription starting site (TSS) of each gene. FDR-adjusted $P < 0.1$ was used for the statistical significance threshold.

Disease enrichment analysis using differentially expressed genes

We performed disease enrichment analysis for each DEG category (upregulated/downregulated/all-regulated/non-DEGs) using disease_enrichment() function implemented in disgenet2r (*39*). Disease enrichments referenced "CURATED" genes from 4,254 diseases from the DisGeNET database (*39*), and we classified results with FDR-adjusted $P < 0.1$ generated by the disease_enrichment() function as significant. Due to limited space, we only presented diseases if disease_semantic_type is either "Mental", "Behavioral Dysfunction", "Individual Behavior" or "Mental Process" in **Fig. 2**. Full lists are provided in **table S7**.

Partitioning GWAS heritability by WREs

We first assessed the contribution of regulatory elements including tissue-type-specific or cell-type-specific regulatory annotations to the overall heritability of brain-related and other traits (**table S7**). Analyses were performed using Stratified LD Score regression (S-LDSC) (*42*, *87*) (v1.0.0) including the baseline-LD model (v1.2). Annotations included (1) Active enhancer or promoter states in fetal brains (female/male). Active enhancer and promoter regions were defined based on chromatin states predicted by chromHMM (*88*) and included: 'active transcription start site', 'flanking active TSS', 'genic enhancers', and 'enhancers' in the core 15-state model (https://egg2.wustl.edu/roadmap/web_portal/chr_state_learning.html). (2) Progenitor-specific chromatin accessible regions were defined as logFC > 0 and FDR-adjusted *P* < 0.1 as a comparison to differentiated neuronal cells. ATAC-seq data were obtained from our previous work (*43*). (3) Open or closed chromatin accessible regions by stimulus condition compared to vehicle conditions were defined by logFC > 0 or logFC < 0, FDR-adjusted *P* < 0.1 and identified in the current study as WREs. For (3), we included non-WREs (FDR-adjusted P > 0.1) in the model to test the specificity of WREs.

We obtained publicly available GWAS summary statistics for testing partitioned heritability. The details of GWAS are provided in **table S8**. For each GWAS summary statistics, SNPs were filtered to those found in HapMap3 (*89*) using munge_sumstats.py provided by LDSC. The pre-computed LD scores for the European population from the 1,000 Genome Project Phase 3 (1KG) (*90*). were downloaded from https://data.broadinstitute.org/alkesgroup/LDSCORE/eur_w_ld_chr.tar.bz2. Enrichment was assessed using *P* values calculated by the annotation's standardized effect size (tau) that was conditioned on other annotations included in the model. FDR-adjusted *P* < 0.1 was used for statistical significance threshold.

caQTL and response-caQTL mapping

caQTL analyses within each condition (Vehicle, WNT3A, CHIR) were performed using the joint model implemented in RASQUAL (*91*) which integrates both between-individual signals and allele-specific signals within individuals. Previously described cqn normalization factors were used as sample specific offsets. Covariates were included consisting of 10 count-based principal components and 10 multidimensional scaling (MDS) genotype components to reduce the confounding effects of technical factors and population structure, respectively. Variants were tested according to the following criteria (1) in a given accessible region or within +/- 25kb (2) minor allele frequency (MAF) ≥ 1% (3) Hardy-Weinberg equilibrium < 0.000001 (4) imputation quality ≥ 0.3 (5) at least two individuals have minor homozygous allele or heterozygous allele.

Multiple testing correction were completed following a hierarchical correction procedure (*11*, *92*) consisting of (1) adjusting nominal p-values of all SNPs for each peak separately using the eigenMT method (*93*) (2) BH procedure was then applied to these locally adjusted p-values to determine globally adjusted p-values lower than 0.1 (3) To determine other independent SNPs for each peak, the maximum nominal p-value from step 1 corresponding to a globally adjusted p-value of 0.1 was used as a significance threshold.

Due to limitations of RASQUAL, conditional analysis, where the top caSNP was controlled for in the association model to find additional independent signals, was not possible. To determine signals most likely to be distinct from the lead for each peak a LD threshold of < 0.2 was used iteratively until no additional significant caQTL remained.

Response caQTL interaction analyses between vehicle and each WNT-stimulus condition (WNT3a or CHIR) used the R package lme4 to evaluate the following linear mixed model for all index caQTL with main effects (n_{caSNP} = 136,163 (WNT3A_Vehicle pairs), 136,641 (CHIR_Vehicle pairs)): $adjCounts = SNP + condition + condition{:}SNP + covariates + (1|Donor)$,
where condition = 0 or 1 (Veh or WNT activators, respectively). The first 10 count-based PCs were used as covariates. We compared the fit of the model above with and without the

condition:SNP interaction term to assess significance. BH was then used to adjust for multiple testing (FDR-adjusted *P* < 0.1)

cis-eQTL and response-eQTL mapping

Cis-eQTL analyses within each condition (Vehicle, WNT3A, CHIR) were performed using a linear mixed model implemented in limix_qtl ((*94*); https://github.com/single-cell-genetics/limix_qtl), an optimized version of limix (*95*). The following genes and SNPs were tested: *Gene*: (1) at least 1% of samples in the condition have at least ≥ 10 normalized counts; (2) protein-coding gene or lncRNA; *SNP*: (1) located in gene body or within +/- 1MB from gene body. (2) minor allele frequency (MAF) ≥ 1% (3) Hardy-Weinberg equilibrium < 0.000001, (4) imputation quality ≥ 0.3, (5) at least two individuals have minor homozygous allele or heterozygous allele. We used the following model: $adjE = SNP + \mu_k + \varepsilon$, where *adj*E is VST-normalized gene read count and further corrected with 10 principal components across the gene expression matrix, that were chosen to maximize the number of eQTLs (**fig. S16**), SNP is a genotype (0/1/2), u_K is a kinship matrix and ε is an error term. The kinship matrix was generated by pcrelate() function in GENESIS (v2.14.1) (*96*) with 10 PCs to control ancestry population structure.

Gene-level multiple corrections were done for each gene by permutation test implemented in limix_qtl, then applied BH-FDR for global correction. eSNP-eGene pairs with FDR-adjusted *P* < 0.1 were considered as statistically significant eQTLs. Those eGenes were further tested for independent signals through conditional testing, whereby the index eSNP is included as a covariate in the association model. For visualization purposes, we set the significance line threshold for loci with no significant eQTL in the condition as follows. First, we obtained the maximum permutation test p-value satisfying FDR-adjusted *P* < 0.1 (max_permP). Then, we estimated the median of the maximum raw p-value less than max_permP across eGene as the significance threshold.

Response eQTL interaction analyses between vehicle and each WNT-stimulus condition (WNT3A or CHIR), were also performed by limix_qtl for all index eQTLs with main effects (n_{eSNP} = 2,966 (WNT3A_Vehicle pairs), 2,906 (CHIR_Vehicle pairs)). The interaction term for the model
$adjE = SNP + \mu_k + condition + condition{:}SNP + \varepsilon$
was tested to identify interaction effects between SNP and condition where condition = 0 or 1 (Vehicle or WNT activators, respectively). We used BH to adjust multiple testing (FDR-adjusted P < 0.1).

Effects of ca/eQTLs on transcription factor binding sites

We used motifBreakR (v.2.6.1) (*97*) to assess the impact of genetic variants within peaks on TF binding motifs surrounding significant caSNP-caPeak associations (parameter setting, threshold of $1x10^{-4}$) (*98*) (**table S12**). Annotated motifs (839 total TF motifs) from JASPAR2022 vertebrate were used in MotifDb (v1.37.1) (*99*). Relative entropy (parameter setting method, 'ic') for both reference and alternative allele was calculated and only TFBSs strongly affected by the SNPs (parameter setting effect, 'strong') were retained. caSNP sites with significantly associated motif disruptions were then assessed for patterns indicative of priming or stimulus-specific signals within each Vehicle-WNT stimulus pair. caQTL and eQTL SNP overlaps were determined where a caSNP was within 1Mb of the eSNP with an LD ≥ 0.8. Priming signals were called based on (1) significant caSNPs common to both Vehicle and WNT stimulus condition (2) only those which overlapped an eSNP signal (3) significant eSNP specific to WNT stimulus (4) confirmed expression of TF associated with significantly associated motifs. Stimulus-specific signals were

assessed following the same criteria but filtering to significant caSNPs specific to the WNT stimulus. Enrichment of priming and stimulus-specific caSNP-disrupted motifs in accessible peaks was calculated using the binomial test (*100*).

Shared eSNPs/caSNPs and GWAS SNPs

We assessed colocalization between causal eca/e-QTL variants and GWAS signals in brain-related traits (**tables S16** and **S17**) through a three-step process. First, we extracted GWAS SNPs ($P < 5 \times 10^{-8}$) that are located within 1MB from ca/eQTLs. Second, we selected GWAS-QTL pairs for which index SNPs are in LD ($r^2 > 0.6$) in either population (1KG EUR or samples used in this study). To consider the possibility of undefined secondary GWAS signals, we also selected GWAS-QTL pairs for which at least one non-index but genome-wide (GW) significant ($P < 5 \times 10^{-8}$) GWAS SNP was in LD with an eQTL index SNP. We tested two colocalization approaches and reported if either approach suggested colocalization. As the first approach, we estimated residual GWAS association statistics after conditioning ca/eQTL index SNPs. We performed approximate conditional analysis using default GCTA settings on variants within 1Mb of those variants (*101*, *102*). We used a subset of 40,000 European-ancestry (White British) UKBB participants for the LD reference panel (https://www.ukbiobank.ac.uk/). We excluded variants from GCTA output if the frequency of effect allele differed by >0.2 between UKBB and GWAS summary results, and masked approximate conditional results if variants exceeded a collinearity threshold of 0.9 with the ca/eQTL index SNP. The second approach was to use eCAVIAR (*103*). We calculated SNP-level colocalization posterior probability (CLPP) for each SNP in a locus that passed the previous two steps. CLPP was estimated by eCAVIAR (*103*) for SNP at $P < 0.05$ in both GWAS and QTL. We considered any locus with CLPP > 1% and r^2 between causal SNP and at least one of GW SNPs in the region > 0.8 as evidence of colocalization in this approach. False positives for QTL condition-specificity could occur due to insufficient power limited by sample size. Thus, we repeated eCAVIAR to salvage potentially shared eGene/caPeak-GWAS pairs that failed to meet FDR-adjusted significance thresholds ($P < 0.1$), but passed the raw significance threshold ($P < 10^{-6}$) for QTL mapping under a particular condition, and showed shared caPeak/eGene-GWAS pairs under a separate condition.

Supplementary Figures

Supplementary Figure 1| Multidimensional scaling analysis of genotype data

Multi-dimensional scaling (MDS) plot shows the first two components of genetic similarity for all HapMap populations and donors in this study, allowing inference of genetic ancestry. Multiple ancestries of donors are present in this population.

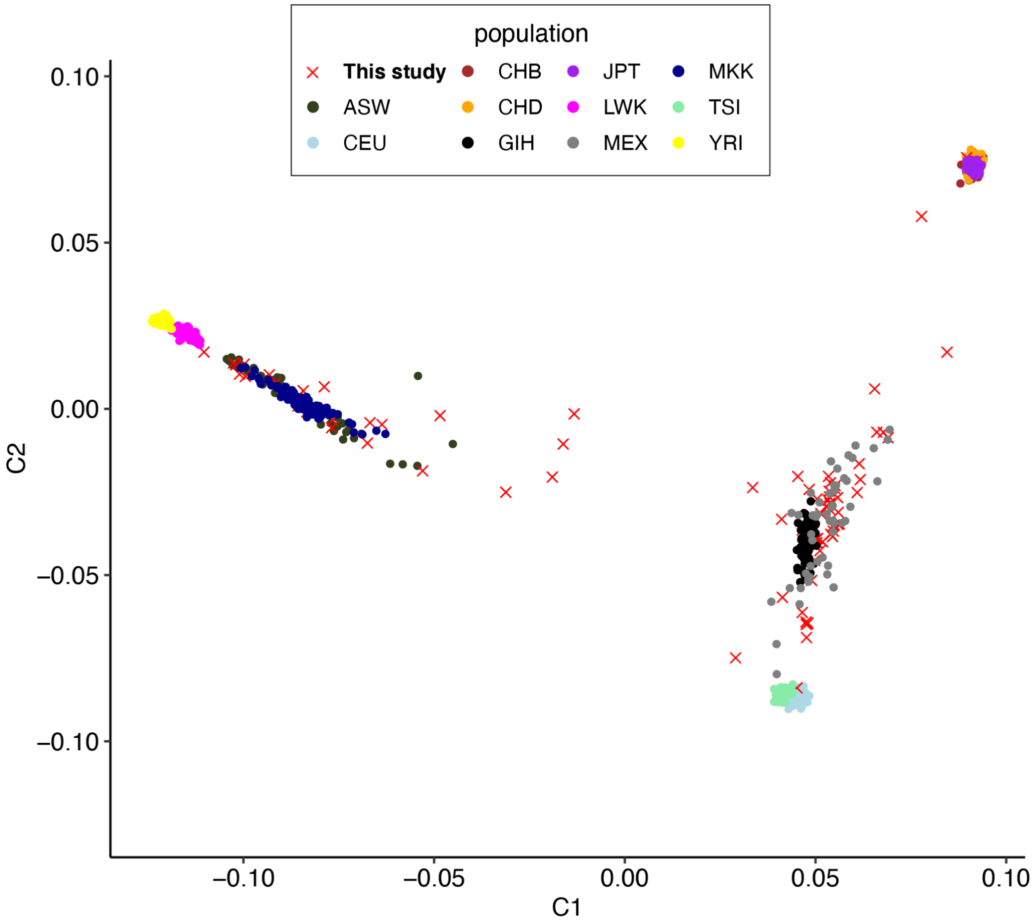

Supplementary Figure 2| Effects of CHIR and WNT3A on proliferation and Wnt activity

Cellular assays show that WNT3A and CHIR stimulation increase canonical Wnt pathway signaling and hNPC proliferation. Diagram of luciferase reporter assay measuring β-catenin mediated Wnt-pathway activation by CHIR or WNT3A (**A**). Effects of 48h CHIR (**B**) or WNT3A (**C**) exposure on Wnt pathway activation, reported as the log of luciferase luminescence (activated by Wnt stimulation) normalized by renilla luminescence (from a constitutively active reporter transgene). Representative flow cytometry scatter plots from proliferation assays depict newly synthesized DNA from a 2h hr EdU pulse vs total DNA content following 48h vehicle (left) or 2.5uM CHIR (right) exposure (**D**). Percentage of cells in S-phase (%EdU+) exposed to vehicle or increasing doses of CHIR for 48h as measured by flow cytometry in a subset of cell-lines (**E**), and in all cell-lines used for ATAC-seq and RNA-seq in this study (**F**). Representative immunocytochemistry images (100x magnification) from proliferation assay of hNPCs following 48h vehicle (left) and 5nM WNT3A (right) exposure **G**). Green (GFP) labels cells in S-phase during the EdU pulse, and blue (DAPI) stains all nuclei. Percentage of cells in S-phase (%EdU+) exposed to vehicle or increasing doses of WNT3A for 48h as measured by flow cytometry in a subset of cell-lines (**H**). CHIR and WNT3A concentrations (boxed) that maximize Wnt activation and proliferative responses were used in this study for ca/eQTL mapping.

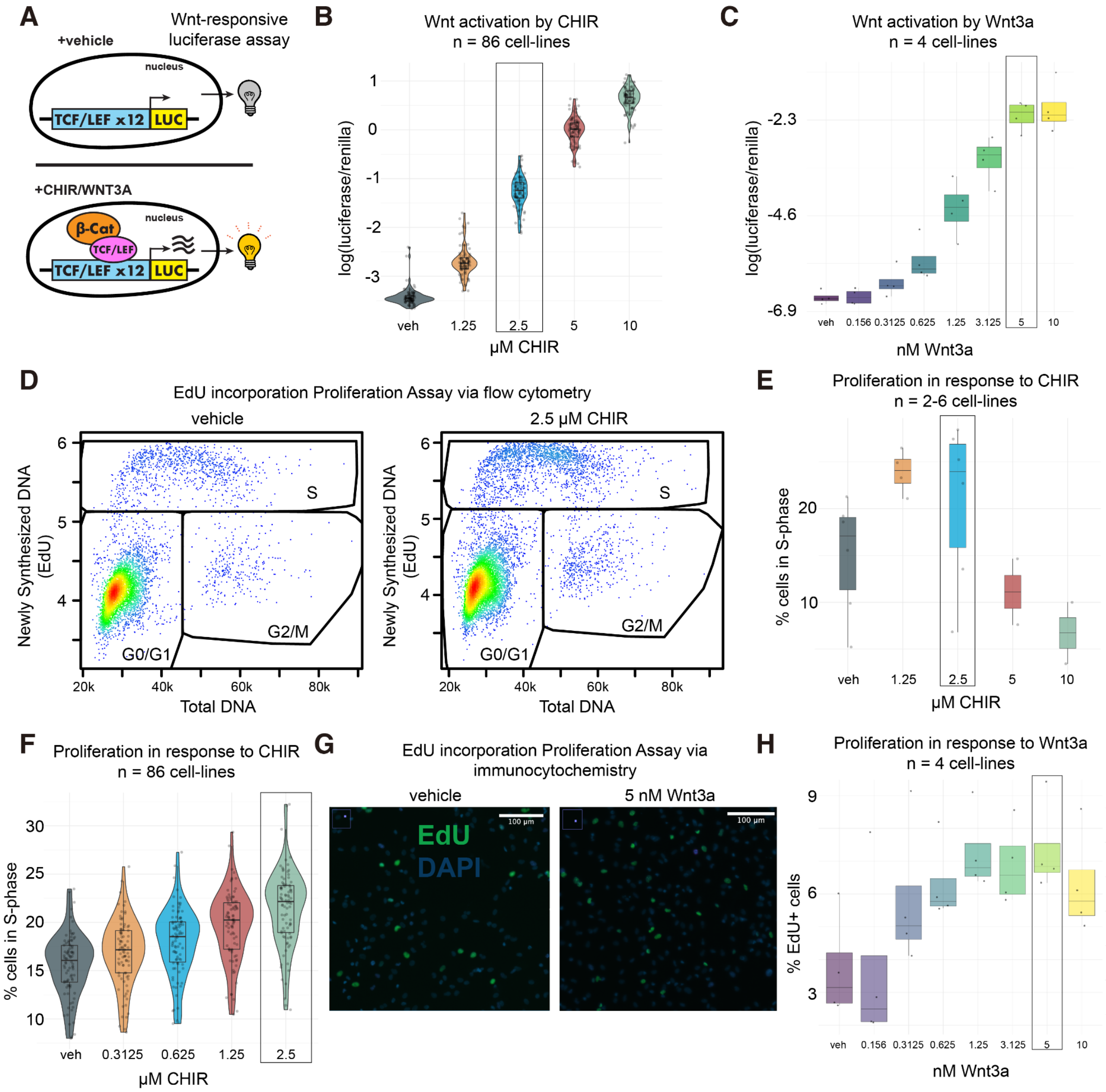
A
+vehicle
Wnt-responsive
luciferase assay
nucleus
TCF/LEF x12
LUC
+CHIR/WNT3A
β-Cat
TCF/LEF
B
Wnt activation by CHIR
n = 86 cell-lines
log(luciferase/renilla)
µM CHIR
veh
1.25
2.5
5
10
C
Wnt activation by Wnt3a
n = 4 cell-lines
log(luciferase/renilla)
-2.3
-4.6
-6.9
veh
0.156
0.3125
0.625
1.25
3.125
5
10
nM Wnt3a
D
EdU incorporation Proliferation Assay via flow cytometry
vehicle
2.5 µM CHIR
Newly Synthesized DNA (EdU)
S
G0/G1
G2/M
Total DNA
20k
40k
60k
80k
E
Proliferation in response to CHIR
n = 2-6 cell-lines
% cells in S-phase
µM CHIR
F
Proliferation in response to CHIR
n = 86 cell-lines
% cells in S-phase
veh
0.3125
0.625
1.25
2.5
µM CHIR
G
EdU incorporation Proliferation Assay via immunocytochemistry
vehicle
5 nM Wnt3a
EdU
DAPI
100 µm
H
Proliferation in response to Wnt3a
n = 4 cell-lines
% EdU+ cells
nM Wnt3a

Supplementary Figure 3| Technical reproducibility of RNA-seq and ATAC-seq

Pairwise correlations of normalized sequencing counts across open chromatin peaks measured by ATAC-seq (**A**) or genes measured by RNA-seq (**B**). Higher correlations for within donor vs across donor pairs indicates robust reproducibility of measurements for a given donor and condition. Violin plots represent the distribution of Pearson correlation coefficients calculated between a pair of genotypically distinct donors (“across donor”) or between two technical replicates of the same donor cultured at different times (“within donor”). Top and bottom horizontal lines within violin plots represent the interquartile range of the data, and the middle bar represents the median. P-values report significant differences between the across vs. within donor correlations following Fisher’s Z transformation and evaluated with the Welch two sample t-test. The total number of cell-lines and pairwise correlations (n) for each experimental condition is reported along the x-axis.

A

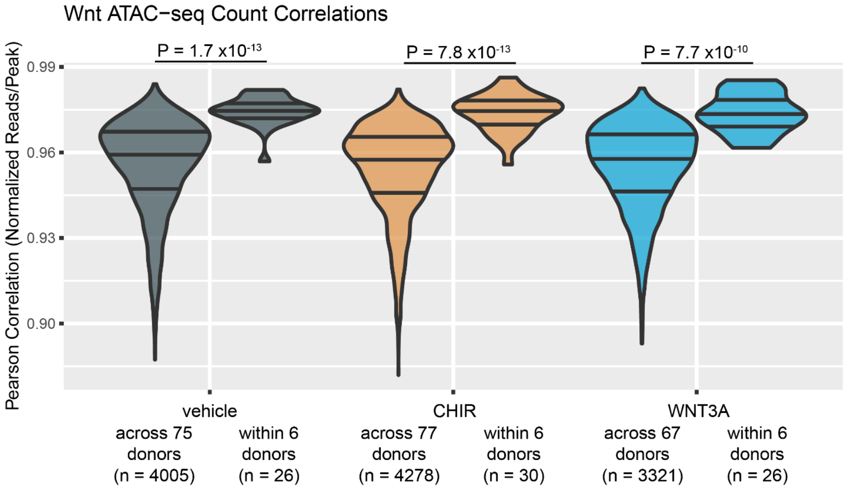

B

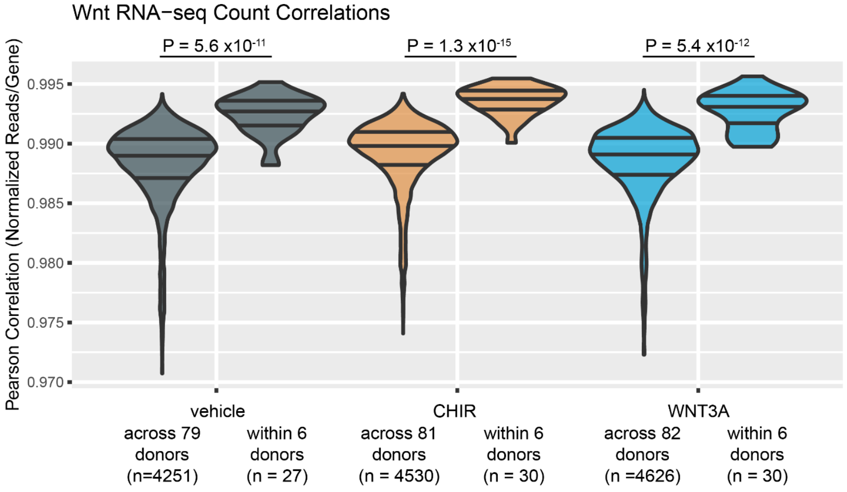

Supplementary Figure 4| ATAC-seq Quality Control

Total ATAC-seq reads (**A**), unique non mitochondrial reads (**B**), transcription start site (TSS) enrichment (**C**), total read duplication rate (**D**), mitochondrial read duplication rate (**E**), and fraction of reads within chromatin accessibility peaks (FRiP) (**F**) across stimulus conditions. Mean insert size distributions in base pairs across all samples colored by stimulus condition (**G**) exhibit a nucleosomal phasing pattern; right: insert size distribution plotted on log- scale. Mean TSS enrichments (> 7) and FRiP scores (>0.3) exceed the "ideal" metrics for ATAC-seq libraries defined by the ENCODE project (*72*). Together, these measurements validate the quality of ATAC-seq samples used in this study.

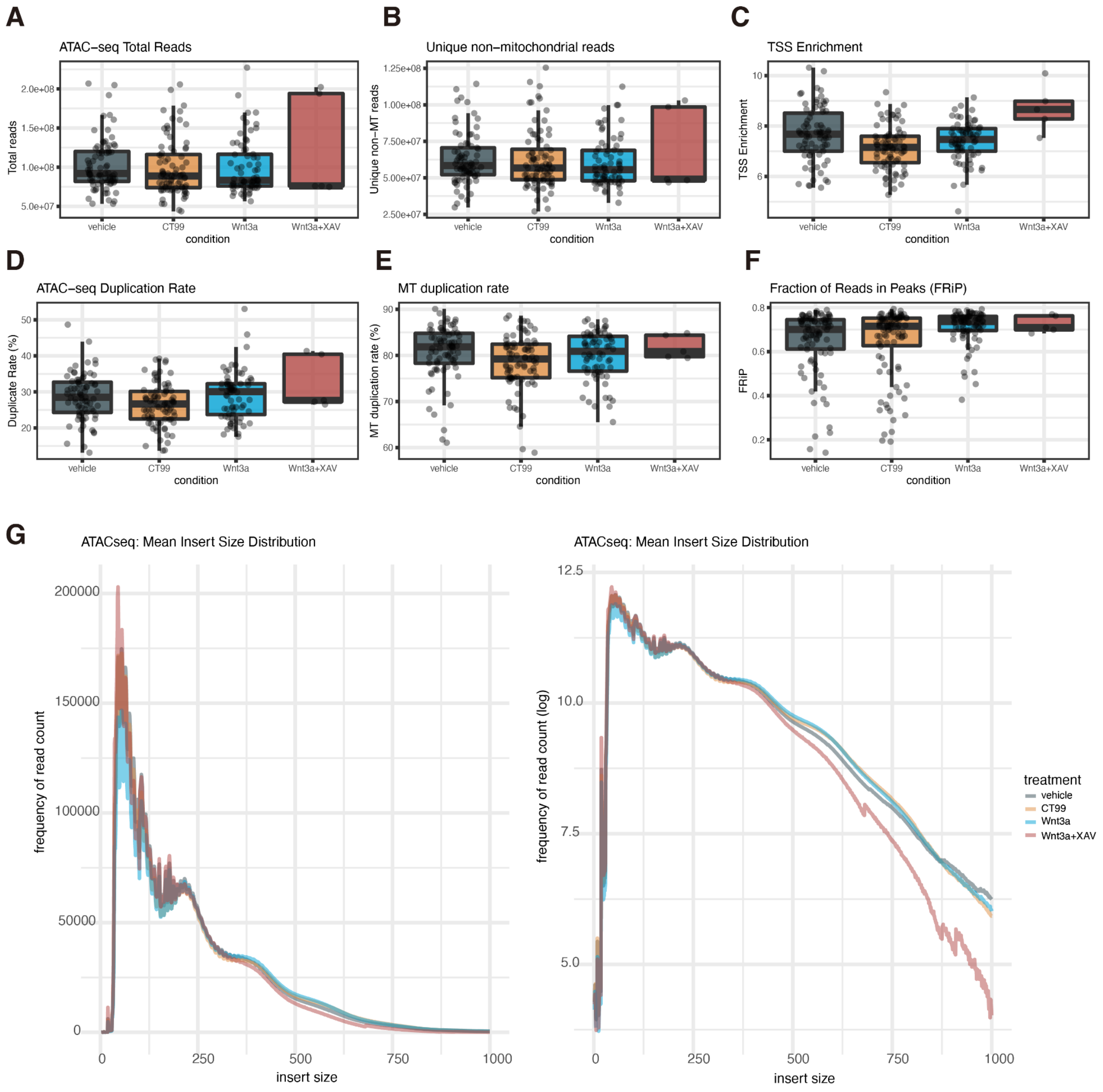

Supplementary Figure 5| Global gene expression and chromatin accessibility patterns

Principal component analysis (PCA) of ATAC-seq (**A, C**) and RNA-seq (**B, D**) data after batch correction for technical variables included in differential accessibility or expression models, respectively. Samples are labeled by condition (**A**, **B**), or sex (**C**, **D**). Variance in global gene expression and chromatin accessibility profiles across the first two PCs is driven by stimulation condition, but not by sex. Correlation matrix of ATAC-seq (**E**) or RNA-seq (**F**) principal components 1-10 with technical (RIN) and biological variables (donor and sex). We performed linear regression to remove effects of PCs 1-10 and the residualized sequencing count data was used as QTL model input to account for measured and unmeasured confounding.

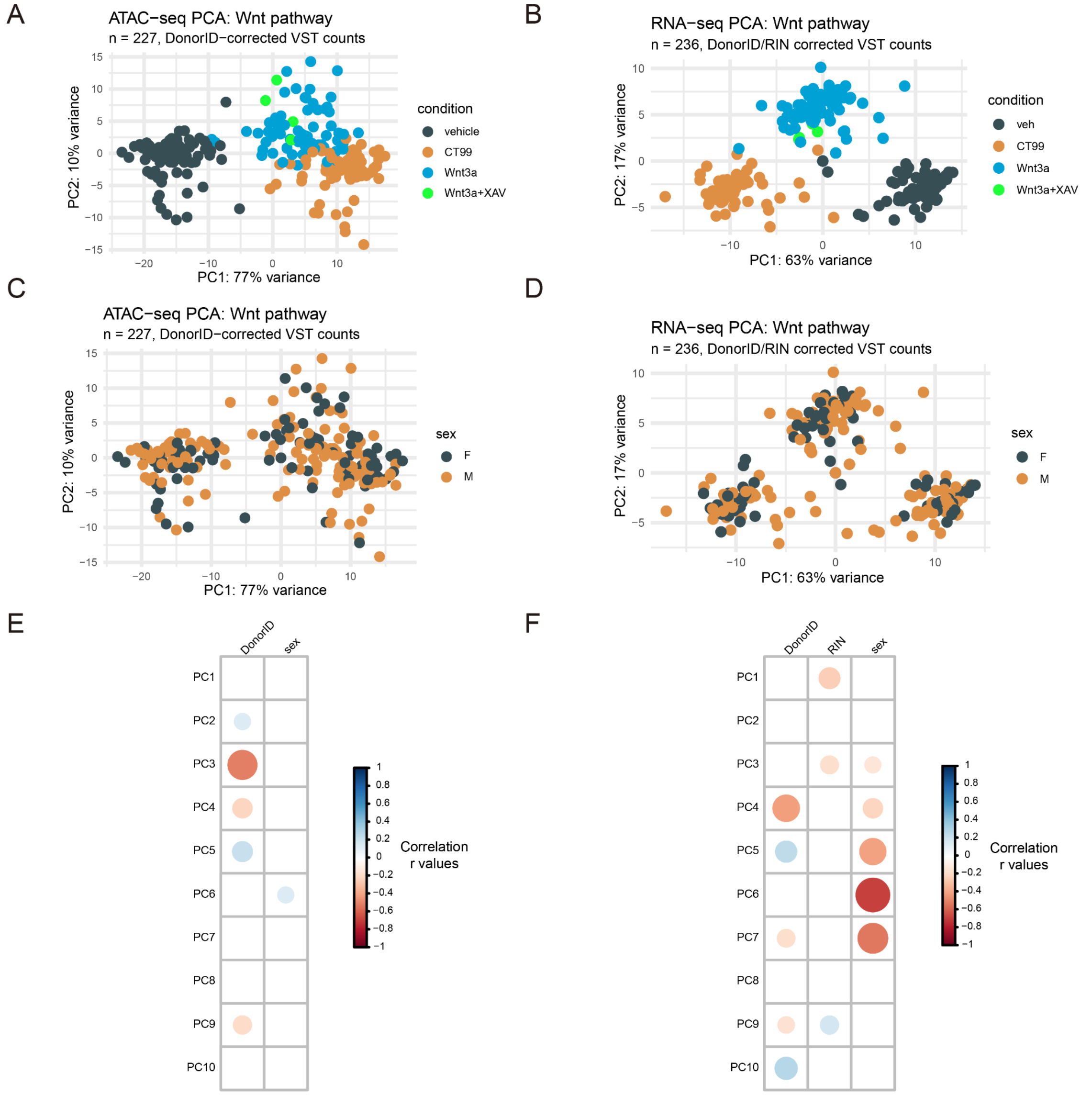

Supplementary Figure 6| Number of differentially accessible regions and differentially expressed genes

Barplots showing the number of differentially accessible regions (WREs) (**A**) and differentially expressed genes (DEGs) (**B**). The reference indicates referenced condition (either vehicle or WNT3A). Sample size indicates paired samples between conditions. Percentage of increased (shrunken log2FC > 0.5 in dark blue, shrunken log2FC > 0 in blue) or decreased (shrunken log2FC < -0.5 in red, shrunken log2FC < 0 in pink) accessibility or expression are shown for each category. Nonsignificant peaks and genes (FDR-adjusted P >=0.1) are colored gray. Greater changes were observed in both chromatin accessibility and gene expression for CHIR, a potent Wnt activator, as compared to WNT3A, an endogenous Wnt ligand. Simultaneous WNT3A activation and inhibition via XAV as compared to WNT3A activation yielded few differentially expressed genes and chromatin elements, likely due to the low sample size. We also note that 93,853 peaks and 9,890 genes were filtered out (adjusted *P* value were set to *NA*) by DESeq2 (*104*, *81*) as those have low mean read counts.

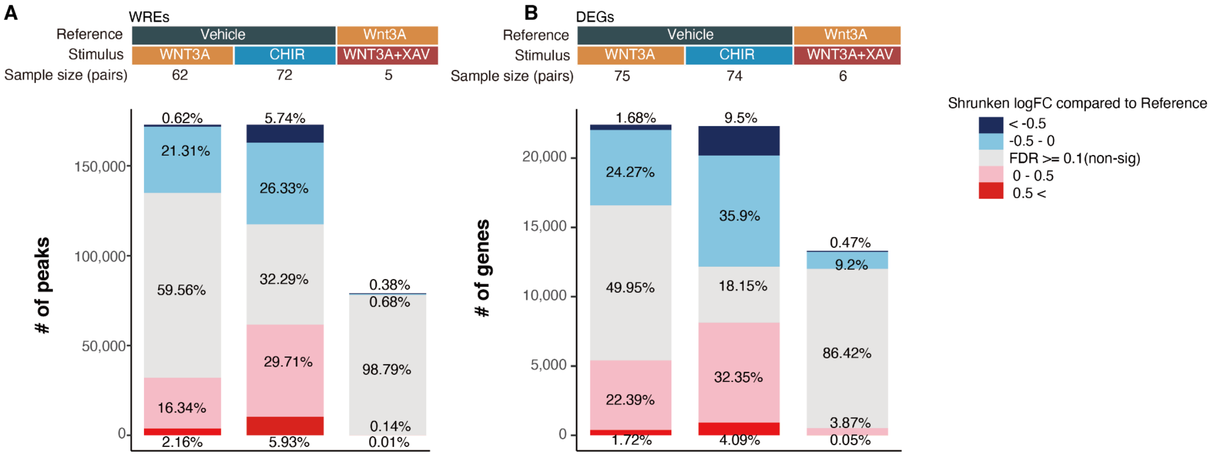

Supplementary Figure 7| Wnt-pathway related transcription factor binding sites are enriched in upregulated WREs

The canonical Wnt-pathway downstream effectors, ß-catenin, LEF1, and TCF7L2 binding sites previously identified by ChIP-seq experiments in HEK cells (*72*, *27*) are more often overlapped with WREs opening due to WNT3A (**A**) or CHIR (**B**) than closing WREs. The figures show the percentage of overlaps with the indicated TF binding sites in either the 2,000 most upregulated peaks or the 2,000 most downregulated peaks based on shrunken logFC. Statistical significance was estimated using Fisher's exact test on the number of overlaps.

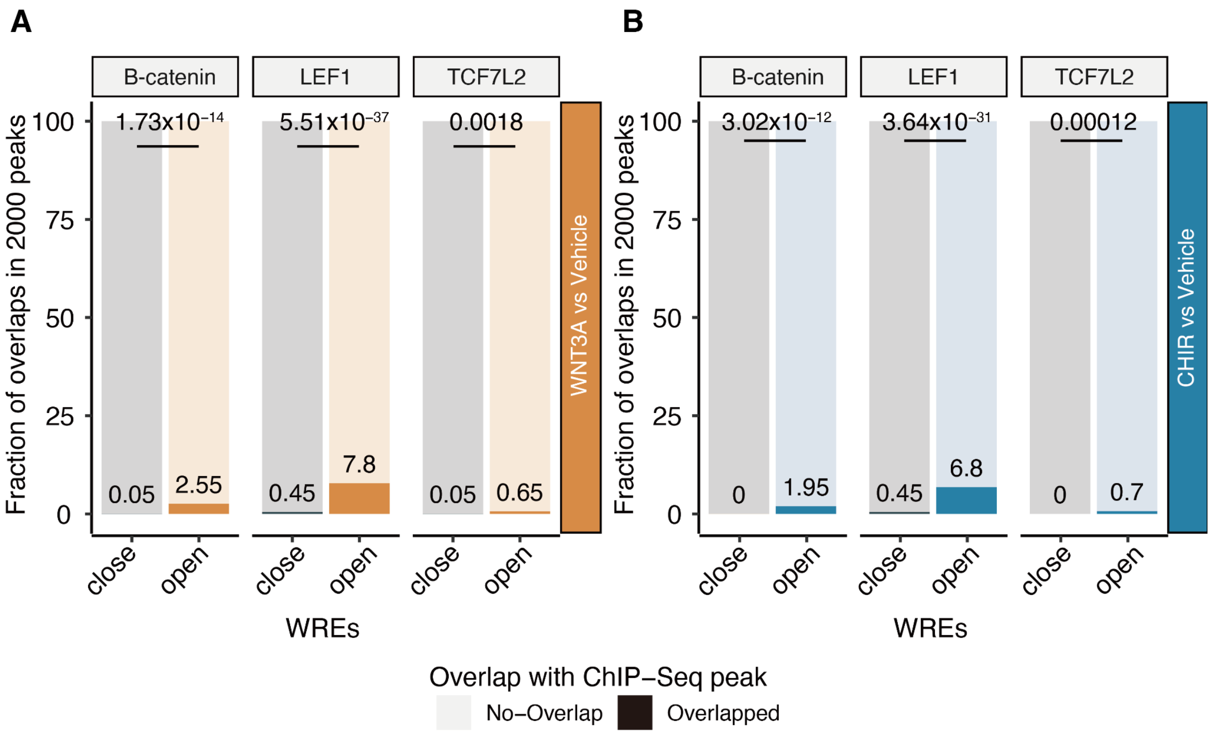

Supplementary Figure 8| Stimulus-specific regulatory element-gene correlation

We identified stimulus-specific regulatory elements which regulate gene expression by correlating expression with chromatin accessibility. The number in the plot indicates significant and positively correlated peak-gene pairs. In total, 12,643 peak-gene pairs are detected only under the stimulus condition (highlighted in red), demonstrating new stimulus-specific regulatory elements.

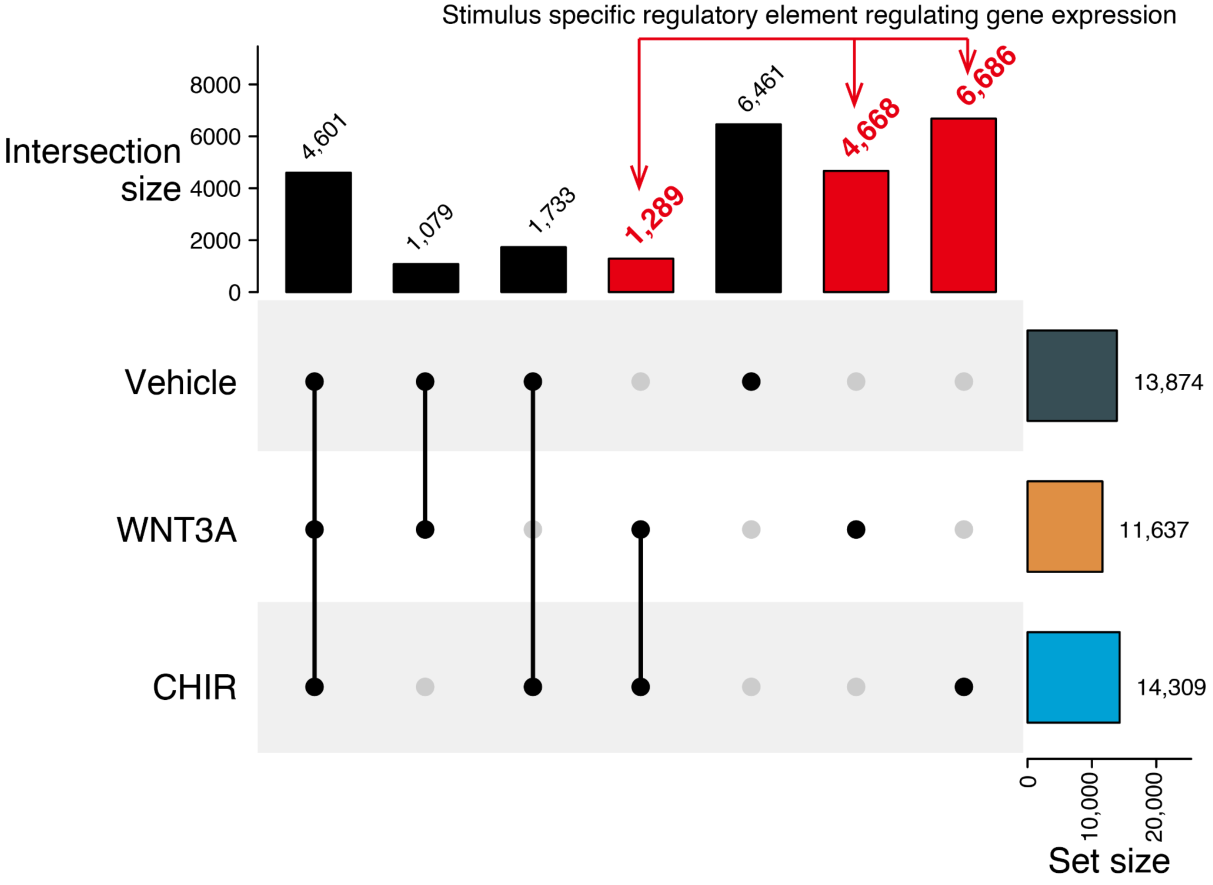

Supplementary Figure 9| Opposing gene regulation by WNT pathway inhibition

(**A**) Volcano plot showing differential expressed genes between simultaneous excitation and inhibition of WNT3A (WNT3A+XAV) as compared to WNT3A stimulus alone. Note that LEF1, a downstream effector of the Wnt pathways, has decreased expression under inhibition of the Wnt pathway. (**B**) Among 1413 genes that are differentially expressed at BH-adj P < 0.1 in both comparisons (WNT3A+XAV vs WNT3A (6 pairs) and WNT3A vs Vehicle (75 pairs)), 70.8% (1001 genes), opposing changes in gene expression were observed. This result is consistent with most gene expression changes being caused by the stimulus of the Wnt pathway.

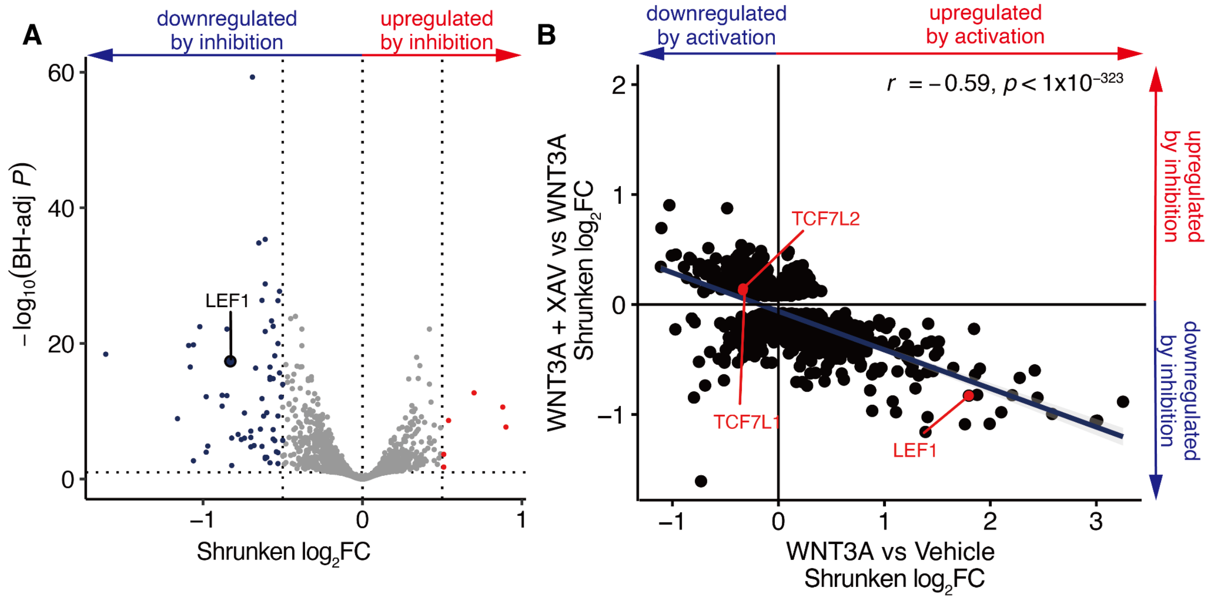

Supplementary Figure 10| ca/eQTL effect sizes compared to previous data

We compared the effect size of ca-QTL (**A**) and eQTL (**B**) in vehicle condition to previous studies using the same hNPC cell lines with slightly larger sample size (*10*, *11*). Strongly correlated effect sizes for unstimulated ca/eQTLs were found between the two datasets. In part **A**, the current study beta values are derived using RASQUAL as a scaled allelic ratio ranging from -0.5 to 0.5, which is not on the same scale as the beta values from Liang et al., which are calculated using a linear mixed effect model. One SNP showed opposite effect directionality across the caQTL datasets between the current and previous datasets (rs2076179). We suspect this was due to an error in allele assignment for that SNP in the previous analysis caused by sampling differences incorrectly changing the labeled minor allele for a SNP with allele frequency close to 0.50. We corrected this error for the one SNP prior to plotting.

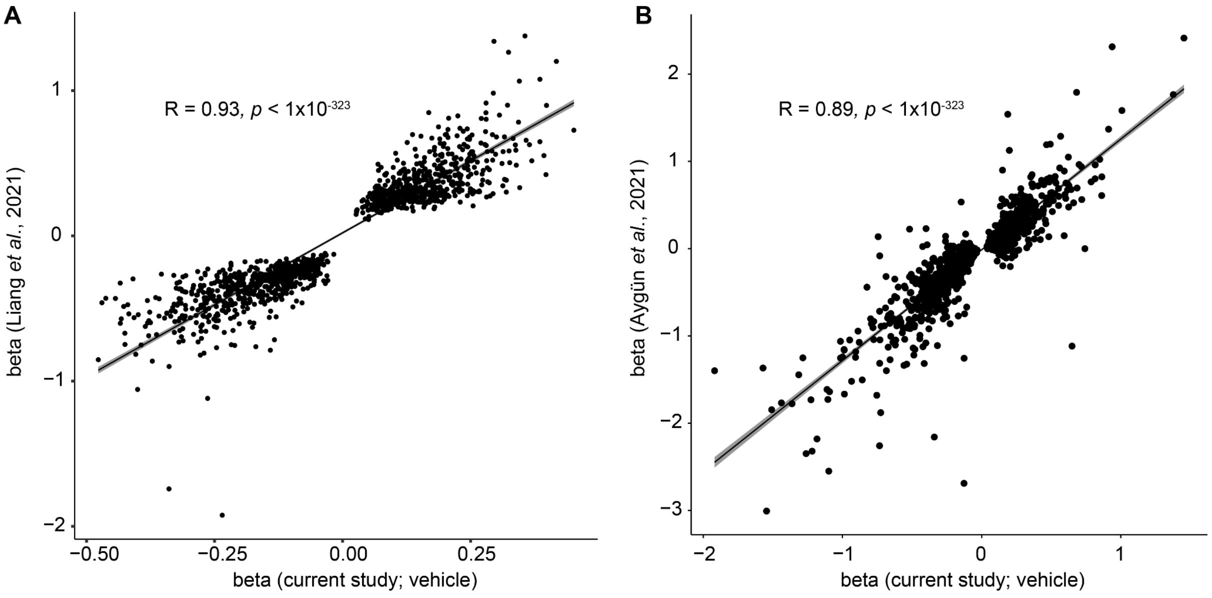

Supplementary Figure 11| eQTL-caQTL overlaps

Overlaps between caQTLs and eQTLs were called where a caSNP was within 1Mb of an eSNP with an LD $r^2 \geq 0.8$. The percentage of eQTLs (**A**) and caQTLs (**B**) overlapped within vehicle and WNT stimulus conditions are shown. The effect size of caQTL-eQTL sites which shared the same SNP position were compared within vehicle (**C**) and WNT stimulus conditions (WNT3A and CHIR shown in **D** and **E** respectively). SNPs selected to influence chromatin accessibility had relatively little overlap with those influencing gene expression likely because genetic variation affecting chromatin accessibility often does not always lead to changes in gene expression (about <2% of caQTLs had a shared eQTL based on LD overlap (r^2 >= 0.8). However, SNPs selected to influence gene expression more often also influence chromatin accessibility in that ~27% of eQTLs have a shared caQTL within condition, a comparable number observed in our previous study (34.9% caQTL-eQTLs overlap (*10*)).

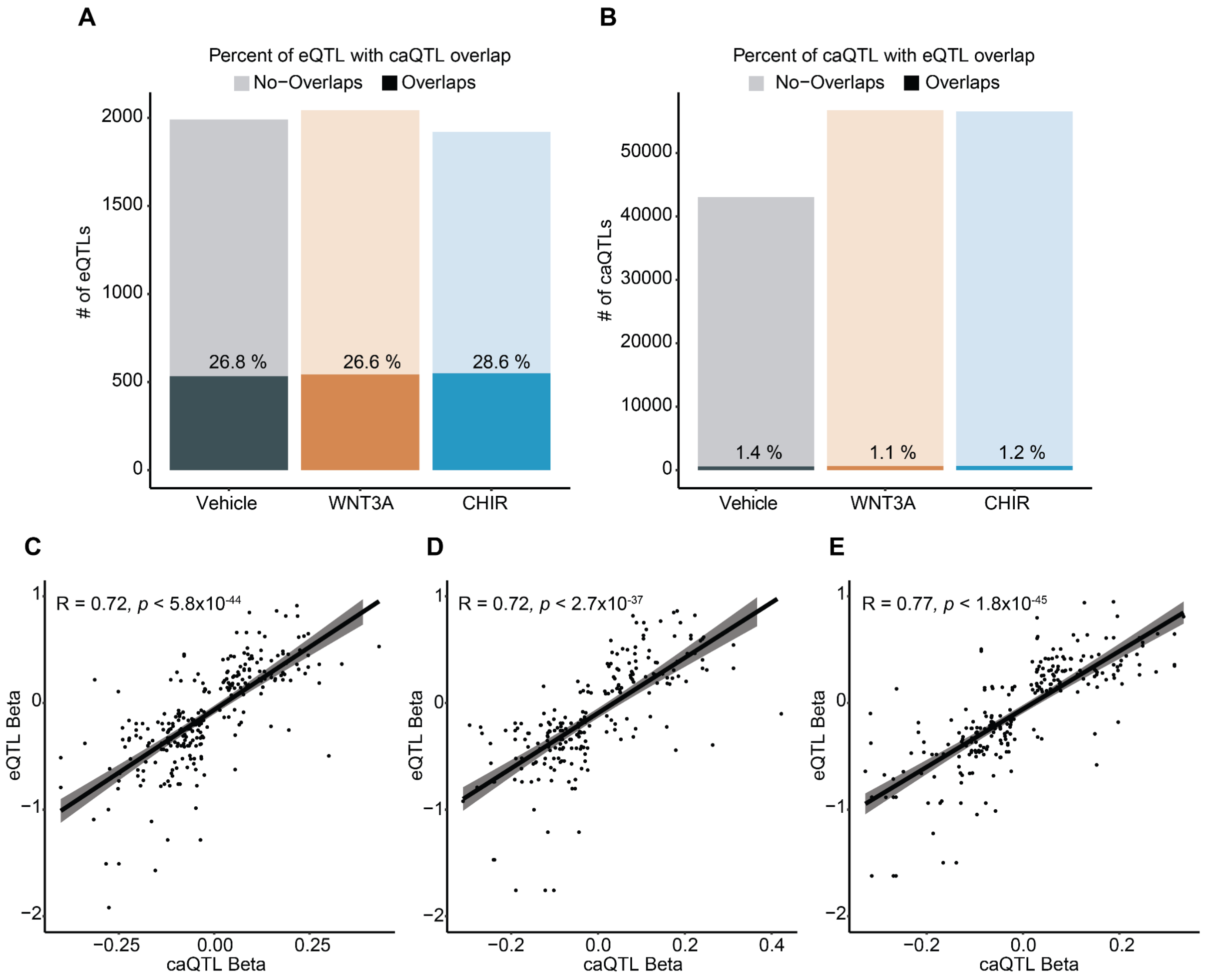

Supplementary Figure 12| Effect size differences across conditions

We compared effect sizes of index caQTLs (**A-B**) / eQTLs (**C-D**) in stimulated conditions versus vehicle conditions (WNT3A or CHIR in (**A,C**) and (**B,D**), respectively). Dots in red indicate significant interaction effects were observed (r-QTLs). Error bars are standard errors of beta. We observed lower correlation in r-QTLs as compared to non-r-QTLs. We note that some SNPs are not tested for a baseline model due to low expression thus no beta value exists.

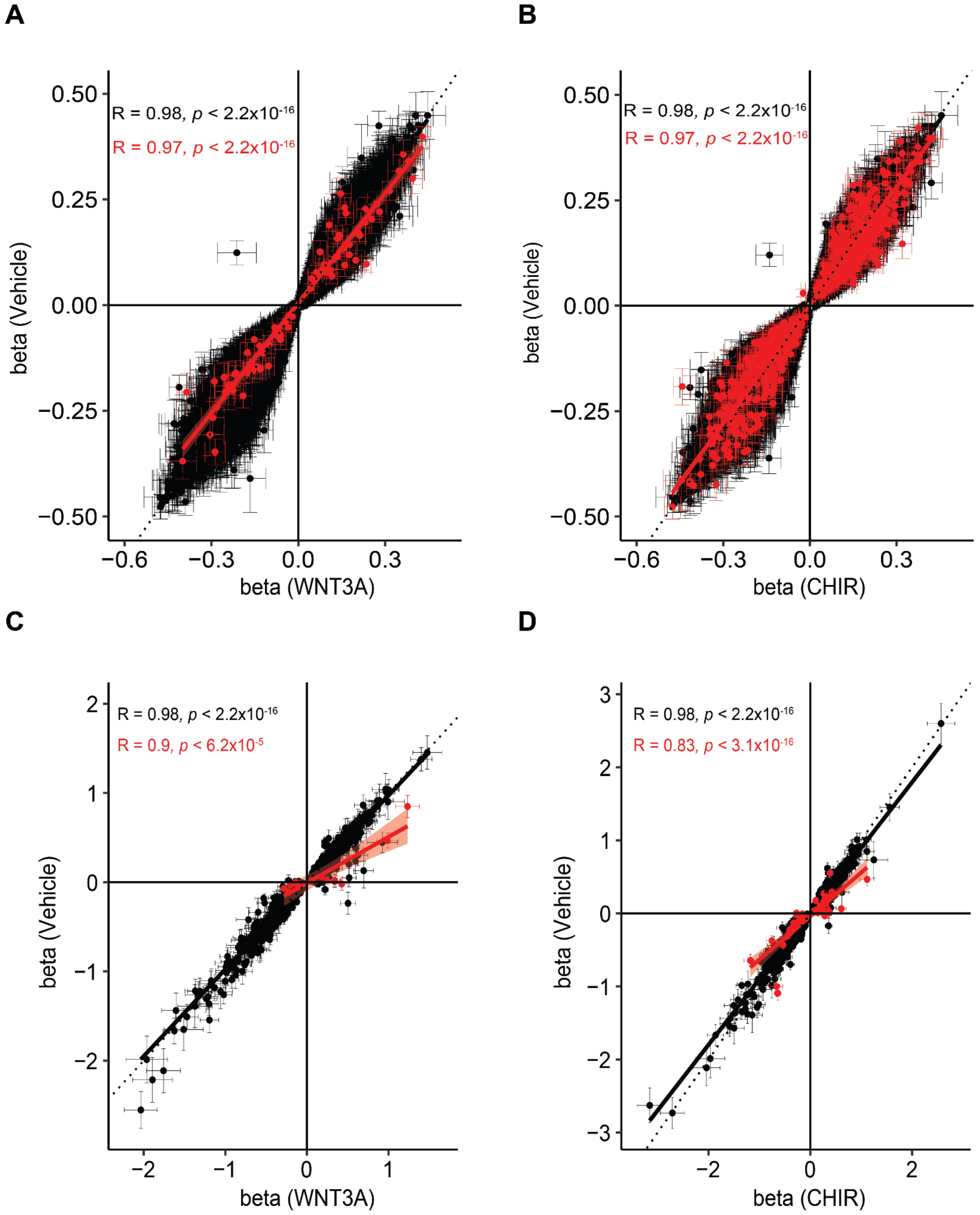

Supplementary Figure 13| Colocalization of ANKRD44 r-eQTL, schizophrenia, and the volume of a hippocampal subfield (presubiculum body)

(**A**) Boxplot showing increased ANKRD44 gene expression by rs979020-T and the SNP a significant interaction effect. (**B**) P-P plot from schizophrenia GWAS vs eQTL colored by r^2 in this study population to rs979020, providing evidence for a colocalization. (**C**) Conditional analysis of schizophrenia GWAS was performed using GCTA-cojo tool with LD from the UKBB reference panel (White British). GWAS *P* value (upper panel) and post-conditional analysis *P* value (bottom panel) are shown. An absence of GWAS signal after conditioning on the r-eQTL index provides evidence for colocalization. (**D**, **E**) Similar to (**B**, **C**) but using GWAS for volume of a hippocampal subfield (presubiculum body; left hemisphere).

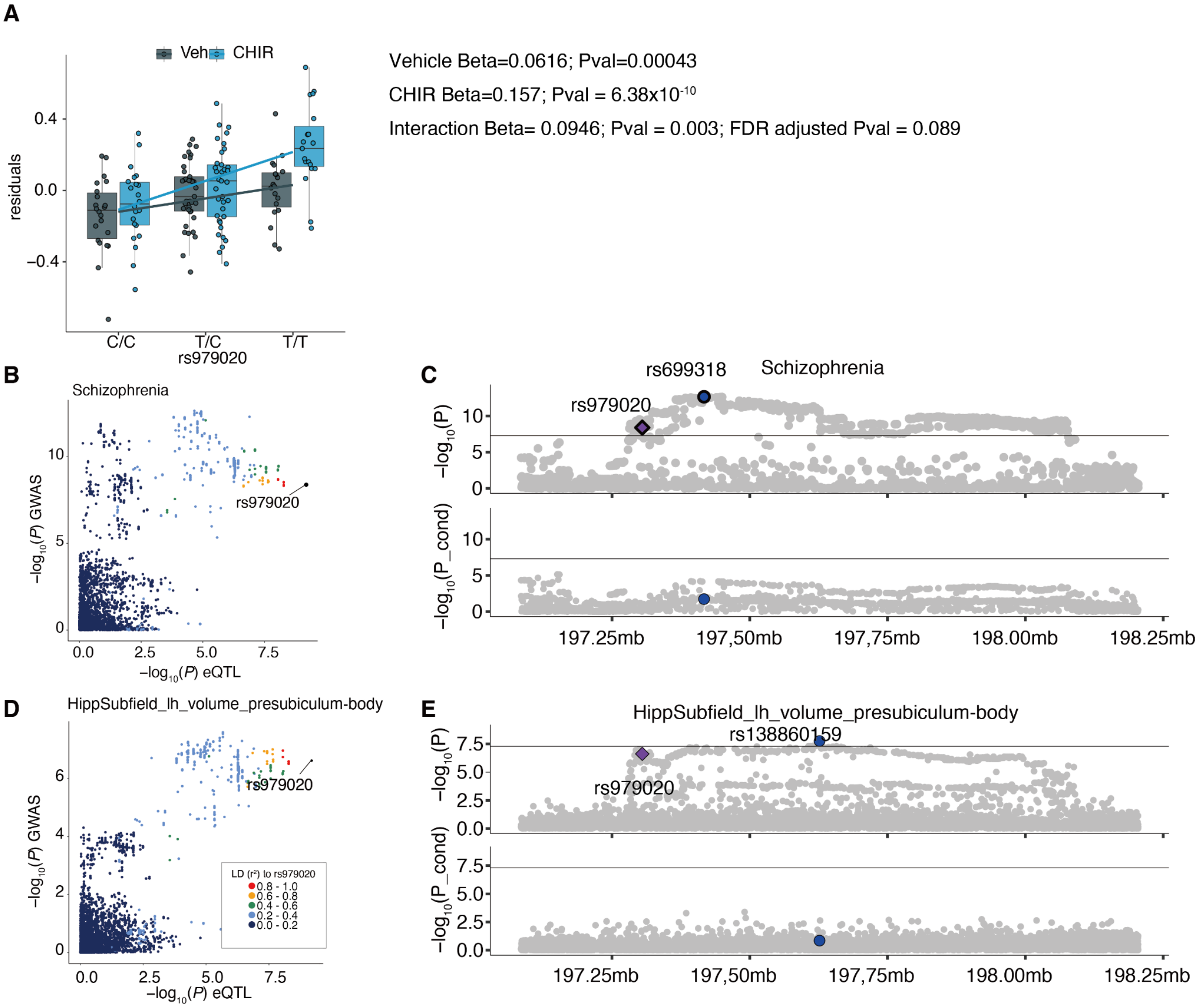

Supplementary Figure 14| Colocalization of an r-caQTL in DPYSL5 region and average thickness of the isthmus Cingulate

(**A**) We detected an r-caQTL at rs1992311 (FDR-adjusted *P* value = 0.04). We present boxplots showing differential genetic effects on chromatin accessibility in stimulated versus vehicle conditions in a linked SNP within the peak that disrupts a motif (rs4665363-G). (**B**) P-P plot for isthmus Cingulate GWAS (*2*) vs caQTL colored by r^2 in our population to rs1992311, providing evidence for colocalization. (**C**) Boxplot showing decreased *DPYSL5* expression by rs4665363-G. (**D**) Conditional analysis on GWAS for average thickness of Isthmus Cingulate was performed using the UKBB reference panel (White British). GWAS *P* value (upper panel) and post-conditional analysis *P* value (bottom panel) are shown. GCTA-cojo identified collinearity between rs4665363 and rs3820823 (r^2>0.9) thus both SNPs are not shown in the bottom panel. A decrease in GWAS signal after conditioning on the r-caQTL provides evidence for colocalization. (**E**) Logo plot predicting disruption of the Pou5f1::Sox2 motif by rs4665363-G. (**F**) Coverage plots of the peak showing the location of rs4665363 and a TCF/LEF motif present in the peak.

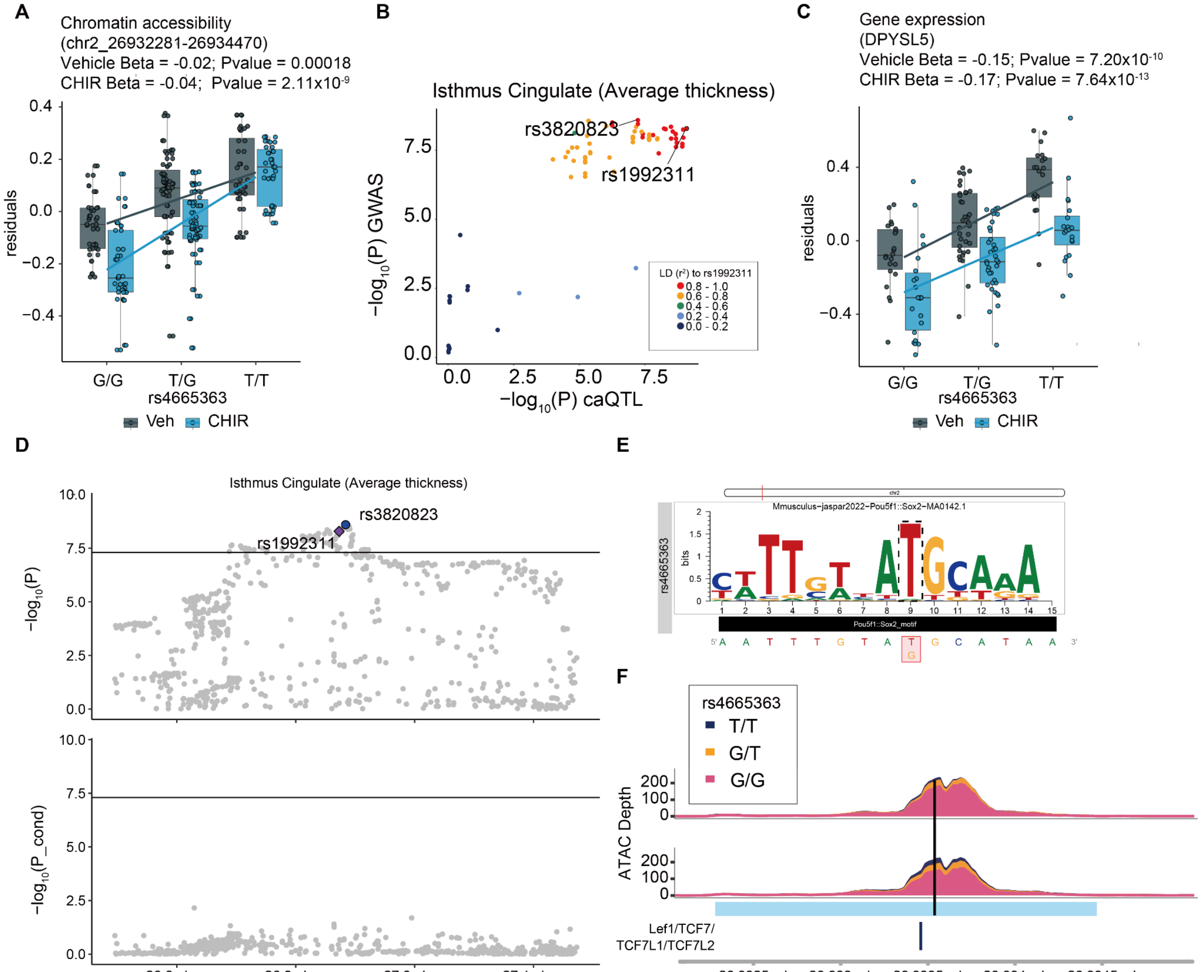

Supplementary Figure 15| Shared and context-specific QTL GWAS overlaps confirmed by eCAVIAR

Colocalization of eQTLs/caQTLs and brain-related GWAS phenotypes tested by eCAVIAR. (**A**) A cumulative number of colocalized eGenes/caPeaks after colocalization analysis by eCAVIAR and (**B**) shared/distinct colocalization in each condition. The use of stimulated conditions increased the number of brain-trait associated genes by 75.6% and peaks by 77.0%.

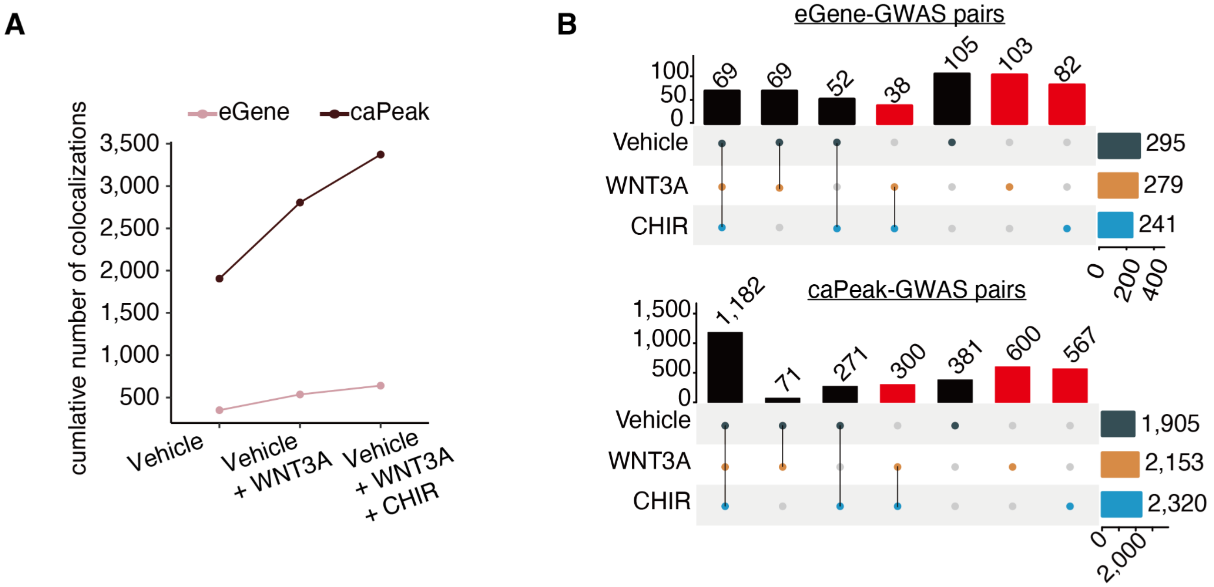

Supplementary Figure 16| Stimulus-specific GWAS colocalization of *FADS3* and Bipolar disorder

(**A**) GWAS and QTL locus plots in the region of*FADS3*. GWAS index SNP rs174592-A (protective allele) of bipolar disorder (BP) is in high LD with index caQTL (rs99780, r^2 = 0.86 in EUR, 0.85 in this study population) for RE (chr11:61850491-61851800) and *FAD3* index eQTL (rs174583, r^2 = 0.89 in EUR, r^2 = 0.78 in this study population). Rs174592-A is located within the peak and predicted to disrupt a STAT1::STAT2 transcription factor binding site motif. (**B**) Rs174592-A decreases chromatin accessibility of this WRE (Beta = -0.10; P = 6.81 x 10^{-13}, left), and expression of *FADS3* (Beta = -0.13 P = 2.95 x 10^{-7}, right) in CHIR condition. (**C**) Differential chromatin accessibility and *FADS3* gene expression between CHIR and Vehicle. (**D**) VST normalized expression counts of STAT1 and STAT2 are shown. Shrunken LFC and FDR-adjusted P values were estimated for 78 pairs. In the boxplots, chromatin accessibility or gene expression are colored by condition (gray: vehicle, blue:CHIR). (**E**) Schematic of FADS3 regulation through STAT1::STAT2 binding. Our data suggests characterization of the colocalized putative BP risk SNP (rs174592) as a functional variant regulating *FADS3* expression through differences in STAT1::STAT2 binding, which is inhibited during Wnt stimulation.

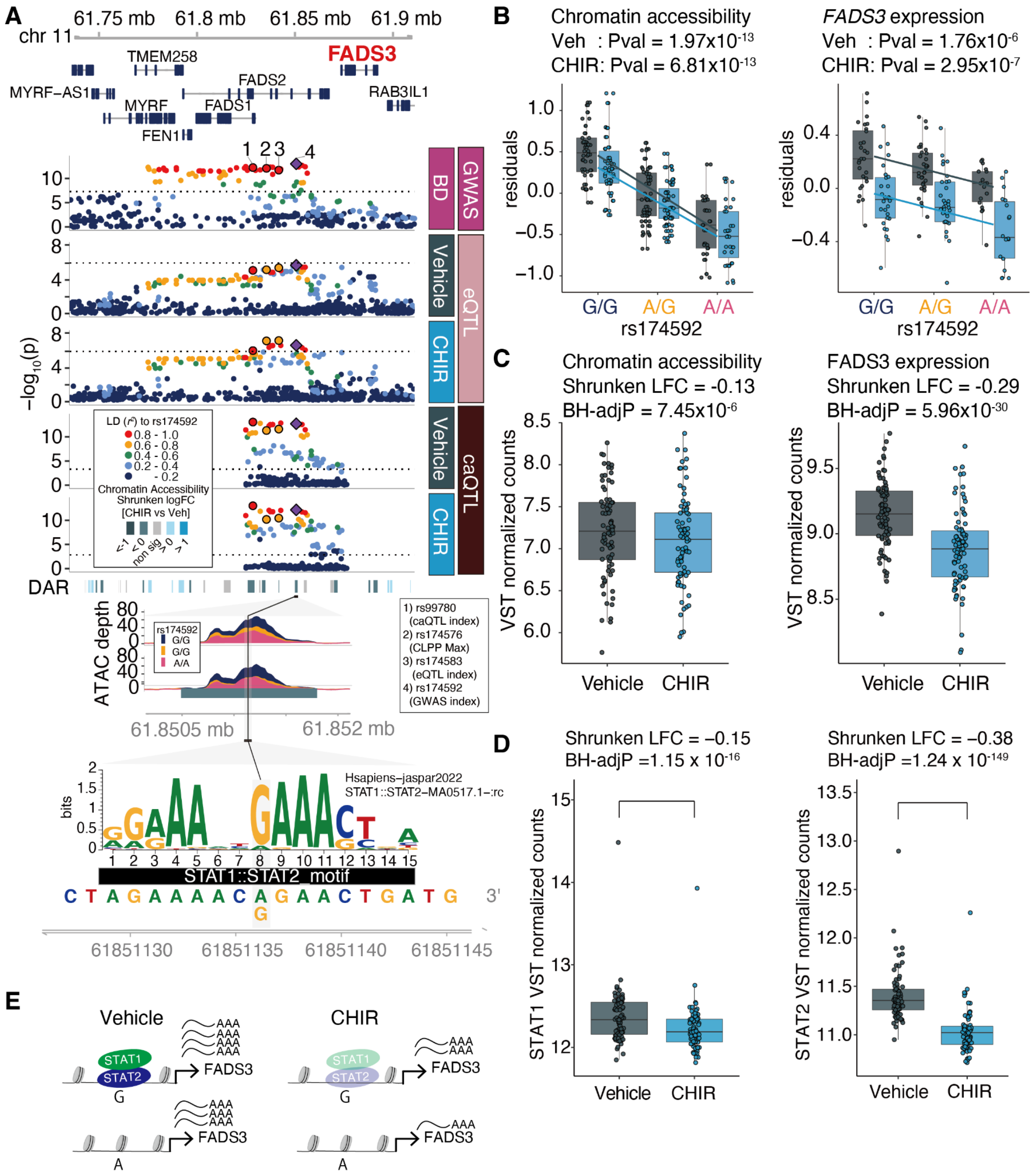
A
chr 11
61.75 mb 61.8 mb 61.85 mb 61.9 mb
TMEM258
FADS3
FADS2
MYRF−AS1
RAB3IL1
MYRF
FADS1
FEN1
BD GWAS
Vehicle eQTL
CHIR
Vehicle caQTL
CHIR
−log10(p)
LD (r²) to rs174592
0.8 - 1.0
0.6 - 0.8
0.4 - 0.6
0.2 - 0.4
- 0.2
Chromatin Accessibility Shrunken logFC [CHIR vs Veh]
<-1 <0 non sig >0 >1
DAR
ATAC depth
rs174592
G/G
G/G
A/A
1) rs99780 (caQTL index)
2) rs174576 (CLPP Max)
3) rs174583 (eQTL index)
4) rs174592 (GWAS index)
61.8505 mb
61.852 mb
bits
Hsapiens−jaspar2022
STAT1::STAT2−MA0517.1−:rc
STAT1::STAT2_motif
C T A G A A A A C A G A A C T G A T G 3'
G
61851130 61851135 61851140 61851145
B
Chromatin accessibility
Veh : Pval = 1.97x10-13
CHIR: Pval = 6.81x10-13
FADS3 expression
Veh : Pval = 1.76x10-6
CHIR: Pval = 2.95x10-7
residuals
G/G A/G A/A
rs174592
C
Chromatin accessibility
Shrunken LFC = -0.13
BH-adjP = 7.45x10-6
FADS3 expression
Shrunken LFC = -0.29
BH-adjP = 5.96x10-30
VST normalized counts
Vehicle CHIR
D
Shrunken LFC = −0.15
BH-adjP =1.15 x 10-16
Shrunken LFC = −0.38
BH-adjP =1.24 x 10-149
STAT1 VST normalized counts
STAT2 VST normalized counts
Vehicle CHIR
E
Vehicle
CHIR
STAT1
STAT2
FADS3
AAA
G
A

Supplementary Figure 17| Stimulus-specific *ENO4* eQTL colocalizing with regional cortical surface area GWAS

(**A**) Chromatin accessibility (chr10:116973711-116975130; left) and *ENO4* gene expression regulated by rs11197861. eQTL of *ENO4* was detected only in the stimulated condition (**A**, **B**). The eQTL is colocalized with several brain-related traits including surface area of Insula (**B**, **C**). (**B**) *P* values from Insula surface area GWAS, eQTL, caQTL for vehicle, CHIR condition are shown respectively. ETV2:FOXI1 motif is disrupted by the rs11197861-T located in the peak, which may result in decreased chromatin accessibility and *ENO4* gene expression. (**C**) Z scores from regional cortical surface area (Grasby et al., 2020). In addition to Insula, surface area of precentral and precuneus are also associated with this SNP (indicated by asterisks; $p < 5 \times 10^{-8}$).

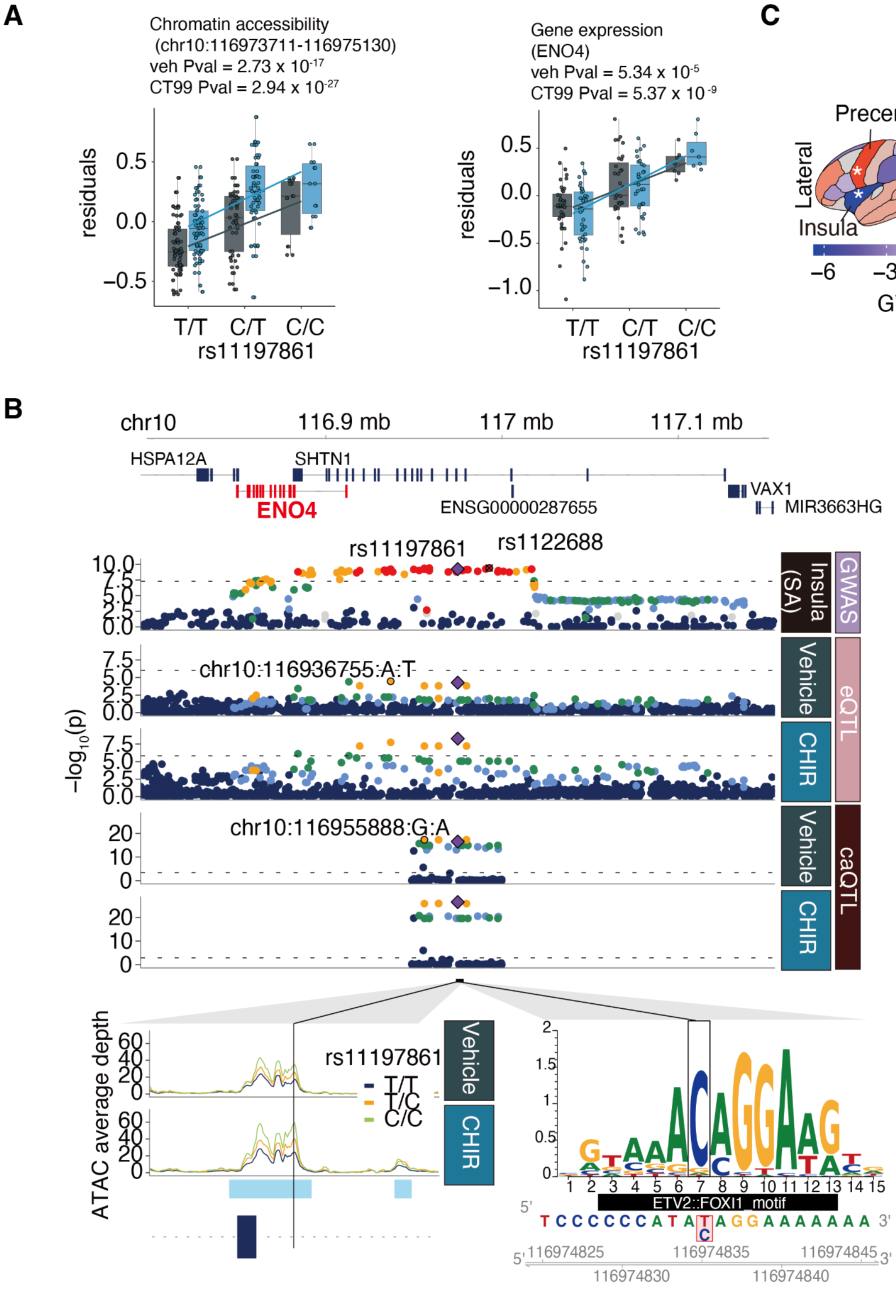

Supplementary Figure 18| Optimizing control for known and unknown technical confounding in eQTL

We tested different numbers of PC variables to determine the number of PCs to include for correcting expression values prior to running limix_qtl. (**A**) The plot shows the number of discovered eGenes with respect to the number of PCs at FDR-adjusted P < 0.1. and the number of overlapping found eGenes across models (**B-D**). To identify independent eQTLs, we repeated eQTL mapping by including the index eSNP in the association model until no SNP passed the genome-wide threshold (FDR-adjusted P < 0.1). In table (**E**), we show the number of SNPs discovered in each round of conditional associations.

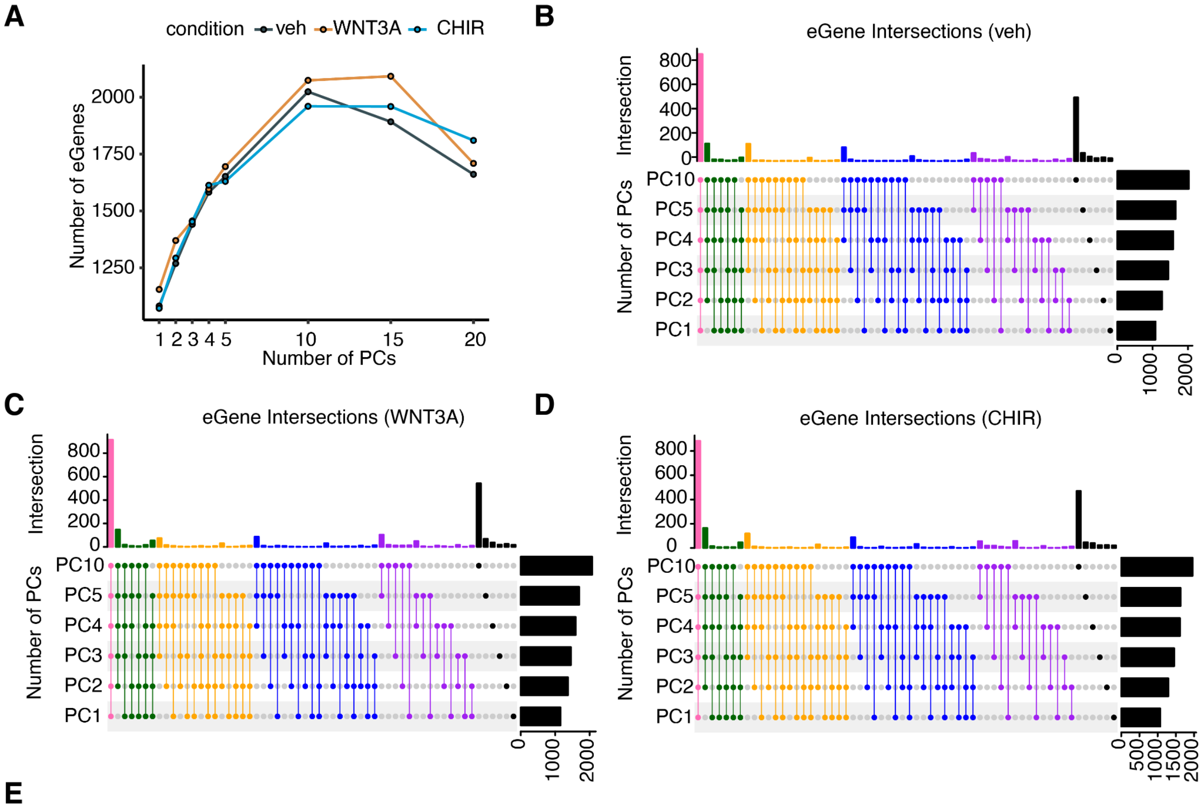

E

# of eSNPs per Gene		
**Round**	**# eGenes**	**condition**
1	2024	Vehicle
2	65	Vehicle
1	2074	Wnt3A
2	72	Wnt3A
3	1	Wnt3A
1	1960	CHIR
2	65	CHIR
3	1	CHIR

Supplementary Tables

S1 - S4, S6-S17 are provided in Excel files.

Supplementary Table 1| Differentially accessible peaks

A. WNT3A vs Vehicle; B. CHIR vs Vehicle; C. WNT3A+XAV vs WNT3A

Column Name	Description
Chromosome	chromosome
start	peak start position (hg38)
end	peak start position (hg38)
baseMean	average of the normalized count values
log2FoldChange	shrunken log2Fold Change
lfsSE	standard error of Shrunken log2Fold Change
pvalue	p value
padj	BH-adjusted Pvalue

Supplementary Table 2| TF motif enrichment analysis

A. WNT3A vs Vehicle; B. CHIR vs Vehicle

Column Name	Description
TFname	TF name
TFID	TF ID
estimate	coefficient from logistic regression
estimate_err	coefficient standard error from logistic regression
z	z score = estimate/estimate_err
pval	pvalue from logistic regression
padj	BH-adjsted Pvalue

Supplementary Table 3| Differentially expressed genes

A. WNT3A vs Vehicle; B. CHIR vs Vehicle; C. WNT3A+XAV vs WNT3A

Column Name	Description
gene_id	Ensembl ID
gene_name	HGNC gene name
gene_type	protein_coding/lncRNA
chromosome	chromosome
start	collapsed gene start position
end	collapsed gene end position
strand	strand
baseMean	average of the normalized count values
log2FoldChange	shrunken log2Fold Change
lfcSE	standard error of Shrunken log2Fold Change
pvalue	p value
padj	BH-adjusted Pvalue

Supplementary Table 4| Pathway enrichment analysis

A. WNT3A vs Vehicle; B. CHIR vs Vehicle
The details can be found on the gprofiler website (https://biit.cs.ut.ee/gprofiler/page/apis)

**Column Name**	**Description**
query	DEG direction (up-regulated/down-regulated/all)
source	KEGG / REAC(REACTOME)
term_id	pathway ID
term_name	pathway name
p_value	Hypergeometric p-value after correction for multiple testing (BH)
query_size	number of genes in the query
intersection_size	number of genes in the query annotated to the corresponding term
term_size	number of genes annotated to the term

effective_domain_size	total number of genes in the background that is used for Hypergeometric test
GeneRatio	interaction_size/query_size

Supplementary Table 5| Summary of peak-gene correlation

Condition	Pearson's Correlation Coefficient			% of positively correlated pairs
	max r	mean(abs.r)	min r	
Vehicle	0.95	0.50	-0.78	80.4%
WNT3A	0.95	0.53	-0.83	83.4%
CHIR	0.97	0.50	-0.75	83.6%

Supplementary Table 6| Peak-gene pairs

A. WNT3A vs Vehicle; B. CHIR vs Vehicle

**Column Name**	**Description**
chromosome	chromosome position of peak
start	start position of peak
end	end position of peak
gene_id	Ensembl ID
gene_name	HGNC gene name
distance	distance from TSS to center of peak
coefficient	coefficient in a linear model
fdr	BH-adjusted P value
(interaction coefficient)	Interaction coefficient in a linear model (WNT3A/CHIR vs Vehicle)
(fdr_interact)	BH-adjusted P value for interaction test (WNT3A/CHIR vs Vehicle)

Supplementary Table 7| Disease enrichment analysis results(DEG)

Details can be found in DisGeNet (https://www.disgenet.org/home/).

Column Name	Description
status	DEG category (all/up/down/nonsig)
cond	condition (WNT3A/CHIR/nonsig)
ID	phenotype ID
Description	phenotype description
source	“CURATED”
BgRatio	number of genes associated to the disease and number of background gene in database
pvalue	Fisher's exact test P value
FDR	BH-adjusted P value
disease_class	MESH tree branch class
disease_class_name	MESH tree branch name
disease_semantic_type	UMLS® semantic types
shared_symbol	shared gene symbol associated to the disease

Supplementary Table 8| Summary of GWASs used in this study

Column Name	Description
GWAS Name	GWAS phenotype
Ref	Reference

Supplementary Table 9| Partitioned heritability analysis results

Column Name	Description
Annotation type	Tissue / Cell / WNT stimulation
Data set	WREs used for LDSC
GWAS phenotype	GWAS phenotype
Coefficient	coefficient from LDSC model
Coefficient stderr	standard error of Coefficient
Coefficient P	One-sided Pvalue from Coefficient Z score
BH-adjusted P	BH-adjusted Pvalue

Supplementary Table 10| List of caQTL - caPeak

Column Name	Description
Feature	peak ID
rs.ID	SNP ID
Chromosome	chromosome position of peak/snp
SNP.Position	SNP position
Ref.Allele	reference allele
Alt.Alele	alternative allele (the effect allele)
Allele.Frequency	allele frequency of reference allele
HWE.Chi_Square.Statistic	Hardy-Weinberg chi-square statistic
Imputation.Quality..IA.	imputation quality score
Log_10.Benjamini.Hochberg.Q.value	$\log_{10}$Benjamini-Hochberg q-value
Chi.square.statistic..2.x.log.Likelihood.ratio.	chi square statistic (2 x log-likelihood ratio)
Effect.Size	cis-regulatory effect parameter (pi)

Sequencing.mapping.error.rate..Delta.	sequencing/mapping error rate (Delta)
Reference.allele.mapping.bias..Phi.	reference allele mapping bias (Phi)
Overdispersion	overdispersion estimate
SNP.ID.within.the.region	ID of snp within tested region
No..of.feature.SNPs	total number of snps tested within the feature
No..of.tested.SNPs	total number of snps tested within cis window
No..of.iterations.for.null.hypothesis	number of iterations for null hypothesis
No..of.iterations.for.alternative.hypothesis	number of iterations for alternative hypothesis
Random.location.of.ties	location of snp used as lead if tie occurs
Log.likelihood.of.the.null.hypothesis	log-likelihood of the null hypothesis
Convergence.status	job outputs status (0=success)
Squared.correlation.between.prior.and.posterior.genotypes..fSNPs.	squared correlation between prior and posterior genotypes (fSNPs; feature snps)
Squared.correlation.between.prior.and.posterior.genotypes..rSNP.	squared correlation between prior and posterior genotypes (rSNP; single cis-regulatory snp)
PValue	p value
Distance_from_PeakCenter	distance of tested snp from the center of the peak

Supplementary Table 11| List of eQTL - eGene

**Column Name**	**Description**
gene_id	Ensembl ID
gene_chr	chromosome position of gene
gene_start	start position of gene

gene_end	end position of gene
gene_name	HGNC gene name
feature_strand	strand (+/-)
snp_id	snp ID
snp_chr	chromosome position of SNP
snp_pos	SNP position
Assessed Allele	Assessed (also called effect) allele
maf	minor allele frequency
beta	beta estimate of main effect
beta_se	standard error of beta estimate
empirical_feature_p_value	permutation p value
p_value	p value
global_adj_p_value	BH-adjusted P value
iteration	iteration (1 = primary)

Supplementary Table 12| caSNP TF motif disruption (in peak)

**Column Name**	**Description**
Chr	chromosome position
snpPos	snp position
strand	strand (+/-)
SNP_id	SNP ID
REF	reference allele
ALT	alternative allele
geneSymbol	motif gene symbol
dataSource	motif data source
providerId	JASPAR matrix ID

effect	disruption effect strength
motifPosition_start	start position of disrupted motif
motifPosition_end	end position of disrupted motif

Supplementary Table 13| List of response-caQTLs

**Column Name**	**Description**
Feature	peak ID
SNP	snp ID
PValue	p value
Interaction_Beta	interaction effect
Chr	chromosome position
Position	SNP position
global	BH-adjusted Pvalue
Peak_SNP	combined peak ID and snp ID
Ref	reference allele
Alt	alternative allele
RASQUAL_PValue_Veh	p value from vehicle RASQUAL output
RASQUAL_PValue_Condition	p value from condition RASQUAL output
Pi_Veh	cis-regulatory effect parameter (pi) from vehicle RASQUAL output
EffectSize_Veh	beta estimate of snp effect in vehicle
Pi_Condition	cis-regulatory effect parameter (pi) from condition RASQUAL output
EffectSize_Condition	beta estimate of snp effect in condition

Supplementary Table 14| List of response-eQTLs

A. WNT3A vs Vehicle, B. CHIR vs Vehicle

Column Name	Description
gene_name	HGNC gene name
chromosome	chromosome position
start	start position of gene
end	end position of gene
snp_id	SNP ID
Assessed Allele	assessed allele
maf	minor allele (assessed allele) frequency
beta	interaction effect
beta_se	standard error of interaction effect
p_value	P value of interaction effect
padj	BH-adjusted Pvalue

Supplementary Table 15| caQTL-eQTL colocalizations

Column Name	Description
gene_chr	chromosome position (eQTL)
gene_start	start position of gene
gene_end	end position of gene
gene_name	HGNC gene name
snp_id	SNP ID (eQTL)
Assessed Allele	assessed allele (eQTL)
maf	minor allele (assessed allele) frequency (eQTL)
beta	interaction effect (eQTL)
beta_se	standard error of interaction effect (eQTL)

p_value	P value of interaction effect (eQTL)
global_adj_p_value	BH-adjusted Pvalue (eQTL)
Feature	peak ID
rs.ID	SNP ID (caQTL)
Chromosome	chromosome position (caQTL)
SNP.Position	SNP position (caQTL)
Ref.Allele	reference allele (caQTL)
Alt.allele	alternative allele (caQTL)
Effect.Size	cis-regulatory effect parameter (pi)
PValue	p value (caQTL)
Distance_from_PeakCenter	distance of tested SNP from the center of the peak
R2	*r2* between eQTL and caQTL SNP

Supplementary Table 16| GWAS colocalization results (caQTL)

**Column Name**	**Description**
chr	chromosome
Peak	peak ID
Wnt_Pos	caQTL index position
Wnt_SNP	caQTL SNP ID
GWAS_Pos	GWAS index Position
GWAS_SNP	GWAS SNP ID
R2_CurrentStudy	r^2 between GWAS index SNP and eQTL index SNP estimated in this study population
R2_1kgEUR	r^2 between GWAS index SNP and eQTL index SNP estimated in 1KG EUR population
GWASID	GWAS phenotype(s)

Supplementary Table 17| GWAS colocalization results (eQTL)

Column Name	Description
condition	stimulus condition (veh/WNT3A/CHIR)
gene	HGNC gene name
chr	chromosome
WntPos	eQTL index position
GWAS Pos	GWAS index Position
r2_current_study	r^2 between GWAS index SNP and eQTL index SNP estimated in this study population
r2_1kgEUR	r^2 between GWAS index SNP and eQTL index SNP estimated in 1KG EUR population

References continued

51. M. J. Roeske, C. Konradi, S. Heckers, A. S. Lewis, Hippocampal volume and hippocampal neuron density, number and size in schizophrenia: a systematic review and meta-analysis of postmortem studies. *Mol. Psychiatry*. **26**, 3524–3535 (2021).

52. D. Sasabayashi, R. Yoshimura, T. Takahashi, Y. Takayanagi, S. Nishiyama, Y. Higuchi, Y. Mizukami, A. Furuichi, M. Kido, M. Nakamura, K. Noguchi, M. Suzuki, Reduced Hippocampal Subfield Volume in Schizophrenia and Clinical High-Risk State for Psychosis. *Front. Psychiatry*. **12**, 642048 (2021).

53. A. Veyrac, S. Reibel, J. Sacquet, M. Mutin, J.-P. Camdessanche, P. Kolattukudy, J. Honnorat, F. Jourdan, CRMP5 regulates generation and survival of newborn neurons in olfactory and hippocampal neurogenic areas of the adult mouse brain. *PLoS One*. **6**, e23721 (2011).

54. N. Mullins, A. J. Forstner, K. S. O'Connell, B. Coombes, J. R. I. Coleman, Z. Qiao, T. D. Als, T. B. Bigdeli, S. Børte, J. Bryois, A. W. Charney, O. K. Drange, M. J. Gandal, S. P. Hagenaars, M. Ikeda, N. Kamitaki, M. Kim, K. Krebs, G. Panagiotaropoulou, B. M. Schilder, L. G. Sloofman, S. Steinberg, V. Trubetskoy, B. S. Winsvold, H.-H. Won, L. Abramova, K. Adorjan, E. Agerbo, M. Al Eissa, D. Albani, N. Alliey-Rodriguez, A. Anjorin, V. Antilla, A. Antoniou, S. Awasthi, J. H. Baek, M. Bækvad-Hansen, N. Bass, M. Bauer, E. C. Beins, S. E. Bergen, A. Birner, C. Bøcker Pedersen, E. Bøen, M. P. Boks, R. Bosch, M. Brum, B. M. Brumpton, N. Brunkhorst-Kanaan, M. Budde, J. Bybjerg-Grauholm, W. Byerley, M. Cairns, M. Casas, P. Cervantes, T.-K. Clarke, C. Cruceanu, A. Cuellar-Barboza, J. Cunningham, D. Curtis, P. M. Czerski, A. M. Dale, N. Dalkner, F. S. David, F. Degenhardt, S. Djurovic, A. L. Dobbyn, A. Douzenis, T. Elvsåshagen, V. Escott-Price, I. N. Ferrier, A. Fiorentino, T. M. Foroud, L. Forty, J. Frank, O. Frei, N. B. Freimer, L. Frisén, K. Gade, J. Garnham, J.

Gelernter, M. Giørtz Pedersen, I. R. Gizer, S. D. Gordon, K. Gordon-Smith, T. A. Greenwood, J. Grove, J. Guzman-Parra, K. Ha, M. Haraldsson, M. Hautzinger, U. Heilbronner, D. Hellgren, S. Herms, P. Hoffmann, P. A. Holmans, L. Huckins, S. Jamain, J. S. Johnson, J. L. Kalman, Y. Kamatani, J. L. Kennedy, S. Kittel-Schneider, J. A. Knowles, M. Kogevinas, M. Koromina, T. M. Kranz, H. R. Kranzler, M. Kubo, R. Kupka, S. A. Kushner, C. Lavebratt, J. Lawrence, M. Leber, H.-J. Lee, P. H. Lee, S. E. Levy, C. Lewis, C. Liao, S. Lucae, M. Lundberg, D. J. MacIntyre, S. H. Magnusson, W. Maier, A. Maihofer, D. Malaspina, E. Maratou, L. Martinsson, M. Mattheisen, S. A. McCarroll, N. W. McGregor, P. McGuffin, J. D. McKay, H. Medeiros, S. E. Medland, V. Millischer, G. W. Montgomery, J. L. Moran, D. W. Morris, T. W. Mühleisen, N. O'Brien, C. O'Donovan, L. M. Olde Loohuis, L. Oruc, S. Papiol, A. F. Pardiñas, A. Perry, A. Pfennig, E. Porichi, J. B. Potash, D. Quested, T. Raj, M. H. Rapaport, J. R. DePaulo, E. J. Regeer, J. P. Rice, F. Rivas, M. Rivera, J. Roth, P. Roussos, D. M. Ruderfer, C. Sánchez-Mora, E. C. Schulte, F. Senner, S. Sharp, P. D. Shilling, E. Sigurdsson, L. Sirignano, C. Slaney, O. B. Smeland, D. J. Smith, J. L. Sobell, C. Søholm Hansen, M. Soler Artigas, A. T. Spijker, D. J. Stein, J. S. Strauss, B. Świątkowska, C. Terao, T. E. Thorgeirsson, C. Toma, P. Tooney, E.-E. Tsermpini, M. P. Vawter, H. Vedder, J. T. R. Walters, S. H. Witt, S. Xi, W. Xu, J. M. K. Yang, A. H. Young, H. Young, P. P. Zandi, H. Zhou, L. Zillich, HUNT All-In Psychiatry, R. Adolfsson, I. Agartz, M. Alda, L. Alfredsson, G. Babadjanova, L. Backlund, B. T. Baune, F. Bellivier, S. Bengesser, W. H. Berrettini, D. H. R. Blackwood, M. Boehnke, A. D. Børglum, G. Breen, V. J. Carr, S. Catts, A. Corvin, N. Craddock, U. Dannlowski, D. Dikeos, T. Esko, B. Etain, P. Ferentinos, M. Frye, J. M. Fullerton, M. Gawlik, E. S. Gershon, F. S. Goes, M. J. Green, M. Grigoroiu-Serbanescu, J. Hauser, F. Henskens, J. Hillert, K. S. Hong, D. M. Hougaard, C. M. Hultman, K. Hveem, N. Iwata, A. V. Jablensky, I. Jones, L. A. Jones, R. S. Kahn, J. R. Kelsoe, G. Kirov, M. Landén, M. Leboyer, C. M. Lewis, Q. S. Li, J. Lissowska, C. Lochner, C. Loughland, N. G. Martin, C. A. Mathews, F. Mayoral, S. L. McElroy, A. M. McIntosh, F. J. McMahon, I. Melle, P. Michie, L. Milani, P. B. Mitchell, G. Morken, O. Mors, P. B. Mortensen, B. Mowry, B. Müller-Myhsok, R. M. Myers, B. M. Neale, C. M. Nievergelt, M. Nordentoft, M. M. Nöthen, M. C. O'Donovan, K. J. Oedegaard, T. Olsson, M. J. Owen, S. A. Paciga, C. Pantelis, C. Pato, M. T. Pato, G. P. Patrinos, R. H. Perlis, D. Posthuma, J. A. Ramos-Quiroga, A. Reif, E. Z. Reininghaus, M. Ribasés, M. Rietschel, S. Ripke, G. A. Rouleau, T. Saito, U. Schall, M. Schalling, P. R. Schofield, T. G. Schulze, L. J. Scott, R. J. Scott, A. Serretti, C. Shannon Weickert, J. W. Smoller, H. Stefansson, K. Stefansson, E. Stordal, F. Streit, P. F. Sullivan, G. Turecki, A. E. Vaaler, E. Vieta, J. B. Vincent, I. D. Waldman, T. W. Weickert, T. Werge, N. R. Wray, J.-A. Zwart, J. M. Biernacka, J. I. Nurnberger, S. Cichon, H. J. Edenberg, E. A. Stahl, A. McQuillin, A. Di Florio, R. A. Ophoff, O. A. Andreassen, Genome-wide association study of more than 40,000 bipolar disorder cases provides new insights into the underlying biology. *Nat. Genet.* **53**, 817–829 (2021).

55. T. Sun, R. F. Hevner, Growth and folding of the mammalian cerebral cortex: from molecules to malformations. *Nat. Rev. Neurosci.* **15**, 217–232 (2014).

56. G. L. Boulting, E. Durresi, B. Ataman, M. A. Sherman, K. Mei, D. A. Harmin, A. C. Carter, D. R. Hochbaum, A. J. Granger, J. M. Engreitz, S. Hrvatin, M. R. Blanchard, M. G. Yang, E. C. Griffith, M. E. Greenberg, Activity-dependent regulome of human GABAergic neurons reveals new patterns of gene regulation and neurological disease heritability. *Nat. Neurosci.* **24**, 437–448 (2021).

57. J. Jerber, D. D. Seaton, A. S. E. Cuomo, N. Kumasaka, J. Haldane, J. Steer, M. Patel, D. Pearce, M. Andersson, M. J. Bonder, E. Mountjoy, M. Ghoussaini, M. A. Lancaster, HipSci Consortium, J. C. Marioni, F. T. Merkle, D. J. Gaffney, O. Stegle, Population-scale

single-cell RNA-seq profiling across dopaminergic neuron differentiation. *Nat. Genet.* **53**, 304–312 (2021).

58. J. M. Wolter, B. D. Le, N. Matoba, M. J. Lafferty, N. Aygün, D. Liang, K. Courtney, J. Song, J. Piven, M. J. Zylka, J. L. Stein, Cellular genome-wide association study identifies common genetic variation influencing lithium induced neural progenitor proliferation. *Biol. Psychiatry* (2022), doi:10.1016/j.biopsych.2022.08.014.

59. M. B. Major, N. D. Camp, J. D. Berndt, X. Yi, S. J. Goldenberg, C. Hubbert, T. L. Biechele, A.-C. Gingras, N. Zheng, M. J. Maccoss, S. Angers, R. T. Moon, Wilms tumor suppressor WTX negatively regulates WNT/beta-catenin signaling. *Science*. **316**, 1043–1046 (2007).

60. M. Malek, M. J. Taghiyar, L. Chong, G. Finak, R. Gottardo, R. R. Brinkman, flowDensity: reproducing manual gating of flow cytometry data by automated density-based cell population identification. *Bioinformatics*. **31**, 606–607 (2015).

61. M. R. Corces, A. E. Trevino, E. G. Hamilton, P. G. Greenside, N. A. Sinnott-Armstrong, S. Vesuna, A. T. Satpathy, A. J. Rubin, K. S. Montine, B. Wu, A. Kathiria, S. W. Cho, M. R. Mumbach, A. C. Carter, M. Kasowski, L. A. Orloff, V. I. Risca, A. Kundaje, P. A. Khavari, T. J. Montine, W. J. Greenleaf, H. Y. Chang, An improved ATAC-seq protocol reduces background and enables interrogation of frozen tissues. *Nat. Methods*. **14**, 959–962 (2017).

62. D. Taliun, D. N. Harris, M. D. Kessler, J. Carlson, Z. A. Szpiech, R. Torres, S. A. G. Taliun, A. Corvelo, S. M. Gogarten, H. M. Kang, A. N. Pitsillides, J. LeFaive, S.-B. Lee, X. Tian, B. L. Browning, S. Das, A.-K. Emde, W. E. Clarke, D. P. Loesch, A. C. Shetty, T. W. Blackwell, A. V. Smith, Q. Wong, X. Liu, M. P. Conomos, D. M. Bobo, F. Aguet, C. Albert, A. Alonso, K. G. Ardlie, D. E. Arking, S. Aslibekyan, P. L. Auer, J. Barnard, R. G. Barr, L. Barwick, L. C. Becker, R. L. Beer, E. J. Benjamin, L. F. Bielak, J. Blangero, M. Boehnke, D. W. Bowden, J. A. Brody, E. G. Burchard, B. E. Cade, J. F. Casella, B. Chalazan, D. I. Chasman, Y.-D. I. Chen, M. H. Cho, S. H. Choi, M. K. Chung, C. B. Clish, A. Correa, J. E. Curran, B. Custer, D. Darbar, M. Daya, M. de Andrade, D. L. DeMeo, S. K. Dutcher, P. T. Ellinor, L. S. Emery, C. Eng, D. Fatkin, T. Fingerlin, L. Forer, M. Fornage, N. Franceschini, C. Fuchsberger, S. M. Fullerton, S. Germer, M. T. Gladwin, D. J. Gottlieb, X. Guo, M. E. Hall, J. He, N. L. Heard-Costa, S. R. Heckbert, M. R. Irvin, J. M. Johnsen, A. D. Johnson, R. Kaplan, S. L. R. Kardia, T. Kelly, S. Kelly, E. E. Kenny, D. P. Kiel, R. Klemmer, B. A. Konkle, C. Kooperberg, A. Köttgen, L. A. Lange, J. Lasky-Su, D. Levy, X. Lin, K.-H. Lin, C. Liu, R. J. F. Loos, L. Garman, R. Gerszten, S. A. Lubitz, K. L. Lunetta, A. C. Y. Mak, A. Manichaikul, A. K. Manning, R. A. Mathias, D. D. McManus, S. T. McGarvey, J. B. Meigs, D. A. Meyers, J. L. Mikulla, M. A. Minear, B. D. Mitchell, S. Mohanty, M. E. Montasser, C. Montgomery, A. C. Morrison, J. M. Murabito, A. Natale, P. Natarajan, S. C. Nelson, K. E. North, J. R. O'Connell, N. D. Palmer, N. Pankratz, G. M. Peloso, P. A. Peyser, J. Pleiness, W. S. Post, B. M. Psaty, D. C. Rao, S. Redline, A. P. Reiner, D. Roden, J. I. Rotter, I. Ruczinski, C. Sarnowski, S. Schoenherr, D. A. Schwartz, J.-S. Seo, S. Seshadri, V. A. Sheehan, W. H. Sheu, M. B. Shoemaker, N. L. Smith, J. A. Smith, N. Sotoodehnia, A. M. Stilp, W. Tang, K. D. Taylor, M. Telen, T. A. Thornton, R. P. Tracy, D. J. Van Den Berg, R. S. Vasan, K. A. Viaud-Martinez, S. Vrieze, D. E. Weeks, B. S. Weir, S. T. Weiss, L.-C. Weng, C. J. Willer, Y. Zhang, X. Zhao, D. K. Arnett, A. E. Ashley-Koch, K. C. Barnes, E. Boerwinkle, S. Gabriel, R. Gibbs, K. M. Rice, S. S. Rich, E. K. Silverman, P. Qasba, W. Gan, NHLBI Trans-Omics for Precision Medicine (TOPMed) Consortium, G. J. Papanicolaou, D. A. Nickerson, S. R. Browning, M. C. Zody, S. Zöllner, J. G. Wilson, L. A.

Cupples, C. C. Laurie, C. E. Jaquish, R. D. Hernandez, T. D. O'Connor, G. R. Abecasis, Sequencing of 53,831 diverse genomes from the NHLBI TOPMed Program. *Nature*. **590**, 290–299 (2021).

63. S. Das, L. Forer, S. Schönherr, C. Sidore, A. E. Locke, A. Kwong, S. I. Vrieze, E. Y. Chew, S. Levy, M. McGue, D. Schlessinger, D. Stambolian, P.-R. Loh, W. G. Iacono, A. Swaroop, L. J. Scott, F. Cucca, F. Kronenberg, M. Boehnke, G. R. Abecasis, C. Fuchsberger, Next-generation genotype imputation service and methods. *Nat. Genet.* **48**, 1284–1287 (2016).

64. C. C. Chang, C. C. Chow, L. C. Tellier, S. Vattikuti, S. M. Purcell, J. J. Lee, Second-generation PLINK: rising to the challenge of larger and richer datasets. *Gigascience*. **4**, 7 (2015).

65. Babraham Bioinformatics - FastQC A Quality Control tool for High Throughput Sequence Data, (available at https://www.bioinformatics.babraham.ac.uk/projects/fastqc/).

66. P. Ewels, M. Magnusson, S. Lundin, M. Käller, MultiQC: summarize analysis results for multiple tools and samples in a single report. *Bioinformatics*. **32**, 3047–3048 (2016).

67. BBMap. *SourceForge* (2022), (available at https://sourceforge.net/projects/bbmap/).

68. H. Li, R. Durbin, Fast and accurate short read alignment with Burrows-Wheeler transform. *Bioinformatics*. **25**, 1754–1760 (2009).

69. B. van de Geijn, G. McVicker, Y. Gilad, J. K. Pritchard, WASP: allele-specific software for robust molecular quantitative trait locus discovery. *Nat. Methods*. **12**, 1061–1063 (2015).

70. P. Danecek, J. K. Bonfield, J. Liddle, J. Marshall, V. Ohan, M. O. Pollard, A. Whitwham, T. Keane, S. A. McCarthy, R. M. Davies, H. Li, Twelve years of SAMtools and BCFtools. *Gigascience*. **10** (2021), doi:10.1093/gigascience/giab008.

71. A. R. Quinlan, I. M. Hall, BEDTools: a flexible suite of utilities for comparing genomic features. *Bioinformatics*. **26**, 841–842 (2010).

72. Y. Luo, B. C. Hitz, I. Gabdank, J. A. Hilton, M. S. Kagda, B. Lam, Z. Myers, P. Sud, J. Jou, K. Lin, U. K. Baymuradov, K. Graham, C. Litton, S. R. Miyasato, J. S. Strattan, O. Jolanki, J.-W. Lee, F. Y. Tanaka, P. Adenekan, E. O'Neill, J. M. Cherry, New developments on the Encyclopedia of DNA Elements (ENCODE) data portal. *Nucleic Acids Res.* **48**, D882–D889 (2020).

73. Picard, (available at http://broadinstitute.github.io/picard/).

74. P. Orchard, Y. Kyono, J. Hensley, J. O. Kitzman, S. C. J. Parker, Quantification, Dynamic Visualization, and Validation of Bias in ATAC-Seq Data with ataqv. *Cell Syst*. **10**, 298–306.e4 (2020).

75. G. Jun, M. Flickinger, K. N. Hetrick, J. M. Romm, K. F. Doheny, G. R. Abecasis, M. Boehnke, H. M. Kang, Detecting and estimating contamination of human DNA samples in sequencing and array-based genotype data. *Am. J. Hum. Genet.* **91**, 839–848 (2012).

76. A. T. L. Lun, G. K. Smyth, csaw: a Bioconductor package for differential binding analysis of ChIP-seq data using sliding windows. *Nucleic Acids Res.* **44**, e45 (2016).

77. K. D. Hansen, R. A. Irizarry, Z. Wu, Removing technical variability in RNA-seq data using conditional quantile normalization. *Biostatistics*. **13**, 204–216 (2012).

78. A. Dobin, C. A. Davis, F. Schlesinger, J. Drenkow, C. Zaleski, S. Jha, P. Batut, M. Chaisson, T. R. Gingeras, STAR: ultrafast universal RNA-seq aligner. *Bioinformatics*. **29**, 15–21 (2013).

79. Y. Liao, G. K. Smyth, W. Shi, The R package Rsubread is easier, faster, cheaper and better for alignment and quantification of RNA sequencing reads. *Nucleic Acids Res.* **47**, e47 (2019).

80. C. Soneson, M. I. Love, M. D. Robinson, Differential analyses for RNA-seq: transcript-level estimates improve gene-level inferences. *F1000Res.* **4**, 1521 (2015).

81. M. I. Love, W. Huber, S. Anders, Moderated estimation of fold change and dispersion for RNA-seq data with DESeq2. *Genome Biol.* **15**, 550 (2014).

82. J. Reimand, M. Kull, H. Peterson, J. Hansen, J. Vilo, g:Profiler—a web-based toolset for functional profiling of gene lists from large-scale experiments. *Nucleic Acids Res.* **35**, W193–W200 (2007).

83. H. Ogata, S. Goto, K. Sato, W. Fujibuchi, H. Bono, M. Kanehisa, KEGG: Kyoto Encyclopedia of Genes and Genomes. *Nucleic Acids Res.* **27**, 29–34 (1999).

84. D. Croft, G. O'Kelly, G. Wu, R. Haw, M. Gillespie, L. Matthews, M. Caudy, P. Garapati, G. Gopinath, B. Jassal, S. Jupe, I. Kalatskaya, S. Mahajan, B. May, N. Ndegwa, E. Schmidt, V. Shamovsky, C. Yung, E. Birney, H. Hermjakob, P. D'Eustachio, L. Stein, Reactome: a database of reactions, pathways and biological processes. *Nucleic Acids Res.* **39**, D691–D697 (2011).

85. J. A. Castro-Mondragon, R. Riudavets-Puig, I. Rauluseviciute, R. B. Lemma, L. Turchi, R. Blanc-Mathieu, J. Lucas, P. Boddie, A. Khan, N. Manosalva Pérez, O. Fornes, T. Y. Leung, A. Aguirre, F. Hammal, D. Schmelter, D. Baranasic, B. Ballester, A. Sandelin, B. Lenhard, K. Vandepoele, W. W. Wasserman, F. Parcy, A. Mathelier, JASPAR 2022: the 9th release of the open-access database of transcription factor binding profiles. *Nucleic Acids Res.* **50**, D165–D173 (2022).

86. K. S. Pollard, M. J. Hubisz, K. R. Rosenbloom, A. Siepel, Detection of nonneutral substitution rates on mammalian phylogenies. *Genome Res.* **20**, 110–121 (2010).

87. S. Gazal, H. K. Finucane, N. A. Furlotte, P.-R. Loh, P. F. Palamara, X. Liu, A. Schoech, B. Bulik-Sullivan, B. M. Neale, A. Gusev, Others, Linkage disequilibrium--dependent architecture of human complex traits shows action of negative selection. *Nat. Genet.* **49**, 1421 (2017).

88. J. Ernst, M. Kellis, ChromHMM: automating chromatin-state discovery and characterization. *Nat. Methods*. **9**, 215–216 (2012).

89. The International HapMap 3 Consortium, Integrating common and rare genetic variation in diverse human populations. *Nature*. **467**, 52–58 (2010).

90. 1000 Genomes Project Consortium, A. Auton, L. D. Brooks, R. M. Durbin, E. P.

Garrison, H. M. Kang, J. O. Korbel, J. L. Marchini, S. McCarthy, G. A. McVean, G. R. Abecasis, A global reference for human genetic variation. *Nature*. **526**, 68–74 (2015).

91. N. Kumasaka, A. J. Knights, D. J. Gaffney, Fine-mapping cellular QTLs with RASQUAL and ATAC-seq. *Nat. Genet.* **48**, 206–213 (2016).

92. Q. Q. Huang, S. C. Ritchie, M. Brozynska, M. Inouye, Power, false discovery rate and Winner's Curse in eQTL studies. *Nucleic Acids Res.* **46**, e133 (2018).

93. J. R. Davis, L. Fresard, D. A. Knowles, M. Pala, C. D. Bustamante, A. Battle, S. B. Montgomery, An Efficient Multiple-Testing Adjustment for eQTL Studies that Accounts for Linkage Disequilibrium between Variants. *Am. J. Hum. Genet.* **98**, 216–224 (2016).

94. A. S. E. Cuomo, G. Alvari, C. B. Azodi, single-cell eQTLGen consortium, D. J. McCarthy, M. J. Bonder, Optimizing expression quantitative trait locus mapping workflows for single-cell studies. *Genome Biol.* **22**, 188 (2021).

95. F. P. Casale, B. Rakitsch, C. Lippert, O. Stegle, Efficient set tests for the genetic analysis of correlated traits. *Nat. Methods*. **12**, 755–758 (2015).

96. Conomos, Gogarten, Brown, Chen, Rice, GENESIS: GENetic EStimation and Inference in Structured samples (GENESIS): Statistical methods for analyzing genetic data from samples with …. *Genesis*.

97. S. G. Coetzee, G. A. Coetzee, D. J. Hazelett, motifbreakR: an R/Bioconductor package for predicting variant effects at transcription factor binding sites. *Bioinformatics*. **31**, 3847–3849 (2015).

98. H. Touzet, J.-S. Varré, Efficient and accurate P-value computation for Position Weight Matrices. *Algorithms Mol. Biol.* **2**, 15 (2007).

99. P. Shannon, M. Richards, MotifDb: An Annotated Collection of Protein-DNA Binding Sequence Motifs, . R package version 1.37.1 (2022), doi:10.18129/B9.bioc.MotifDb.

100. C. Y. McLean, D. Bristor, M. Hiller, S. L. Clarke, B. T. Schaar, C. B. Lowe, A. M. Wenger, G. Bejerano, GREAT improves functional interpretation of cis-regulatory regions. *Nat. Biotechnol.* **28**, 495–501 (2010).

101. J. Yang, S. H. Lee, M. E. Goddard, P. M. Visscher, GCTA: a tool for genome-wide complex trait analysis. *Am. J. Hum. Genet.* **88**, 76–82 (2011).

102. J. Yang, T. Ferreira, A. P. Morris, S. E. Medland, Genetic Investigation of ANthropometric Traits (GIANT) Consortium, DIAbetes Genetics Replication And Meta-analysis (DIAGRAM) Consortium, P. A. F. Madden, A. C. Heath, N. G. Martin, G. W. Montgomery, M. N. Weedon, R. J. Loos, T. M. Frayling, M. I. McCarthy, J. N. Hirschhorn, M. E. Goddard, P. M. Visscher, Conditional and joint multiple-SNP analysis of GWAS summary statistics identifies additional variants influencing complex traits. *Nat. Genet.* **44**, 369–75, S1–3 (2012).

103. F. Hormozdiari, M. van de Bunt, A. V. Segrè, X. Li, J. W. J. Joo, M. Bilow, J. H. Sul, S. Sankararaman, B. Pasaniuc, E. Eskin, Colocalization of GWAS and eQTL Signals Detects Target Genes. *Am. J. Hum. Genet.* **99**, 1245–1260 (2016).

104. R. Bourgon, R. Gentleman, W. Huber, Independent filtering increases detection power for high-throughput experiments. *Proc. Natl. Acad. Sci. U. S. A.* **107**, 9546–9551 (2010).
